# Single‐cell RNA sequencing identify SDCBP in ACE2‐positive bronchial epithelial cells negatively correlates with COVID‐19 severity

**DOI:** 10.1111/jcmm.16714

**Published:** 2021-06-16

**Authors:** Ding Ma, Shuwen Liu, Lili Hu, Qinyu He, Weiwei Shi, Dongliang Yan, Yin Cao, Guang Zhang, Zhongxia Wang, Junhua Wu, Chunping Jiang

**Affiliations:** ^1^ Jiangsu Key Laboratory of Molecular Medicine National Institute of Healthcare Data Science at Nanjing University Medical School of Nanjing University Nanjing China; ^2^ Department of Hepatobiliary Surgery The Affiliated Drum Tower Hospital of Nanjing University Medical School Nanjing China; ^3^ Department of Hepatobiliary Surgery Drum Tower Clinical College of Nanjing Medical University Nanjing China

**Keywords:** ACE2, antigen processing and presentation, COVID‐19, HuRI (human reference interactome), SDCBP (Syndecan‐Binding Protein), WGCNA (weighted gene co expression network analysis)

## Abstract

The coronavirus disease 2019 (COVID‐19), caused by the novel severe acute respiratory syndrome coronavirus 2 (SARS‐CoV‐2), has resulted in many deaths throughout the world. It is vital to identify the novel prognostic biomarkers and therapeutic targets to assist with the subsequent diagnosis and treatment plan to mitigate the expansion of COVID‐19. Since angiotensin‐converting enzyme 2 (ACE2)‐positive cells are hosts for COVID‐19, we focussed on this cell type to explore the underlying mechanisms of COVID‐19. In this study, we identified that ACE2‐positive cells from the bronchoalveolar lavage fluid (BALF) of patients with COVID‐19 belong to bronchial epithelial cells. Comparing with patients of COVID‐19 showing severe symptoms, the antigen processing and presentation pathway was increased and 12 typical genes, HLA‐DRB5, HLA‐DRB1, CD74, HLA‐DRA, HLA‐DPA1, HLA‐DQA1, HSP90AA1, HSP90AB1, HLA‐DPB1, HLA‐DQB1, HLA‐DQA2, and HLA‐DMA, particularly HLA‐DPB1, were obviously up‐regulated in ACE2‐positive bronchial epithelial cells of patients with mild disease. We further discovered SDCBP was positively correlated with above 12 genes particularly with HLA‐DPB1 in ACE2‐positive bronchial epithelial cells of COVID‐19 patients. Moreover, SDCBP may increase the immune infiltration of B cells, CD8+ T cells, CD4+ T cells, macrophages, neutrophils and dendritic cells in different lung carcinoma. Moreover, we found the expression of SDCBP was positively correlated with the expression of antigen processing and presentation genes in post‐mortem lung biopsies tissues, which is consistent with previous discoveries. These results suggest that SDCBP has good potential to be further developed as a novel diagnostic and therapeutic target in the treatment of COVID‐19.

## 1 INTRODUCTION

Since December 2019, the outbreak of a novel coronavirus, which causes coronavirus disease 2019 (COVID‐19), has brought terrible suffering to patients worldwide and has resulted in not only continuously increasing mortality rates but also considerable economic losses.^1^, ^2^ The patients who initially develop fever or respiratory symptoms can then be divided into a mild group and a severe group based on the severity of their symptoms. A large proportion of patients with the common mild disease were classified into the mild group, and approximately 15% to 20% of the patients were classified into the severe group and needed to receive oxygenation as part of their adjuvant therapy. Although all patients need to receive a good standard of care, those belonging to the severe group place the highest pressure on doctors.^3^ Thus, identification of the differences in gene expression between the patients in the mild group and those in the severe group is necessary. We are committed to discover the differentially expressed genes (DEGs) between patients with mild symptoms and those with severe symptoms and promoting the development of a treatment for COVID‐19 in a targeted manner.

The causative agent of severe acute respiratory syndrome coronavirus (SARS‐CoV) enters cells with angiotensin‐converting enzyme 2 (ACE2), the receptor of SARS‐CoV.^4^ A Western blot analysis of mCherry‐ or ACE2‐expressing A549 cells infected with SARS‐CoV‐2 indicated that the SARS‐CoV‐2 nucleocapsid (N) is only found on ACE2‐expressing A549 cells.^5^ ACE2‐positive cells can be considered susceptible to exposure to SARS‐CoV‐2 due to the significant blocking effect of human recombinant soluble ACE2 at the early stage of COVID‐19 infection.^6^, ^7^, ^8^ Soeren Lukassen et al^9^ have discovered that SARS‐CoV‐2 receptor ACE2 and the transmembrane protease serines (TMPRSS2) are primarily expressed in bronchial transient secretory cells. Those found remind that bronchial epithelial cells with ACE2‐positive expression have a chance to become the first target cells of SARS‐CoV‐2 infection in the lung.

The physiological response after viral infection is usually manifested by viral replication and subsequent changes at the cellular level.^10^ Once the virus enters and infects host cells, the infected cells detect the presence of viral replication via a number of pattern recognition receptors (PRRs). In the context of viral infection, the detection of viral replication is mediated to a large extent by an intracellular PRR family that identifies abnormal RNA structures that typically form during viral replication.^11^ Human leucocyte antigen (HLA), which is the major pattern recognition receptor in humans, is related to antigen processing and presentation pathways. Notably, bronchial epithelial cells exhibit similar antigen presentation and processing properties.^12^

The current research on COVID‐19 is focussing on two major problems that remain to be resolved: the mechanism through which COVID‐19 results in the development of mild and severe symptoms remains unclear,^13^, ^14^ and effective therapeutic targets for COVID‐19 have not been discovered.^13^ We aim to explore the internal mechanism that leads to the development of mild or severe COVID‐19 symptoms through a comprehensive analysis of the gene expression profile of ACE2‐positive bronchial epithelial cells and to identify new genes that can serve as therapeutic targets.

To compare the transcriptional response of patients with mild and severe COVID‐19 symptoms, the transcriptional signals that might serve as the biological basis for COVID‐19 were identified. We downloaded a single‐cell RNA sequencing data set (GSE145926) from the Gene Expression Omnibus database that contains information on cells from the bronchoalveolar lavage fluid (BALF) in six patients with severe COVID‐19 and three patients with mild COVID‐19. We then selected cells with ACE2 expression levels greater than 0 as ACE2‐positive cells, and these cells were subsequently annotated as bronchial epithelial cells using SingleR.^15^ We subsequently used these cells to identify the DEGs between patients with mild disease and those with severe disease and constructed a coexpression network through weighted gene coexpression network analysis (WGCNA) to identify the modules that are most important for antigen processing and presentation pathways. We also performed a human reference interaction (HuRI) analysis to determine the unbiased relationship between the above‐mentioned pathways and modules (http://interactome‐atlas.org). The obtained results were then verified by gene expression profiling interactive analysis (GEPIA), using the tumour immune estimation resource (TIMER) and through Pearson's correlation coefficients.

## 2 MATERIALS AND METHODS

### 2.1 Data processing

We downloaded GSE145926 scRNA‐seq submitted by Zheng Zhang et al from the Gene Expression Omnibus database (https://www.ncbi.nlm.nih.gov/geo/query/acc.cgi?acc=GSE145926). It contains the read count matrix of the COVID‐19 patients and healthy control. H5 expression data were read using the Read10X_h5 function (Seurat package). Nine cases were selected for analysis, and these included three cases of mild disease and six cases of severe disease. All ACE2‐positive cells (count>0) were selected using the subset function (dplyr package) in R software, and unique cells with molecules detected count (nCount) > 75 000 and unique cells with percent.mt (percentage of mitochondrial genes) values greater than 40 were subsequently filtered out (Seurat package).

### 2.2 Dimensionality reduction and clustering

The first 2000 genes with the most significant differences in expression were obtained using the FindVariableFeatures function (Seurat package). We applied a linear transformation function, the ScaleDate function (Seurat package), to preprocess the data, which is an essential step in data standardization before the application of a dimensionality reduction technique, such as principal component analysis (PCA). We then performed a PCA of the scaled data using the RunPCA function (Seurat package) and clustered the cells using the FindNeighbors and FindClusters functions (Seurat package). We subsequently utilized the UMAP algorithm for dimensionality reduction to visualize these data sets and make them suitable for exploration.

### 2.3 Cell type annotation of single cells

We used the SingleR (Single‐cell Recognition) function (SingleR package) to annotate single‐cell RNA‐seq clusters based on Human Primary Cell Atlas data, which is a reference data set of samples with known labels. Based on similarity to the above‐mentioned reference set, the ACE2‐positive cells were labelled from the test data set.

### 2.4 Biomarker genes that showed differential expression between the mild and severe groups

We identified markers of the mild group compared with the severe group using the Find All Markers function (Seurat package). These biomarkers were detected in cells with at least 25% gene expression in both populations, regardless of the level of gene expression.

### 2.5 Gene enrichment analysis

Using the gseaGO and gseaKEGG functions (clusterProfiler package), we performed Gene Ontology (GO) and Kyoto Encyclopedia of Genes and Genomes (KEGG) pathway analyses of the differentially expressed biomarker genes.

### 2.6 Construction of the co‐expression module

First, the usability of 5000 genes was evaluated, and the adjacency matrix A_mn_ was defined as follows:

$$A_{\text{mn}}={\left|S_{\text{mn}}\right|}^{\beta },$$

where S_mn_ was Pearson's correlation coefficient between gene m and gene n and Aij was the contiguity between gene m and gene n. We selected *β* = 5 as the soft‐thresholding parameter (scale‐free *R*2 = 0.88). We then transformed the adjacency matrix into a topological overlap matrix (TOM), and genes with high absolute correlation values were divided into different gene modules according to dissimilarity measures based on the TOM through average linkage hierarchical clustering with a minimum of 30.

### 2.7 Identification of modules

We calculated the correlations between the module eigengenes (the major component in the principal component analysis for each gene module) and antigen processing and presentation genes to measure the significance of each module. The module significance (MS) was defined as the average absolute gene significance measured for all the genes in a given module. In general, the module with the highest absolute MS was considered to exhibit the strongest link between given genes.

### 2.8 Direct genetic association identification

We downloaded the HuRI unions from http://www.interactome‐atlas.org/ and then analysed the PPIs in modules with high absolute biological significance. Subsequently, the genes in the modules that are most directly related to various important biological functions were acquired.

### 2.9 Validation of the genes with the most direct connections

GSE147143 was downloaded for further validation. We compared the association of SDCBP expression with antigen processing and presentation gene expression in bronchoalveolar lavage fluid (BALF) (GSE147143). Moreover, the association of SDCBP expression with antigen processing and presentation gene expression in lung adenocarcinoma (TCGA‐LUAD), lung squamous cell carcinoma (TCGA‐LUSC) and normal lung tissue (GTEx‐lung) was analysed by GEPIA (http://gepia.cancer‐pku.cn/), and the correlations between immune cell types and the expression of SDCBP in lung adenocarcinoma (TCGA‐LUAD) and lung squamous cell carcinoma (TCGA‐LUSC) were analysed by TIMER (http://cistrome.dfci.harvard.edu/TIMER/).

## 3 RESULTS

### 3.1 Extraction of ACE2‐positive cells and identification of cellular information

**FIGURE 1:**
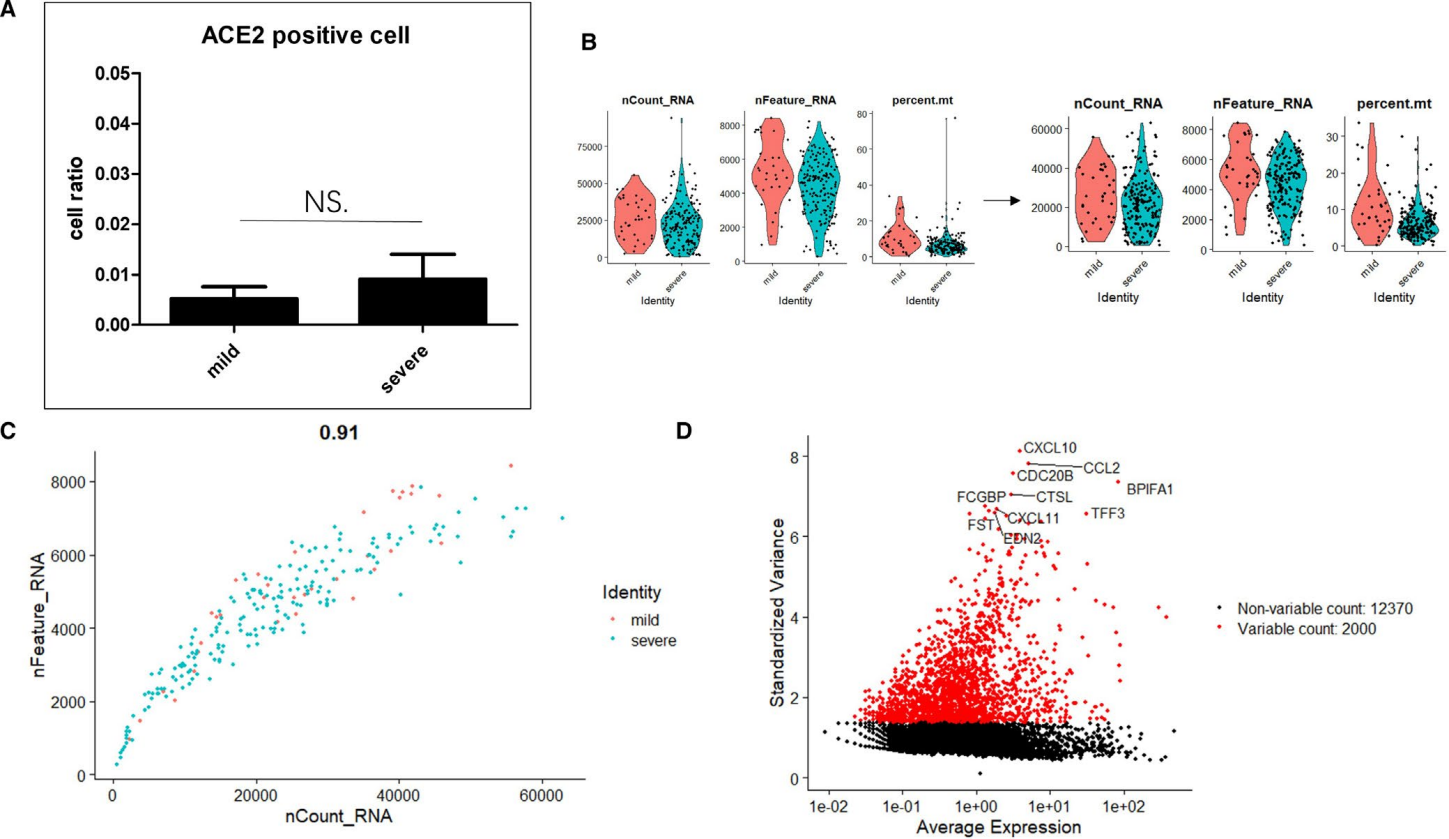
Identification information of ACE2‐positive cells based on scRNA‐seq data. A, Ratio of ACE2+ cells in the BALF from patients with mild disease to those in the BALF from patients with severe disease. B, Total gene counts (nCount), number of gene types (nFeature) and percentage of mitochondrial genes (percent.mt) in the mild and severe groups. Each dot represents an ACE2‐positive bronchial epithelial cell. C, Relationship between nCount and nFeature. D, A subset of features that shows high variation between cells (ie, genes that are highly expressed in some cells and lowly expressed in others) was calculated. Red dot: top 2000 genes with relatively significant differential expression, and the top 10 genes with the most significant variance are marked

In this study, we obtained single‐cell sequencing data from nine cases in the GSE145926 data set (Table 1), selected the ACE2‐positive cells (count>0) from BALF and calculated their percentage. No significant difference in the ACE2‐positive cell percentage was found between the mild and severe groups (Figure 1A), which indicates that the severity of COVID‐19 does not depend on the proportion of ACE2‐positive cells. We calculated the number of gene types (nFeature) presented in the ACE2‐positive cell, nCount and the percentage of reads that mitochondrial genome (percent.mt) and then filtered the cells with nCount greater than 75 000 and those with a percent.mt value higher than 40 (Figure 1B). 222 ACE2‐positive cells were picked out and used for subsequent analysis. The two groups showed consistent trends in nCount and nFeature, which means the feasibility of single‐cell sequencing (Figure 1C). To categorize ACE2‐positive cells, we then identified a subset of characteristics showing high intercellular variation in the data set and labelled the top 10 genes with the most significant differences (Figure 1D). It is known that SARS‐CoV employs the host cell proteases TMPRSS2 for the viral spike (S) proteins priming in human lung cells,^4^ and interestingly, we discovered that out of 222 ACE2‐positive cells, 96 ones express TMPRSS2 (Table S1).

**TABLE 1:** Cell characteristics of patients with COVID‐19

Title	Source	Cell subsets	Patient group	Cell number	ACE2‐positive cell number
BALF, C141 (scRNA‐seq)	BALF	Total cell	Mild	4233	16
BALF, C142 (scRNA‐seq)	BALF	Total cell	Mild	4562	9
BALF, C144 (scRNA‐seq)	BALF	Total cell	Mild	915	9
BALF, C143 (scRNA‐seq)	BALF	Total cell	Severe	20 289	23
BALF, C145 (scRNA‐seq)	BALF	Total cell	Severe	17 396	24
BALF, C146 (scRNA‐seq)	BALF	Total cell	Severe	3401	2
C148 (scRNA‐seq)	BALF	Total cell	Severe	2397	70
C149 (scRNA‐seq)	BALF	Total cell	Severe	2485	47
C152 (scRNA‐seq)	BALF	Total cell	Severe	6902	24

### 3.2 The relationship between 222 ACE2‐positive cells and bronchial epithelial cells

**FIGURE 2:**
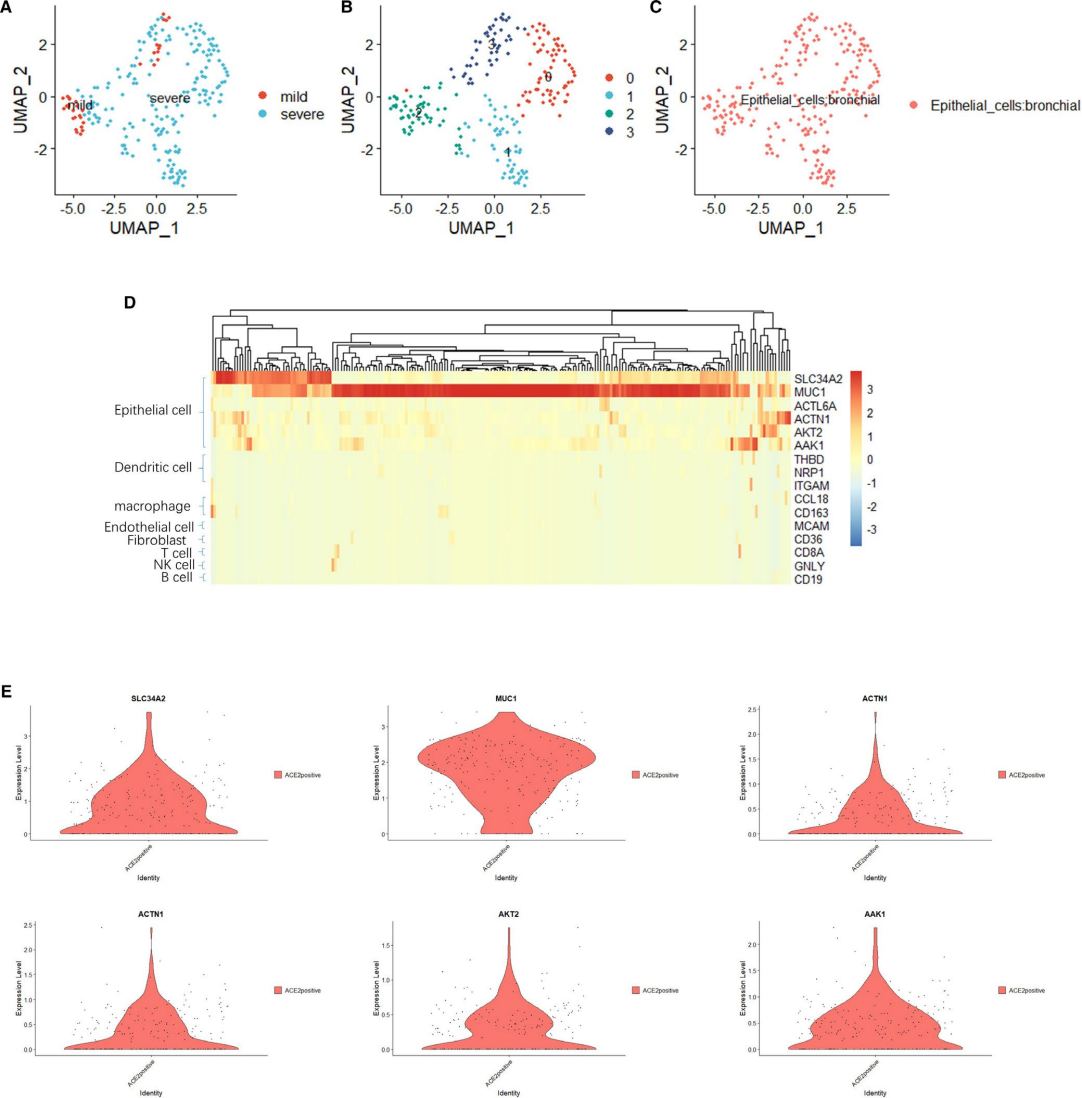
ACE2‐positive cells belong to bronchial epithelial cells. A, u‐MAP plot of approximately 222 ACE2‐positive cells in the mild and severe groups. B, The 222 ACE2‐positive cells were divided into four clusters after further UMAP dimensionality reduction, and the different clusters are shown in different colors. C, The cells in the given subgroups are shown in different colors based on the prediction results obtained with SingleR. D, The heatmap of cell marker in 222 ACE2‐positive cells. E, The expression of epithelial cell marker in 222 ACE2‐positive cells

We created a cell‐by‐gene expression matrix and performed dimensionality reduction by uniform manifold approximation and projection (UMAP) for further clustering analysis/(Figure 2A).^16^ Clusters were then further identified according to the different types of cell expression through graph‐based clustering, and four clusters were ultimately obtained using the Seurat package (Figure 2B).^15^ It is observed that the gene expression features of bronchial epithelial cells between mild and severe patients have different distribution in above four populations, indicating certain expression differences between bronchial epithelial cells from mild and severe patients. Interestingly, we discovered that all ACE2‐positive cells belonged to bronchial epithelial cells by SingleR using the Human Primary Cell Atlas as the reference data set^17^ (Figure 2C). Furthermore, we used canonical markers (Table S2), especially MUC1 and SLC34A2, to match the ACE2‐positive cells to known cell types, and gene expression profiles were visualized by heat map (Figure 2D) and the epithelial cell markers were visualized by violin plot (Figure 2A‐E),^18^, ^19^, ^20^, ^21^, ^22^, ^23^, ^24^, ^25^, ^26^ which confirmed that all ACE2‐positive cells belonged to bronchial epithelial cells.

### 3.3 Antigen processing and presentation genes were significantly up‐regulated in ACE2‐positive bronchial epithelial cells of patients with mild disease

**FIGURE 3:**
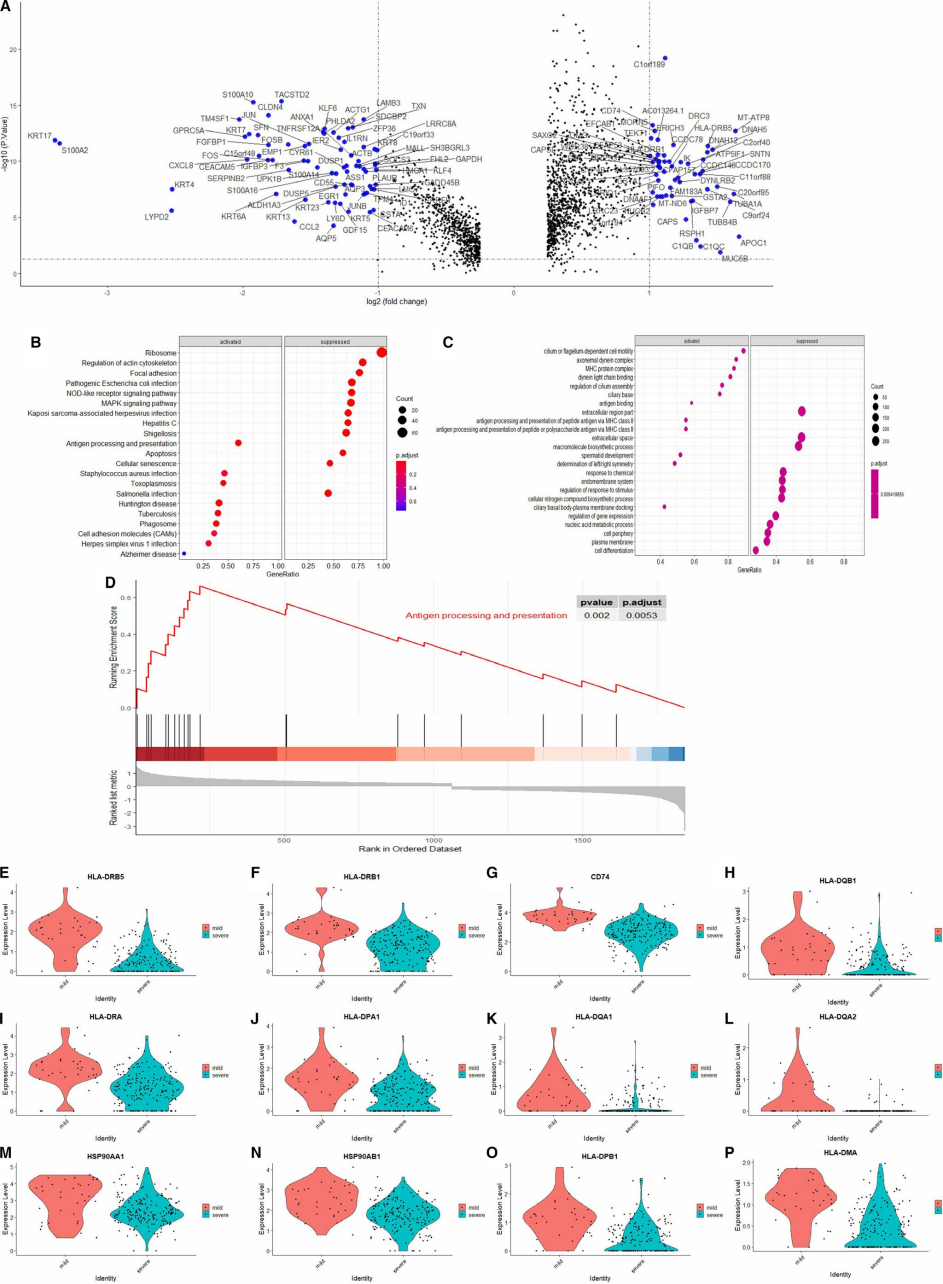
Identification of differentially expressed genes between ACE2‐positive bronchial epithelial cells of patients with mild disease compared with those with severe disease based on scRNA‐seq data. A, Volcano plot of 1925 differentially expressed genes. B, KEGG pathway analysis C, Gene Ontology analysis, D, Top enriched pathways in ACE2‐positive bronchial epithelial cells from patients with mild disease. E‐P, Violin plots of the differential expression of HLA‐DRB5, HLA‐DRB1, CD74, HLA‐DRA, HLA‐DPA1, HLA‐DQA1, HSP90AA1, HSP90AB1, HLA‐DPB1, HLA‐DQB1, HLA‐DQA2, and HLA‐DMA. Each dot represents an ACE2‐positive bronchial epithelial cell

The cluster results showed that the main components of the ACE2‐positive cells of patients with mild and severe disease exhibited significant differences, which hinted at further exploration of DEGs. We then identified 1925 DEGs by comparing cells from mild cases with those from severe cases (Figure 3A) (Table S3). To further elucidate the functions, signalling pathways and upstream regulators of the DEGs, KEGG and GO enrichment analyses were performed, and the results indicated that the DEGs were mainly enriched in antigen processing and presentation pathway (Figure 3B,C).^27^ We then further verified the top enriched KEGG pathways by GSEA and discovered that 12 genes, including HLA‐DRB5, HLA‐DRB1, CD74, HLA‐DRA, HLA‐DPA1, HLA‐DQA1, HSP90AA1, HSP90AB1, HLA‐DPB1, HLA‐DQB1, HLA‐DQA2, and HLA‐DMA, in antigen processing and presentation pathway were typically up‐regulated in patients with mild disease (Figure 3D, Figure S1)^28^(Figure 3E‐P). HLA‐DRB5, HLA‐DRB1, CD74, HLA‐DRA, HLA‐DPA1, HLA‐DQA1, HLA‐DPB1, HLA‐DQB1, HLA‐DQA2 and HLA‐DMA belong to HLA‐Ⅱ family which widely distribute in APCs, B cells, macrophages and dendritic cells, responsible for antigen presentation and immune regulation.^29^ Besides, heat shock proteins (HSPs), HSP90AA1 and HSP90AB1, are also closely related to multiple immune processes.^30^, ^31^ These DEGs between the mild and severe groups might be key to determining the severity of COVID‐19.

### 3.4 Correlation of SDCBP with the expression of antigen processing and presentation genes in bronchial epithelial cells from COVID‐19 patients

**FIGURE 4:**
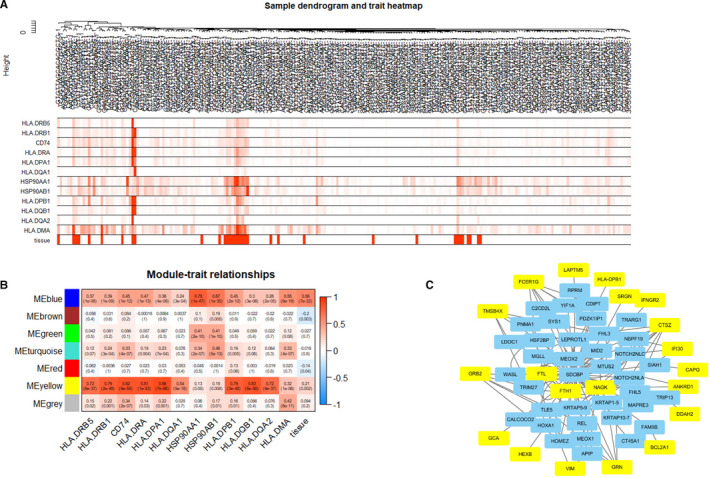
Relationship between SDCBP and typical genes in the antigen processing and presentation pathway. A, Clustering dendrogram of 224 bronchial epithelial cells. B, Heatmap of the correlations between module eigengenes and the expression of antigen processing and presentation genes. C, PPIs between SDCBP and 20 typical genes in the yellow module based on the HuRI atlas. The yellow squares represent genes belonging to the yellow module in B, and the blue squares show the pivotal genes with PPIs between SDCBP and genes in the yellow module

For the exploration of prognostic markers and therapeutic targets, we built coexpression networks through a WGCNA of bronchial epithelial cell gene expression in data sets of the mild and severe patient groups described in earlier parts of the study.^32^ The dendrogram obtained from the cell sample clustering using the average linkage method indicated that the microarray is feasible (Figure 4A). A significant difference in the distribution of gene expression related to antigen processing and presentation was found, and these genes were concentrated in the samples from the mild group, which further indicates that these genes are associated with disease severity (Figure 4B).

We selected *β* = 5 as the soft‐thresholding power (scale‐free *R* ^2^ = 0.88; Figure S2A‐B), and seven modules were then screened by average linkage hierarchical clustering (Figure S2C). Our findings showed a strong link between the genes in the yellow module and the genes in antigen processing and presentation pathways (Figure 4B). We further analysed the yellow module and found 83 genes (Figure S2D‐E) (Table S4). Using the HuRI atlas as the reference, we found that SDCBP (SDCBP genes or SDCBP‐protein A‐genes confirmed by Y2H assay) has direct or indirect interaction with 20 of the 83 proteins and exhibited most PPIs with proteins in the yellow module (Figure 4C).^33^ These indicate that SDCBP plays a potential role in human antigen presentation in bronchial epithelial cells from COVID‐19 patients.

### 3.5 The relationship between SDCBP and antigen processing and presentation genes in lung tissue

**FIGURE 5:**
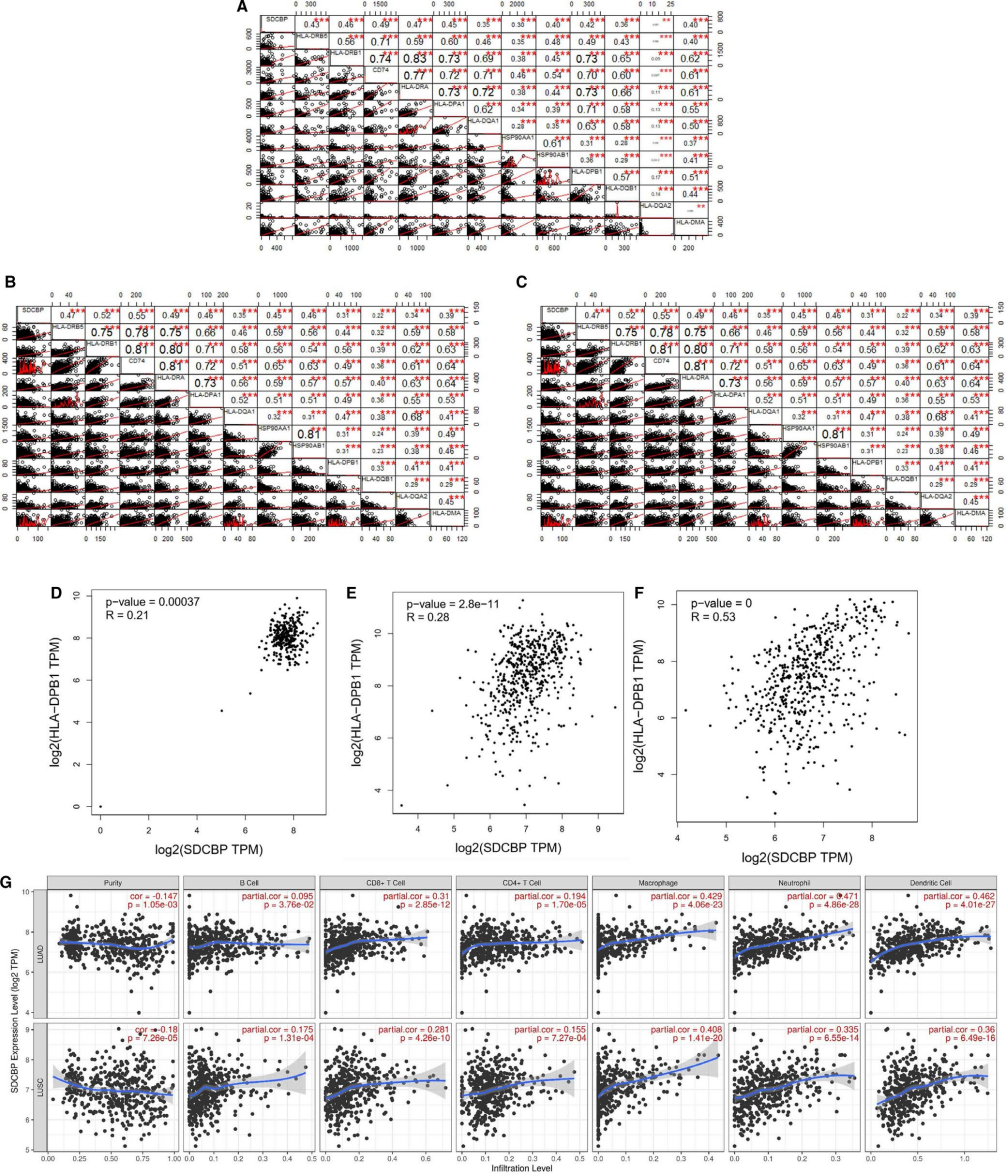
Relationship between SDCBP and the expression of antigen processing and presentation genes in BALF and lung tissue. A‐C, Correlation analysis (Pearson's correlation coefficients) between the expression of SDCBP and the expression of antigen processing and presentation genes based on GSE147143. Each circle in the lower left area represents a BALF cell, and the short red line represents the regression curve. Each datum in the upper right square represents the regression coefficient of the correlation relationship. (*P < .05, **P < .01, ***P < .001) D. Correlation analysis between the expression of SDCBP and HLA‐DPB1 based on normal lung tissue data from GTEx. E, Correlation analysis between the expression of SDCBP and HLA‐DPB1 based on the TCGA‐LUAD data. F, Correlation analysis between the expression of SDCBP and HLA‐DPB1 based on the TCGA‐LUSC data. G, Separate correlation analyses between the expression of SDCBP and B cells, CD8+ T cells, CD4+ T cells, macrophages, neutrophils, and dendritic cells based on the TCGA‐LUAD and TCGA‐LUSC data

Our findings were confirmed using the validation data set GSE147143, among the COVID‐19 samples, and macrophages, B cells, endothelial cells, ciliated cells, club cells, as well as type1 and type2 alveolar cells (AT1 and AT2) were included. Results indicated that the expression level of SDCBP was positively correlated with that of genes related to antigen processing and presentation (Figure 5A‐C) in lung tissue from COVID‐19 patients. Since we have identified the positive correlation at the single‐cell level, we then verified this kind of relationship at tissue level. Through further analysis of data sets from The Cancer Genome Atlas (TCGA) and Genotype‐Tissue Expression (GTEx)^34^, ^35^ of different lung tissues, including adenocarcinoma, lung squamous cell carcinoma and normal lung tissues, we verified significantly positive correlations between SDCBP and HLA‐DRB5, HLA‐DRB1, CD74, HLA‐DRA, HLA‐DPA1, HLA‐DQA1, HSP90AA1, HSP90AB1, HLA‐DPB1, HLA‐DQB1, HLA‐DQA2 and HLA‐DMA, particularly that between SDCBP and HLA‐DPB1 (Figure S3, Figure 5D‐F).^36^ We also found that the increase in the SDCBP gene expression level was significantly associated with the increase in the canonical markers of B cells, CD8+ T cells, CD4+ T cells, macrophages, neutrophils and dendritic cells in LUSC and LUAD (Figure 5G). All those cell markers are precisely provided by TIMER.^37^ The analysed results suggested that SDCBP is positively correlated with antigen processing and presentation genes in lung tissues.

### 3.6 The relationship between SDCBP expression and antigen processing and presentation genes in epithelial cells of post‐mortem lung biopsies epithelial cells from patients die of COVID‐19

**FIGURE 6:**
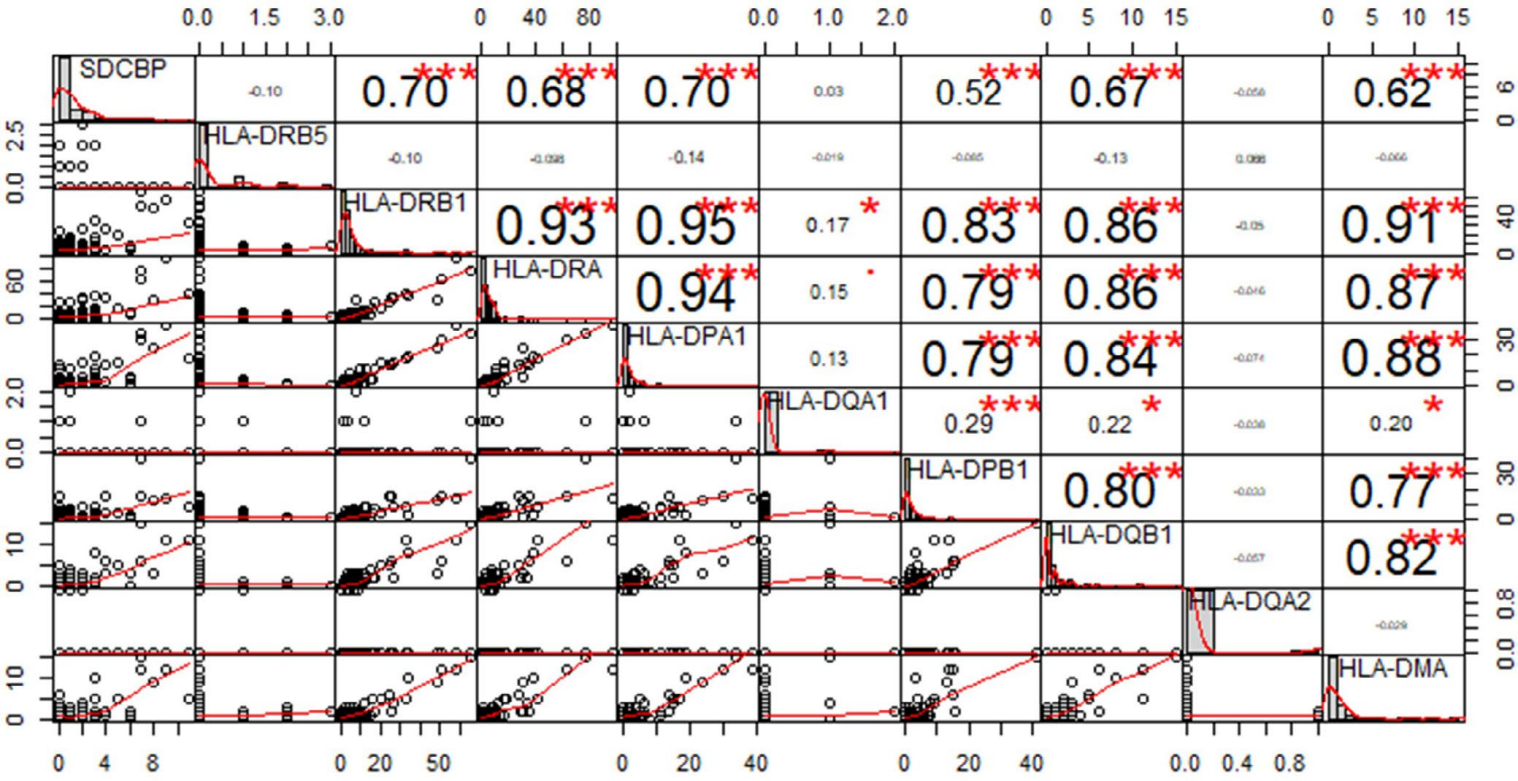
Relationship between SDCBP and the expression of antigen processing and presentation genes in epithelial cell. A, Correlation analysis (Pearson's correlation coefficients) between the expression of SDCBP and the expression of antigen processing and presentation genes based on GSE158127. Each circle in the lower left area represents a ACE2 positive cell, and the short red line represents the regression curve. Each datum in the upper right square represents the regression coefficient

For some patients with sincerely severe symptoms, inevitable death was known as the worst outcome. We downloaded the data about two post‐mortem lung biopsies PB1 and PB2 from the validation data set GSE158127 (https://www.ncbi.nlm.nih.gov/geo/query/acc.cgi?acc=GSE158127). We picked only ACE2‐positive cells therefrom for further analysis. We also use canonical markers (Table S2) to match the ACE2‐positive cells to known cell types (Table [Link], [Link]), from which we discovered that SDCBP was positively correlated with the antigen processing and presentation genes (Figure 6), such as HLA‐DPB1, HLA‐DRB1, HLA‐DRA, HLA‐DQB1, HLA‐DMA. Thus, there is a suspect that SDCBP might be a new target in the treatment of COVID‐19, which could provide new ideas for achieving a good prognosis.

## 4 DISCUSSION

We selected ACE2‐positive bronchial epithelial cells from BALF for analysis in this study, and our results provide evidence showing that the expression of antigen processing and presentation genes, particularly HLA class Ⅱ, is significantly increased in patients with mild disease. Furthermore, based on the above‐mentioned findings, we identified SDCBP as the target for the prediction and subsequent treatment of COVID‐19 because this compound plays a vital role in the positive regulation of HLA class II. On the one hand, all of these results reveal a new target for therapeutic intervention and thus provide instructional implications for understanding the pathogenesis of COVID‐19; on the other hand, understanding the antigen presentation function of ACE2‐positive cells in the bronchial epithelium is vital and can be considered as a future direction in the research and treatment of COVID‐19.

Zhou et al^38^ and Hoffmann et al^4^ identified ACE2 as a major receptor of COVID‐19,^4^, ^38^, ^39^ and these findings suggest that understanding ACE2‐positive cells should be key for improving the treatment and prognosis of patients with COVID‐19. Airborne transmission is a common and direct route of COVID‐19 infection. Through the interaction with its cellular receptor ACE2, SARS‐COV‐2 can enter and replicate in airway bronchial epithelial cells,^40^ which explains why most patients exhibit lung infections, and as a result, ACE2‐positive bronchial epithelial cells in the alveolar lavage fluid were selected in our study.

To date, there is no evidence of any effective treatment for severe COVID‐19. Although many therapies have been proposed, quarantine is the only intervention that appears to be effective.^41^ Thus, there is an urgent need to find new treatments.

Patients with mild disease tend to quickly recover without any therapy, but those with severe disease have difficulty recovering even if they receive oxygenation as part of their adjuvant therapy,^3^ which suggests that the investigation of differences between mild and severe cases might allow the development of new treatments. Prabhu S Arunachalam et al^42^ observed reduced expression of human leucocyte antigen class DR (HLA‐DR) and pro‐inflammatory cytokines in myeloid cells among the peripheral blood mononuclear cell (PBMC) population from patients with COVID‐19. The changes in PBMCs might be due to changes in bronchial epithelial cells. All the above‐mentioned information provides insight into the importance of distinguishing the bronchial epithelial cells of patients with mild diseases from those of patients with severe disease at the molecular and gene levels.

The overexpression of HLA class II genes in PBMCs is associated with a good prognosis.^42^ Aaron J Wilk et al^43^ discovered that the down‐regulation of HLA class Ⅱ in monocytes and B cells is closely related to the number of plasmablasts produced by patients with acute respiratory failure who had to rely on mechanical ventilation strategies. This finding suggests that the dysregulation of presenting antigens might lead to the aggravation of infection. Another meaningful finding was reported by Evangelos J. Giamarellos‐Bourboulis et al^44^, who discovered that the expression of HLA‐DR is inhibited in the plasma of patients with severe respiratory failure due to COVID‐19. Due to a lack of virus‐specific immunity, a strong innate immune dysregulation known as a “cytokine storm” leads to the aggravation of COVID‐19 infections.^45^ Notably, the H1N1 influenza pandemic and the H5N1 outbreak of highly pathogenic avian influenza have shown that some patients infected with the virus die not from the infection itself but from overimmunity.^46^ A Elssner et al^47^ proved that the up‐regulation of human leucocyte antigen (HLA) Ⅱ in bronchial epithelial cells (BECs) is related to the rejection response. Bronchial epithelial cells are regarded as the first line of defence in terms of recognizing antigenic peptides and presenting antigens. These findings support our hypothesis that the antigen presentation‐related genes, including HLA‐Ⅱ, show higher expression in the ACE2‐positive bronchial epithelial cells of patients with mild disease than in those from patients with severe disease.

SDCBP is a PDZ domain‐containing adaptor protein that can influence the trafficking of transmembrane proteins^48^ but also reportedly regulates the assembly of signalling complexes, including signalling downstream of Toll‐like receptors(TLRs)^49^. In this study, SDCBP‐expressing cells belong to epithelial cell; we found the expression of canonical airway epithelial and immune cell markers in SDCBP‐expressing cells (Table S7). WGCNA^32^ can be used to find clusters (modules) of highly correlated genes. The relationship between genes has been verified by a series of related studies. HuRI provides binary protein‐protein interactions^33^ between 17 500 tested proteins that have been verified by Y2H assays.

The present study provides a new prognosis and therapy target for COVID‐19 through the use of WGCNA and HuRI: SDCBP. However, further in vivo and in vitro studies are necessary to clarify the molecular mechanisms associated with SDCBP or HLA Ⅱ. Because the global COVID‐19 epidemic remains at the pandemic phase, any further progression in elucidating the viral infection mechanism might be valuable to vaccine development.

## CONFLICT OF INTEREST

The authors declare that there are no conflicts of interest.

## AUTHOR CONTRIBUTION

**Ding Ma:** Conceptualization (equal); Data curation (equal); Formal analysis (equal); Investigation (equal); Methodology (equal); Project administration (equal); Resources (equal); Software (equal); Supervision (equal); Validation (equal); Visualization (equal); Writing‐original draft (equal); Writing‐review & editing (equal). **Shuwen Liu:** Investigation (equal); Project administration (equal); Resources (equal); Supervision (equal); Validation (equal); Writing‐original draft (equal); Writing‐review & editing (equal). **Lili Hu:** Project administration (equal); Resources (equal); Validation (equal). **Qingyu He:** Project administration (equal); Validation (equal). **Weiwei Shi:** Project administration (equal); Supervision (equal); Validation (equal). **Dongliang Yan:** Validation (equal). **Yin Cao:** Validation (equal). **Guang Zhang:** Validation (equal). **Zhongxia Wang:** Formal analysis (equal); Funding acquisition (equal); Project administration (equal); Supervision (equal); Validation (equal); Writing‐review & editing (equal). **Chunping Jiang:** Conceptualization (equal); Funding acquisition (equal); Project administration (equal); Resources (equal); Supervision (equal); Validation (equal); Writing‐review & editing (equal). **Junhua Wu:** Conceptualization (equal); Funding acquisition (equal); Methodology (equal); Project administration (equal); Supervision (equal); Validation (equal); Writing‐review & editing (equal).

## Supporting information

Figure S1

Figure S2

Figure S3

Table S1

Table S2

Table S3

Table S4

Table S5

Table S6

Table S7

## Data Availability

All the data used in this study were downloaded from public databases as stated in the manuscript.
