# Correction to Single‐cell RNA sequencing identify SDCBP in ACE2‐positive bronchial epithelial cells negatively correlates with COVID‐19 severity

**DOI:** 10.1111/jcmm.17778

**Published:** 2023-06-10

In Ding Ma et al,^1^ an error occurred in Figure 2 Panel E, and the plot on the top right is a duplication of the plot of ACTN1 on the lower left. And another error occurred in Figure 5 Panel C, the correlogram is a duplication of the Figure 5 Panel B. Correct version of Figure 2 (the top right of Panel E) and Figure 5 (Panel C) should have been as depicted below:

**FIGURE 2:**
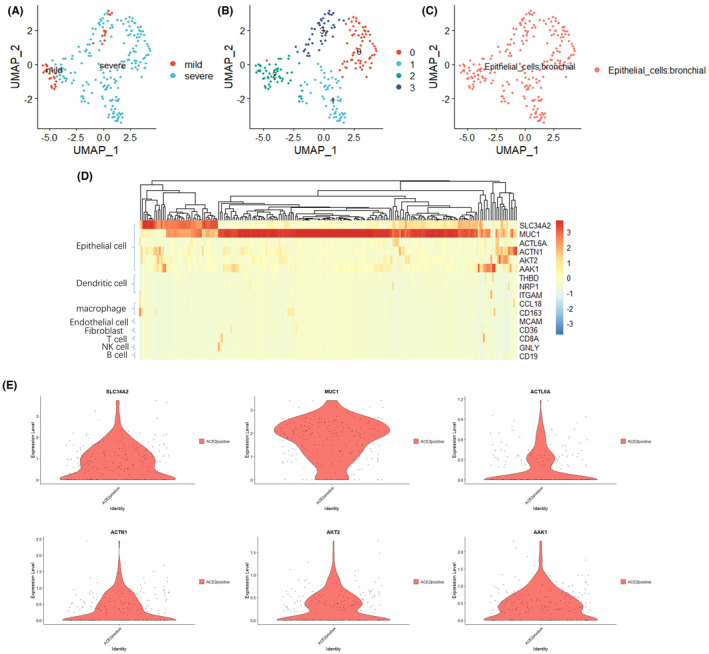
ACE2‐positive cells belong to bronchial epithelial cells. (A) u‐MAP plot of approximately 222 ACE2‐positive cells in the mild and severe groups. (B) The 222 ACE2‐positive cells were divided into four clusters after further UMAP dimensionality reduction, and the different clusters are shown in different colours. (C) The cells in the given subgroups are shown in different colours based on the prediction results obtained with SingleR. (D) The heatmap of cell marker in 222 ACE2‐positive cells. (E) The expression of epithelial cell marker in 222 ACE2‐positive cells.

**FIGURE 5:**
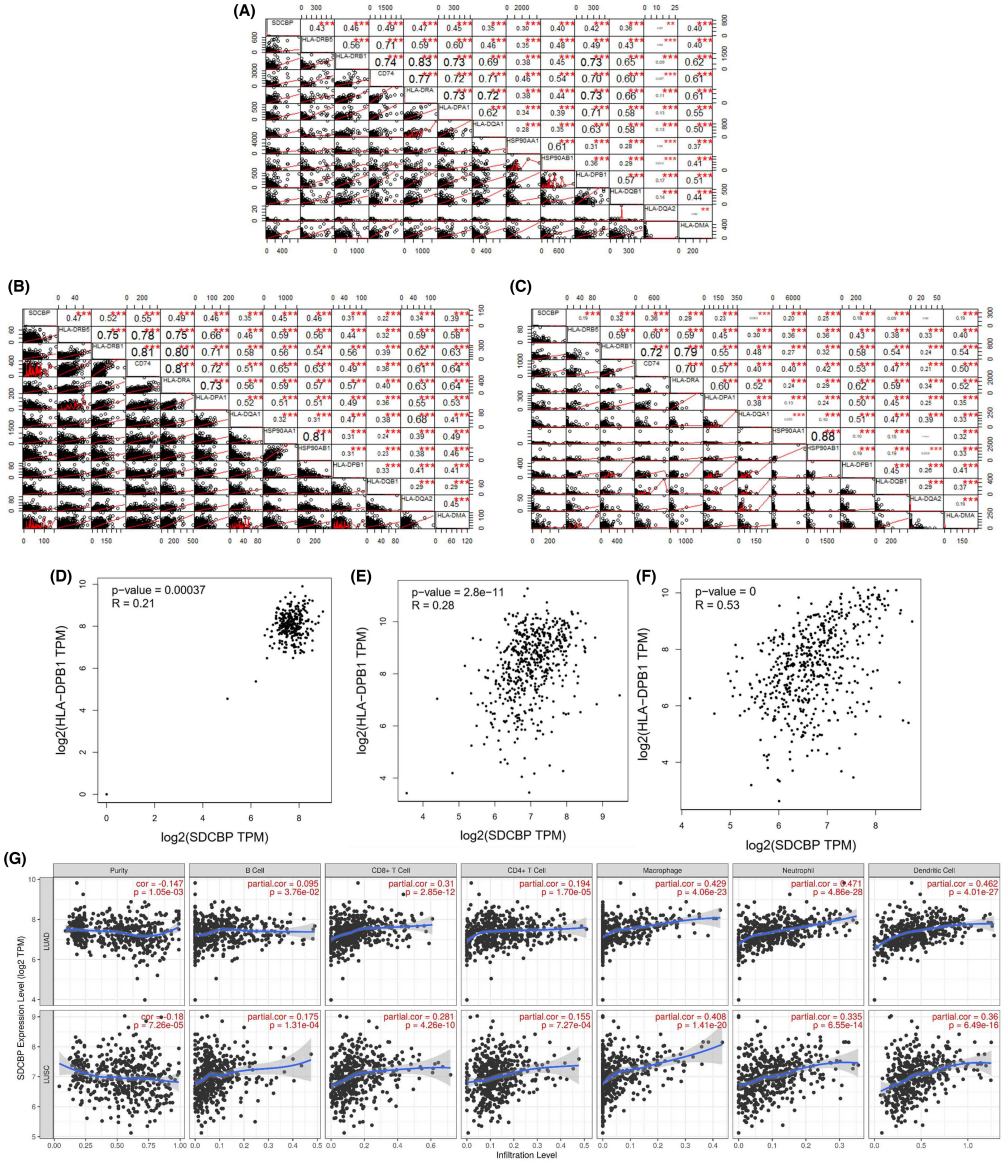
Relationship between SDCBP and the expression of antigen processing and presentation genes in BALF and lung tissue. (A–C) Correlation analysis (Pearson's correlation coefficients) between the expression of SDCBP and the expression of antigen processing and presentation genes based on GSE147143. Each circle in the lower left area represents a BALF cell, and the short red line represents the regression curve. Each datum in the upper right square represents the regression coefficient of the correlation relationship. (*p < 0.05, **p < 0.01, ***p < 0.001). (D) Correlation analysis between the expression of SDCBP and HLA‐DPB1 based on normal lung tissue data from GTEx. (E) Correlation analysis between the expression of SDCBP and HLA‐DPB1 based on the TCGA‐LUAD data. (F) Correlation analysis between the expression of SDCBP and HLA‐DPB1 based on the TCGA‐LUSC data. (G). Separate correlation analyses between the expression of SDCBP and B cells, CD8+ T cells, CD4+ T cells, macrophages, neutrophils and dendritic cells based on the TCGA‐LUAD and TCGA‐LUSC data.

The authors confirm all results and conclusions of this article remain unchanged. The authors wish to apologize for any misunderstanding or inconvenience caused.
