# Supplementary material for: Single‐cell RNA sequencing identify SDCBP in ACE2‐positive bronchial epithelial cells negatively correlates with COVID‐19 severity
Source: J Cell Mol Med. 2021 Jun 16;25(14):7001–12. doi: 10.1111/jcmm.16714 (PMC8278084; doi:10.1111/jcmm.16714)
Supplement: Supplementary file 1 — Figure S1 [file JCMM-25-7001-s009.docx]

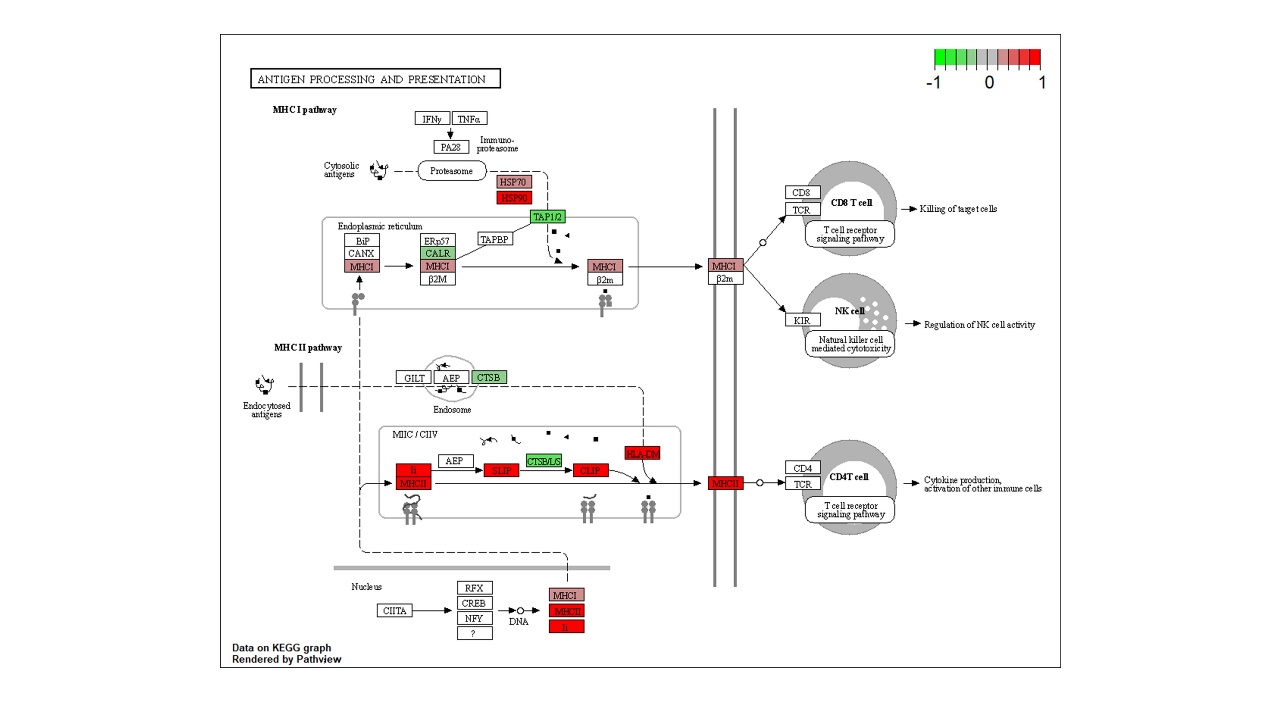


SUPPLEMENT FIGURE 1

Significant changes in antigen processing and presentation pathways between the mild and severe groups. View of the antigen processing and presentation pathway in KEGG.
