# Supplementary material for: Single‐cell RNA sequencing identify SDCBP in ACE2‐positive bronchial epithelial cells negatively correlates with COVID‐19 severity
Source: J Cell Mol Med. 2021 Jun 16;25(14):7001–12. doi: 10.1111/jcmm.16714 (PMC8278084; doi:10.1111/jcmm.16714)
Supplement: Supplementary file 2 — Figure S2 [file JCMM-25-7001-s002.docx]

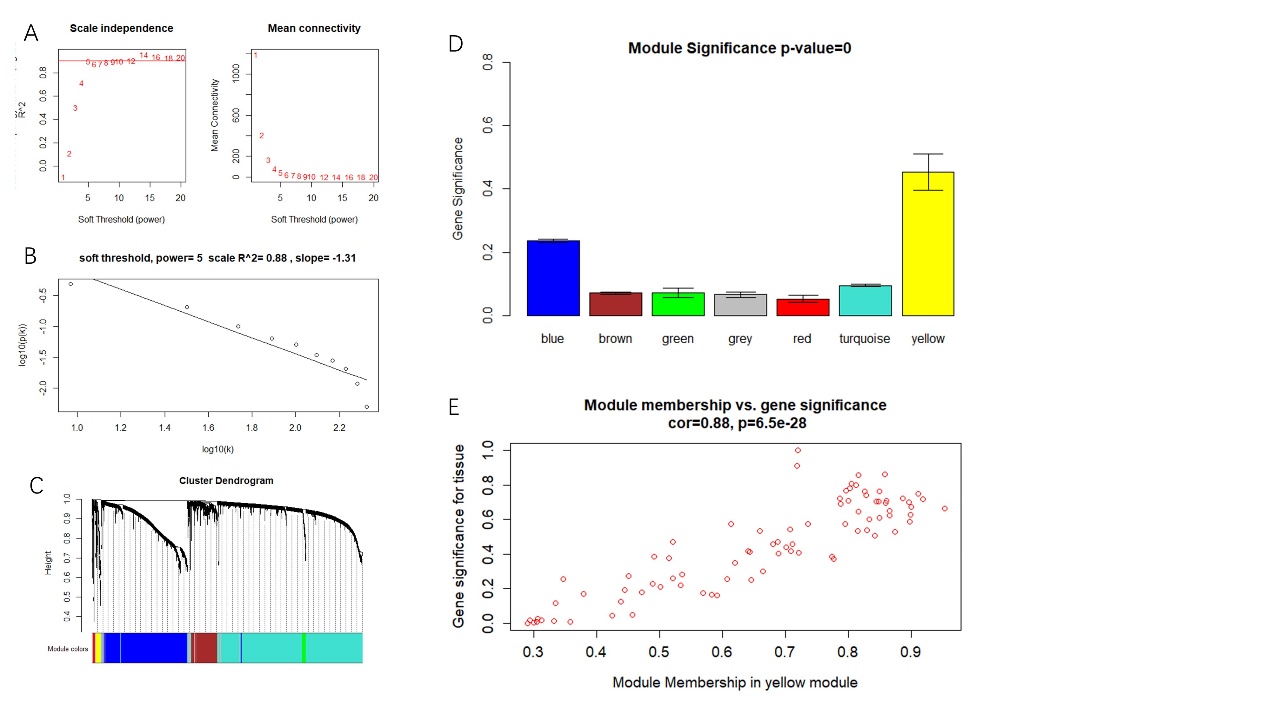


SUPPLEMENT FIGURE 2

Association between different modules and antigen processing and presentation genes. A. Left: Analysis of the scale‐free fit index for various soft‐thresholding powers (β). Right: Analysis of the mean connectivity for various soft‐thresholding powers. B. WGCNA, weighted gene coexpression network analysis. The scale‐free topology was checked when β = 5. C. Dendrogram of all differentially expressed genes clustered based on a dissimilarity measure (1‐TOM). D. Distribution of average gene significance and errors in the modules associated with the expression of HLA-DRB5. E. Module membership (MM) value and gene significance (GS) value of the yellow module.
