# Supplementary material for: Single‐cell RNA sequencing identify SDCBP in ACE2‐positive bronchial epithelial cells negatively correlates with COVID‐19 severity
Source: J Cell Mol Med. 2021 Jun 16;25(14):7001–12. doi: 10.1111/jcmm.16714 (PMC8278084; doi:10.1111/jcmm.16714)
Supplement: Supplementary file 3 — Figure S3 [file JCMM-25-7001-s004.docx]

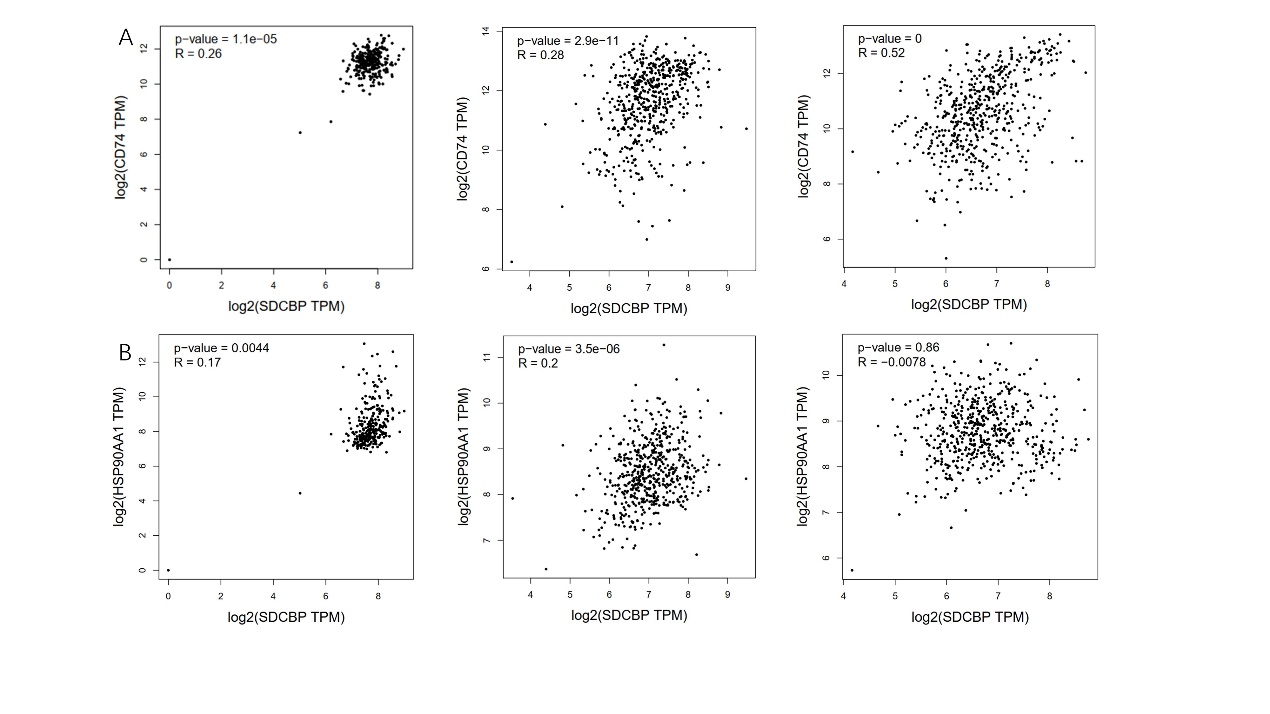


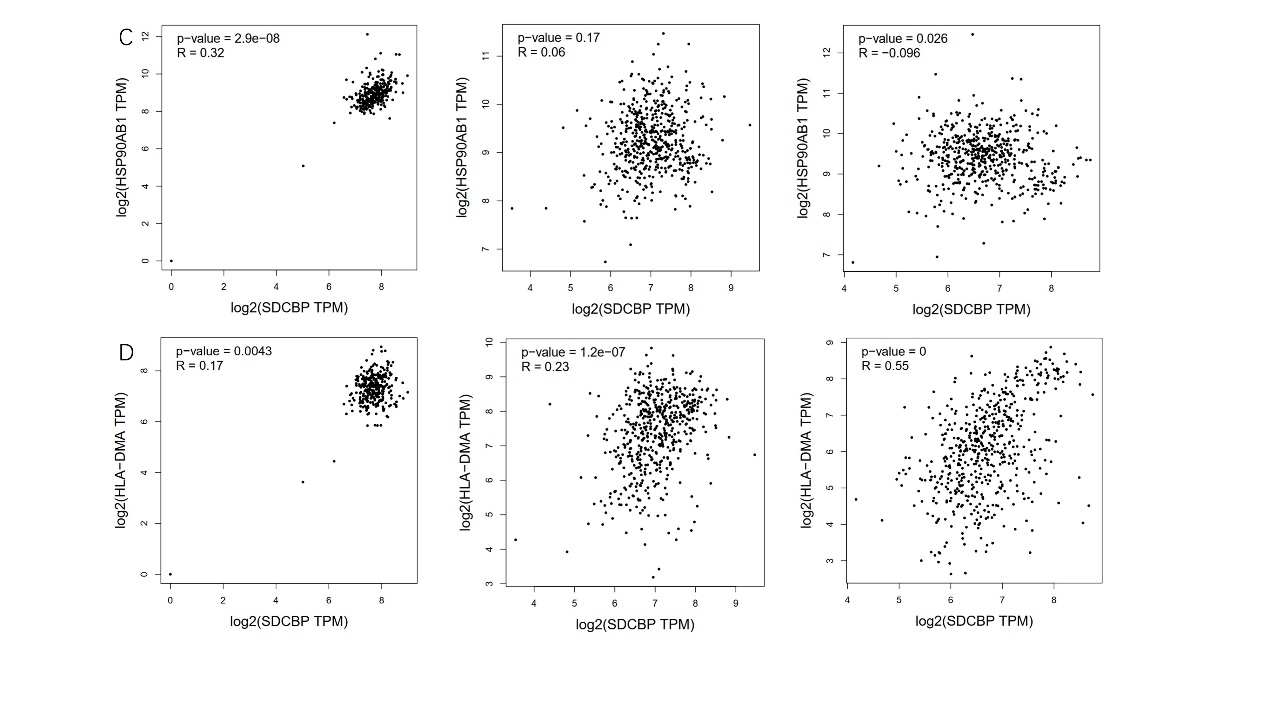


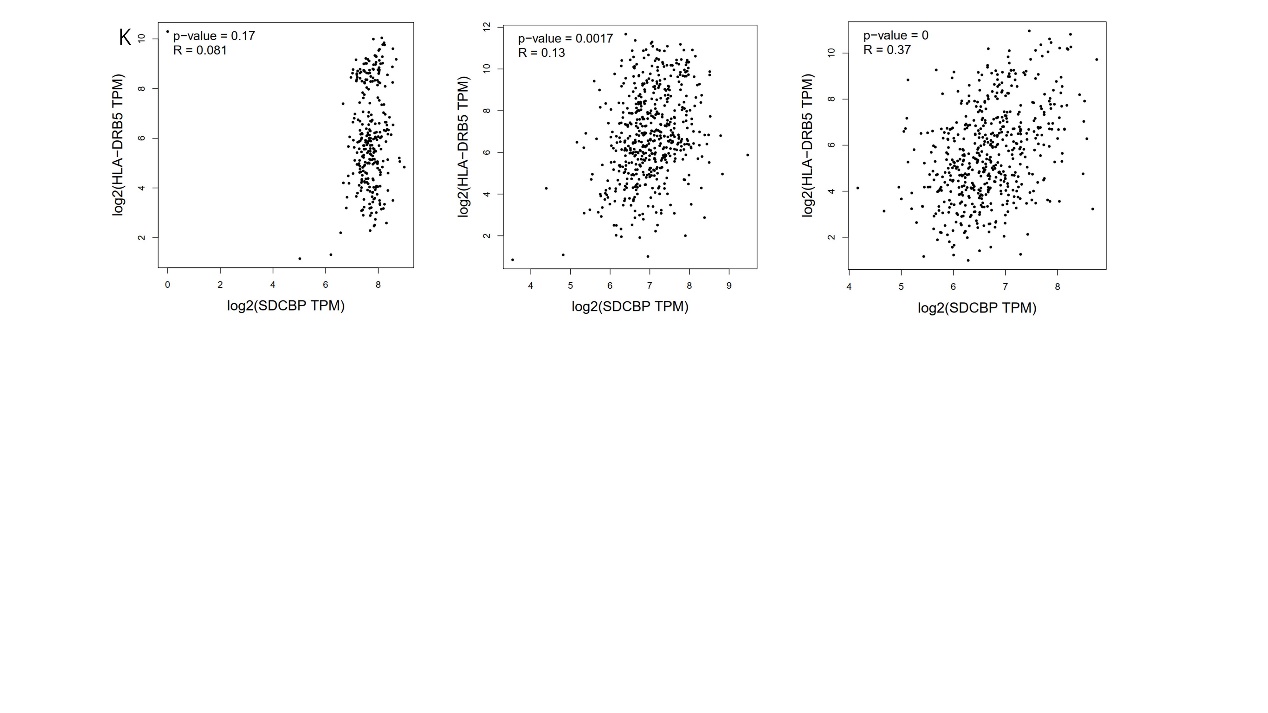


SUPPLEMENT FIGURE 3

Association between SDCBP and the following antigen processing and presentation genes: HLA-DRB5, HLA-DRB1, CD74, HLA-DRA, HLA-DPA1, HLA-DQA1, HSP90AA1, HSP90AB1, HLA-DQB1, HLA-DQA2, HLA-DMA. A. Correlation analysis between the expression of SDCBP and CD74. B. Correlation analysis between the expression of SDCBP and HSP90AA1. C. Correlation analysis between the expression of SDCBP and HSP90AB1. D. Correlation analysis between the expression of SDCBP and HLA-DMA. E. Correlation analysis between the expression of SDCBP and HLA-DPA1. F. Correlation analysis between the expression of SDCBP and HLA-DQA1. G. Correlation analysis between the expression of SDCBP and HLA-DQA2. H. Correlation analysis between the expression of SDCBP and HLA-DQB1. I. Correlation analysis between the expression of SDCBP and HLA-DRA. J. Correlation analysis between the expression of SDCBP and HLA-DRB1. K. Correlation analysis between the expression of SDCBP and HLA-DRB5. Left: Relationship of SDCBP with antigen processing and presentation genes in GTEx. Middle: Relationship of SDCBP with antigen processing and presentation genes in TCGA-LUAD. Right: Relationship of SDCBP with antigen processing and presentation genes in TCGA-LUSC.
