# Supplementary material for: Single‐cell RNA sequencing identify SDCBP in ACE2‐positive bronchial epithelial cells negatively correlates with COVID‐19 severity
Source: J Cell Mol Med. 2021 Jun 16;25(14):7001–12. doi: 10.1111/jcmm.16714 (PMC8278084; doi:10.1111/jcmm.16714)
Supplement: Supplementary file 5 — Table S2 [file JCMM-25-7001-s005.docx]

| gene | cell type |
| --- | --- |
| SLC34A2 | Epithelial cell |
| KL-6(MUC1) | Epithelial cell |
| ACTL6A | Epithelial cell |
| ACTN1 | Epithelial cell |
| AKT2 | Epithelial cell |
| AAK1 | Epithelial cell |
| CD83 | Dendritic cell |
| CD141(THBD) | Dendritic cell |
| NRP1 | Dendritic cell |
| CD11b(ITGAM) | Dendritic cell |
| CCL18 | macrophage |
| CD163 | macrophage |
| CD146(MCAM) | Endothelial cell |
| CD36 | Fibroblast |
| CD8A | T cell |
| GNLY | NK cell |
| CD19 | B cell |
