# Supplementary material for: Single‐cell RNA sequencing identify SDCBP in ACE2‐positive bronchial epithelial cells negatively correlates with COVID‐19 severity
Source: J Cell Mol Med. 2021 Jun 16;25(14):7001–12. doi: 10.1111/jcmm.16714 (PMC8278084; doi:10.1111/jcmm.16714)
Supplement: Supplementary file 6 — Table S3 [file JCMM-25-7001-s007.docx]

| gene | p_val | avg_logFC | pct.1 | pct.2 | p_val_adj |
| --- | --- | --- | --- | --- | --- |
| CNTD1 | 8.92E-24 | 0.367372 | 0.5 | 0 | 1.28E-19 |
| AC009502.1 | 6.61E-23 | 0.457362 | 0.588 | 0.021 | 9.50E-19 |
| AL109811.1 | 2.25E-22 | 0.510814 | 0.471 | 0 | 3.23E-18 |
| AC092802.1 | 2.25E-22 | 0.318899 | 0.471 | 0 | 3.23E-18 |
| RBM20 | 2.25E-22 | 0.293072 | 0.471 | 0 | 3.23E-18 |
| CCDC81 | 3.61E-22 | 0.485616 | 0.559 | 0.016 | 5.19E-18 |
| AC090371.1 | 1.81E-21 | 0.518775 | 0.559 | 0.021 | 2.60E-17 |
| PROS1 | 5.91E-21 | 0.918737 | 0.618 | 0.043 | 8.49E-17 |
| IL5RA | 2.23E-20 | 0.546581 | 0.647 | 0.053 | 3.20E-16 |
| C1orf189 | 6.20E-20 | 1.114041 | 0.618 | 0.048 | 8.91E-16 |
| SPATA4 | 9.04E-20 | 0.386697 | 0.588 | 0.037 | 1.30E-15 |
| PLA1A | 1.31E-19 | 0.289194 | 0.412 | 0 | 1.89E-15 |
| MYCBPAP | 1.38E-19 | 0.45989 | 0.588 | 0.037 | 1.98E-15 |
| AC096637.2 | 1.88E-19 | 0.314957 | 0.5 | 0.016 | 2.70E-15 |
| RSPH10B | 3.30E-19 | 0.300706 | 0.559 | 0.032 | 4.75E-15 |
| DCDC2B | 3.31E-19 | 0.73333 | 0.647 | 0.059 | 4.76E-15 |
| ST3GAL6 | 3.37E-19 | 0.5908 | 0.676 | 0.074 | 4.84E-15 |
| MRLN | 1.08E-18 | 0.388769 | 0.5 | 0.021 | 1.56E-14 |
| C11orf16 | 3.17E-18 | 0.755702 | 0.647 | 0.074 | 4.55E-14 |
| TTC16 | 3.87E-18 | 0.477139 | 0.559 | 0.037 | 5.56E-14 |
| AL590491.2 | 6.77E-18 | 0.478242 | 0.588 | 0.048 | 9.73E-14 |
| CFAP161 | 1.00E-17 | 0.37791 | 0.559 | 0.043 | 1.44E-13 |
| C2orf73 | 1.10E-17 | 0.516959 | 0.559 | 0.043 | 1.58E-13 |
| NHLRC4 | 1.11E-17 | 0.304395 | 0.529 | 0.032 | 1.60E-13 |
| FABP6 | 1.23E-17 | 0.727896 | 0.618 | 0.059 | 1.76E-13 |
| GABPB1-AS1 | 1.27E-17 | 0.589076 | 0.824 | 0.16 | 1.83E-13 |
| TXLNB | 1.31E-17 | 0.389492 | 0.559 | 0.043 | 1.88E-13 |
| SLC23A1 | 1.74E-17 | 0.338738 | 0.588 | 0.048 | 2.51E-13 |
| ECT2L | 2.09E-17 | 0.62011 | 0.618 | 0.064 | 3.00E-13 |
| USP2-AS1 | 2.91E-17 | 0.268539 | 0.5 | 0.027 | 4.18E-13 |
| FBXO15 | 3.42E-17 | 0.581125 | 0.559 | 0.048 | 4.92E-13 |
| JHY | 3.51E-17 | 0.316914 | 0.647 | 0.069 | 5.04E-13 |
| AC009652.2 | 3.97E-17 | 0.481571 | 0.588 | 0.059 | 5.70E-13 |
| CFAP58 | 4.10E-17 | 0.322713 | 0.559 | 0.043 | 5.90E-13 |
| KCNRG | 5.70E-17 | 0.326888 | 0.529 | 0.037 | 8.19E-13 |
| RIIAD1 | 7.48E-17 | 0.600983 | 0.647 | 0.074 | 1.08E-12 |
| AK7 | 1.32E-16 | 0.681251 | 0.676 | 0.096 | 1.89E-12 |
| WFDC6 | 1.40E-16 | 0.48005 | 0.588 | 0.059 | 2.01E-12 |
| CASC2 | 1.65E-16 | 0.432623 | 0.647 | 0.08 | 2.37E-12 |
| LRRC34 | 1.98E-16 | 0.458649 | 0.618 | 0.069 | 2.85E-12 |
| CDC14A | 2.00E-16 | 0.269378 | 0.706 | 0.096 | 2.87E-12 |
| CATSPERD | 2.71E-16 | 0.703887 | 0.618 | 0.074 | 3.89E-12 |
| C10orf67 | 3.80E-16 | 0.417001 | 0.529 | 0.043 | 5.46E-12 |
| TACSTD2 | 4.14E-16 | -1.71632 | 0.971 | 0.968 | 5.95E-12 |
| CFAP299 | 4.95E-16 | 0.509782 | 0.618 | 0.074 | 7.12E-12 |
| GAS2L2 | 5.20E-16 | 0.451133 | 0.647 | 0.085 | 7.47E-12 |
| S100A10 | 5.64E-16 | -1.92335 | 0.765 | 0.989 | 8.10E-12 |
| UCKL1-AS1 | 5.80E-16 | 0.29935 | 0.559 | 0.053 | 8.33E-12 |
| ARMC4 | 7.59E-16 | 0.726056 | 0.647 | 0.09 | 1.09E-11 |
| AC078864.2 | 1.02E-15 | 0.523396 | 0.559 | 0.053 | 1.47E-11 |
| CCDC39 | 1.09E-15 | 0.444471 | 0.618 | 0.08 | 1.57E-11 |
| MAP3K19 | 1.59E-15 | 0.86697 | 0.647 | 0.096 | 2.29E-11 |
| MDM1 | 1.85E-15 | 0.318714 | 0.647 | 0.09 | 2.65E-11 |
| CCDC180 | 2.27E-15 | 0.593779 | 0.618 | 0.08 | 3.27E-11 |
| TTC26 | 2.63E-15 | 0.436395 | 0.706 | 0.122 | 3.78E-11 |
| POU2AF1 | 2.72E-15 | 0.593212 | 0.676 | 0.112 | 3.91E-11 |
| LRRC74B | 2.74E-15 | 0.64817 | 0.588 | 0.08 | 3.94E-11 |
| TTLL9 | 2.94E-15 | 0.356267 | 0.588 | 0.069 | 4.23E-11 |
| LINC01571 | 3.38E-15 | 0.518193 | 0.559 | 0.059 | 4.85E-11 |
| CROCC2 | 4.28E-15 | 0.501437 | 0.588 | 0.069 | 6.15E-11 |
| TEKT4 | 4.61E-15 | 0.461857 | 0.588 | 0.069 | 6.62E-11 |
| KIAA2012 | 4.73E-15 | 0.512495 | 0.618 | 0.085 | 6.79E-11 |
| LRRC43 | 6.05E-15 | 0.285791 | 0.559 | 0.059 | 8.70E-11 |
| DNAJB13 | 6.17E-15 | 0.58304 | 0.647 | 0.101 | 8.86E-11 |
| KLHL6 | 6.97E-15 | 0.540008 | 0.647 | 0.09 | 1.00E-10 |
| CLDN4 | 7.62E-15 | -1.81356 | 0.912 | 0.952 | 1.10E-10 |
| LRRIQ3 | 1.03E-14 | 0.265282 | 0.441 | 0.027 | 1.49E-10 |
| OSBPL6 | 1.47E-14 | 0.750337 | 0.618 | 0.101 | 2.11E-10 |
| TM4SF1 | 1.82E-14 | -2.02632 | 0.088 | 0.835 | 2.62E-10 |
| LAMB3 | 1.84E-14 | -1.11023 | 0 | 0.814 | 2.65E-10 |
| IGFBP5 | 1.93E-14 | 0.806852 | 0.794 | 0.207 | 2.78E-10 |
| DZIP1L | 1.99E-14 | 0.765674 | 0.618 | 0.101 | 2.86E-10 |
| AC125611.4 | 2.30E-14 | 0.260445 | 0.529 | 0.059 | 3.30E-10 |
| ZMYND12 | 2.46E-14 | 0.32974 | 0.559 | 0.069 | 3.53E-10 |
| STK33 | 2.51E-14 | 0.903951 | 0.647 | 0.128 | 3.61E-10 |
| ADGB | 2.57E-14 | 0.524869 | 0.618 | 0.09 | 3.70E-10 |
| RBM41 | 2.65E-14 | 0.297129 | 0.559 | 0.064 | 3.80E-10 |
| EFHB | 2.80E-14 | 0.720742 | 0.647 | 0.112 | 4.02E-10 |
| CERKL | 3.73E-14 | 0.378454 | 0.618 | 0.085 | 5.35E-10 |
| TCTE1 | 3.79E-14 | 0.274981 | 0.471 | 0.037 | 5.45E-10 |
| PIH1D3 | 4.84E-14 | 0.539969 | 0.588 | 0.08 | 6.95E-10 |
| STOML3 | 5.07E-14 | 0.778469 | 0.647 | 0.106 | 7.28E-10 |
| CD74 | 6.16E-14 | 1.024809 | 1 | 0.989 | 8.85E-10 |
| AC113349.1 | 6.25E-14 | 0.497745 | 0.588 | 0.085 | 8.98E-10 |
| DIAPH2 | 6.54E-14 | 0.445286 | 0.676 | 0.128 | 9.39E-10 |
| DNAH2 | 9.22E-14 | 0.617505 | 0.647 | 0.112 | 1.33E-09 |
| SDCBP2 | 9.34E-14 | -1.19013 | 0.118 | 0.83 | 1.34E-09 |
| LINC02345 | 1.02E-13 | 0.324027 | 0.559 | 0.069 | 1.46E-09 |
| TXN | 1.12E-13 | -1.2228 | 1 | 0.984 | 1.60E-09 |
| KLF6 | 1.31E-13 | -1.39842 | 0.765 | 0.947 | 1.88E-09 |
| ANKUB1 | 1.41E-13 | 0.744176 | 0.647 | 0.133 | 2.03E-09 |
| CASC1 | 1.69E-13 | 0.513219 | 0.588 | 0.085 | 2.42E-09 |
| EFCAB10 | 1.71E-13 | 0.64985 | 0.618 | 0.101 | 2.46E-09 |
| FXYD1 | 1.89E-13 | 0.39442 | 0.529 | 0.064 | 2.72E-09 |
| CFAP52 | 1.91E-13 | 0.652947 | 0.647 | 0.117 | 2.75E-09 |
| MORN5 | 1.91E-13 | 1.037057 | 0.647 | 0.128 | 2.75E-09 |
| SLC13A3 | 1.94E-13 | 0.46636 | 0.559 | 0.08 | 2.79E-09 |
| MT-ATP8 | 1.97E-13 | 1.632638 | 1 | 0.957 | 2.83E-09 |
| RIPOR2 | 2.15E-13 | 0.619352 | 0.706 | 0.149 | 3.09E-09 |
| FANCF | 2.25E-13 | 0.305611 | 0.765 | 0.17 | 3.24E-09 |
| ANXA1 | 2.34E-13 | -1.41028 | 0.941 | 0.989 | 3.36E-09 |
| ZNF474 | 2.54E-13 | 0.370664 | 0.588 | 0.08 | 3.65E-09 |
| ERICH6-AS1 | 2.58E-13 | 0.414435 | 0.5 | 0.059 | 3.71E-09 |
| DHRS4-AS1 | 2.63E-13 | 0.308375 | 0.559 | 0.085 | 3.78E-09 |
| ACTG1 | 2.67E-13 | -1.33226 | 1 | 0.989 | 3.83E-09 |
| CATIP | 2.71E-13 | 0.466191 | 0.588 | 0.085 | 3.89E-09 |
| WDR93 | 2.75E-13 | 0.31775 | 0.5 | 0.053 | 3.96E-09 |
| C11orf97 | 2.84E-13 | 0.948657 | 0.647 | 0.128 | 4.09E-09 |
| ANKFN1 | 3.13E-13 | 0.361119 | 0.588 | 0.09 | 4.50E-09 |
| PHLDA2 | 3.15E-13 | -1.39919 | 0.235 | 0.84 | 4.53E-09 |
| KRT7 | 3.82E-13 | -1.95326 | 0.647 | 0.92 | 5.49E-09 |
| FILIP1 | 3.97E-13 | 0.54454 | 0.647 | 0.128 | 5.71E-09 |
| CAPN2 | 4.07E-13 | -0.909 | 0.882 | 0.947 | 5.85E-09 |
| KIF6 | 4.21E-13 | 0.291648 | 0.529 | 0.069 | 6.05E-09 |
| DCDC1 | 4.28E-13 | 0.497921 | 0.647 | 0.117 | 6.14E-09 |
| SFN | 4.41E-13 | -1.88932 | 0.088 | 0.824 | 6.34E-09 |
| PPP1R42 | 4.67E-13 | 0.450371 | 0.618 | 0.106 | 6.71E-09 |
| TEKT3 | 5.04E-13 | 0.269908 | 0.471 | 0.048 | 7.25E-09 |
| AC084033.3 | 5.46E-13 | 0.842085 | 0.676 | 0.16 | 7.84E-09 |
| PLTP | 5.51E-13 | 0.401526 | 0.412 | 0.032 | 7.91E-09 |
| EPPIN | 5.64E-13 | 0.610432 | 0.618 | 0.106 | 8.11E-09 |
| GPRC5A | 6.15E-13 | -1.98339 | 0.294 | 0.851 | 8.84E-09 |
| LCA5L | 6.17E-13 | 0.390867 | 0.618 | 0.101 | 8.87E-09 |
| APOBEC4 | 6.81E-13 | 0.554901 | 0.618 | 0.106 | 9.79E-09 |
| C1orf158 | 7.48E-13 | 0.48103 | 0.618 | 0.106 | 1.08E-08 |
| ZFP36 | 7.69E-13 | -1.29316 | 0.647 | 0.915 | 1.10E-08 |
| CCDC191 | 7.73E-13 | 0.499987 | 0.676 | 0.138 | 1.11E-08 |
| DRC7 | 7.97E-13 | 0.513644 | 0.618 | 0.106 | 1.14E-08 |
| TEKT1 | 8.58E-13 | 1.013434 | 0.647 | 0.133 | 1.23E-08 |
| DNAH10 | 1.00E-12 | 0.816251 | 0.647 | 0.144 | 1.44E-08 |
| CFAP61 | 1.02E-12 | 0.355269 | 0.5 | 0.059 | 1.46E-08 |
| SPAG8 | 1.06E-12 | 0.731338 | 0.647 | 0.133 | 1.52E-08 |
| C16orf71 | 1.09E-12 | 0.393201 | 0.529 | 0.074 | 1.57E-08 |
| CCDC30 | 1.12E-12 | 0.666862 | 0.647 | 0.133 | 1.61E-08 |
| AC013264.1 | 1.25E-12 | 1.061114 | 0.618 | 0.128 | 1.79E-08 |
| ARMH4 | 1.27E-12 | 0.38855 | 0.559 | 0.085 | 1.83E-08 |
| RGS22 | 1.31E-12 | 0.381427 | 0.559 | 0.08 | 1.88E-08 |
| KRT17 | 1.39E-12 | -3.3878 | 0 | 0.745 | 2.00E-08 |
| KPNA5 | 1.44E-12 | 0.357293 | 0.647 | 0.128 | 2.07E-08 |
| HYDIN | 1.48E-12 | 0.919096 | 0.706 | 0.176 | 2.12E-08 |
| DNAI2 | 1.51E-12 | 0.845227 | 0.647 | 0.138 | 2.17E-08 |
| DTHD1 | 1.60E-12 | 0.949461 | 0.647 | 0.144 | 2.29E-08 |
| AC007114.1 | 1.62E-12 | 0.274117 | 0.529 | 0.08 | 2.33E-08 |
| RSPH14 | 1.77E-12 | 0.517127 | 0.559 | 0.085 | 2.55E-08 |
| CCDC151 | 1.81E-12 | 0.352309 | 0.471 | 0.053 | 2.60E-08 |
| IL1RN | 1.91E-12 | -1.25141 | 0 | 0.739 | 2.74E-08 |
| KCNE1 | 2.03E-12 | 0.434005 | 0.588 | 0.101 | 2.92E-08 |
| MIA | 2.06E-12 | 0.362791 | 0.441 | 0.048 | 2.96E-08 |
| CD99L2 | 2.22E-12 | 0.395685 | 0.706 | 0.176 | 3.19E-08 |
| SERPINI2 | 2.23E-12 | 0.328298 | 0.559 | 0.085 | 3.20E-08 |
| TTC12 | 2.26E-12 | 0.41529 | 0.618 | 0.128 | 3.25E-08 |
| SLC22A4 | 2.47E-12 | 0.681537 | 0.647 | 0.138 | 3.55E-08 |
| TSPAN19 | 2.56E-12 | 0.924689 | 0.676 | 0.165 | 3.68E-08 |
| S100A2 | 2.58E-12 | -3.35421 | 0.265 | 0.819 | 3.70E-08 |
| MDH1B | 2.59E-12 | 0.456858 | 0.647 | 0.128 | 3.72E-08 |
| JUN | 2.67E-12 | -1.51389 | 0.706 | 0.915 | 3.84E-08 |
| DZANK1 | 2.75E-12 | 0.301513 | 0.529 | 0.074 | 3.95E-08 |
| CFAP65 | 2.91E-12 | 0.601526 | 0.647 | 0.128 | 4.18E-08 |
| FHAD1 | 3.04E-12 | 0.876671 | 0.676 | 0.176 | 4.37E-08 |
| AC008771.1 | 3.18E-12 | 0.386059 | 0.676 | 0.154 | 4.57E-08 |
| TCTN2 | 3.21E-12 | 0.270858 | 0.559 | 0.08 | 4.61E-08 |
| FOSB | 3.27E-12 | -1.66635 | 0.088 | 0.761 | 4.70E-08 |
| AKAP14 | 3.39E-12 | 0.71219 | 0.588 | 0.106 | 4.87E-08 |
| KATNAL2 | 3.43E-12 | 0.253101 | 0.5 | 0.064 | 4.93E-08 |
| ERICH3 | 3.51E-12 | 1.175765 | 0.647 | 0.154 | 5.04E-08 |
| FGFBP1 | 3.55E-12 | -1.81223 | 0 | 0.729 | 5.10E-08 |
| IQUB | 3.68E-12 | 0.598843 | 0.559 | 0.096 | 5.29E-08 |
| ANKRD66 | 3.95E-12 | 0.69445 | 0.588 | 0.106 | 5.68E-08 |
| PKIG | 4.20E-12 | 0.558232 | 0.765 | 0.223 | 6.04E-08 |
| EMP1 | 4.22E-12 | -1.54244 | 0.088 | 0.777 | 6.06E-08 |
| HLA-DRB5 | 4.31E-12 | 1.429062 | 0.941 | 0.622 | 6.19E-08 |
| DYNC2H1 | 4.32E-12 | 0.636799 | 0.676 | 0.16 | 6.21E-08 |
| EFCAB6 | 4.75E-12 | 0.305542 | 0.588 | 0.096 | 6.82E-08 |
| BAIAP3 | 4.99E-12 | 0.426423 | 0.647 | 0.122 | 7.17E-08 |
| C6orf118 | 5.02E-12 | 0.768262 | 0.647 | 0.16 | 7.21E-08 |
| LRRC8A | 5.16E-12 | -1.10947 | 0.147 | 0.787 | 7.42E-08 |
| LINC02541 | 5.36E-12 | 0.29876 | 0.559 | 0.085 | 7.70E-08 |
| TCTEX1D4 | 6.26E-12 | 0.940917 | 0.618 | 0.133 | 9.00E-08 |
| CFAP57 | 6.29E-12 | 0.56835 | 0.618 | 0.128 | 9.04E-08 |
| C20orf96 | 6.41E-12 | 0.645214 | 0.706 | 0.191 | 9.21E-08 |
| AC007405.3 | 6.53E-12 | 0.461274 | 0.559 | 0.09 | 9.39E-08 |
| CCDC13 | 6.58E-12 | 0.253468 | 0.471 | 0.053 | 9.45E-08 |
| NEK10 | 6.69E-12 | 0.592098 | 0.647 | 0.138 | 9.62E-08 |
| ZBED5-AS1 | 7.27E-12 | 0.411052 | 0.706 | 0.181 | 1.04E-07 |
| KRT8 | 7.90E-12 | -1.02635 | 0.971 | 0.968 | 1.14E-07 |
| DNAH5 | 8.69E-12 | 1.465385 | 0.676 | 0.202 | 1.25E-07 |
| IER2 | 9.10E-12 | -1.27863 | 0.618 | 0.899 | 1.31E-07 |
| TNNT3 | 9.18E-12 | 0.256054 | 0.529 | 0.074 | 1.32E-07 |
| C19orf33 | 9.75E-12 | -1.00736 | 0.853 | 0.931 | 1.40E-07 |
| CCDC74B | 1.04E-11 | 0.534227 | 0.588 | 0.106 | 1.50E-07 |
| HLA-DQA2 | 1.08E-11 | 0.672813 | 0.441 | 0.053 | 1.55E-07 |
| VIM-AS1 | 1.12E-11 | 0.332375 | 0.471 | 0.059 | 1.61E-07 |
| MAP1A | 1.15E-11 | 0.705613 | 0.647 | 0.144 | 1.66E-07 |
| MORN1 | 1.18E-11 | 0.300605 | 0.529 | 0.08 | 1.70E-07 |
| C22orf15 | 1.19E-11 | 0.784946 | 0.618 | 0.138 | 1.70E-07 |
| BEST4 | 1.19E-11 | 0.57641 | 0.5 | 0.074 | 1.71E-07 |
| ZYX | 1.19E-11 | -0.73505 | 0 | 0.707 | 1.72E-07 |
| NME5 | 1.24E-11 | 0.837237 | 0.735 | 0.229 | 1.78E-07 |
| TMSB10 | 1.28E-11 | -0.88979 | 1 | 0.995 | 1.84E-07 |
| RP1 | 1.30E-11 | 0.847292 | 0.647 | 0.16 | 1.87E-07 |
| CCDC33 | 1.37E-11 | 0.832117 | 0.618 | 0.144 | 1.96E-07 |
| CFAP69 | 1.39E-11 | 0.475745 | 0.676 | 0.16 | 1.99E-07 |
| DNAH12 | 1.52E-11 | 1.425708 | 0.676 | 0.207 | 2.19E-07 |
| TCTEX1D1 | 1.58E-11 | 0.516064 | 0.618 | 0.128 | 2.27E-07 |
| DAW1 | 1.70E-11 | 0.424105 | 0.559 | 0.101 | 2.45E-07 |
| CABCOCO1 | 1.76E-11 | 0.572095 | 0.588 | 0.112 | 2.53E-07 |
| ARMC2 | 1.85E-11 | 0.499124 | 0.588 | 0.128 | 2.66E-07 |
| FAM166B | 1.89E-11 | 0.888664 | 0.647 | 0.149 | 2.72E-07 |
| FAM227A | 1.98E-11 | 0.554159 | 0.618 | 0.128 | 2.84E-07 |
| LRRC10B | 1.98E-11 | 0.671968 | 0.588 | 0.112 | 2.85E-07 |
| PROM1 | 2.00E-11 | 0.950548 | 0.735 | 0.255 | 2.87E-07 |
| FAM27C | 2.02E-11 | 0.350901 | 0.559 | 0.09 | 2.90E-07 |
| MORN3 | 2.05E-11 | 0.436655 | 0.588 | 0.117 | 2.95E-07 |
| CFAP221 | 2.11E-11 | 0.766845 | 0.618 | 0.149 | 3.03E-07 |
| AL121899.1 | 2.19E-11 | 0.417686 | 0.529 | 0.085 | 3.14E-07 |
| TSGA10 | 2.19E-11 | 0.534991 | 0.735 | 0.245 | 3.15E-07 |
| BCAS3 | 2.31E-11 | 0.299552 | 0.559 | 0.101 | 3.31E-07 |
| ABCA13 | 2.34E-11 | 0.949371 | 0.912 | 0.654 | 3.36E-07 |
| SLC7A2 | 2.40E-11 | 0.68982 | 0.676 | 0.202 | 3.45E-07 |
| HLA-DRB1 | 2.67E-11 | 1.06102 | 0.971 | 0.862 | 3.84E-07 |
| TMEM232 | 2.81E-11 | 0.559588 | 0.618 | 0.138 | 4.04E-07 |
| FBXW9 | 2.84E-11 | 0.344588 | 0.647 | 0.149 | 4.08E-07 |
| VWA3A | 2.84E-11 | 0.814765 | 0.647 | 0.165 | 4.09E-07 |
| TTC21B | 2.85E-11 | 0.324859 | 0.559 | 0.101 | 4.09E-07 |
| DNAH11 | 2.88E-11 | 0.754213 | 0.647 | 0.154 | 4.14E-07 |
| CFAP74 | 2.90E-11 | 0.499626 | 0.588 | 0.112 | 4.17E-07 |
| NCALD | 2.92E-11 | 0.446275 | 0.588 | 0.138 | 4.20E-07 |
| FRMPD2 | 2.97E-11 | 0.487488 | 0.588 | 0.122 | 4.26E-07 |
| TNFRSF12A | 3.04E-11 | -1.20002 | 0.206 | 0.782 | 4.36E-07 |
| EFCAB1 | 3.08E-11 | 1.107674 | 0.647 | 0.181 | 4.42E-07 |
| TIMP4 | 3.08E-11 | 0.274044 | 0.441 | 0.053 | 4.43E-07 |
| C15orf48 | 3.13E-11 | -1.88249 | 0.118 | 0.739 | 4.49E-07 |
| FAM216B | 3.14E-11 | 0.985 | 0.618 | 0.138 | 4.52E-07 |
| OMG | 3.18E-11 | 1.004089 | 0.647 | 0.144 | 4.56E-07 |
| CDHR4 | 3.21E-11 | 0.746033 | 0.647 | 0.154 | 4.61E-07 |
| SPEF1 | 3.23E-11 | 0.476205 | 0.647 | 0.133 | 4.64E-07 |
| DNAI1 | 3.25E-11 | 0.651357 | 0.647 | 0.149 | 4.66E-07 |
| EFHC2 | 3.32E-11 | 0.373266 | 0.588 | 0.106 | 4.77E-07 |
| HMGN5 | 3.41E-11 | 0.375697 | 0.618 | 0.138 | 4.91E-07 |
| TTC25 | 3.44E-11 | 0.699201 | 0.618 | 0.149 | 4.95E-07 |
| ELN-AS1 | 3.49E-11 | 0.437299 | 0.618 | 0.144 | 5.01E-07 |
| HAGHL | 3.70E-11 | 0.384975 | 0.618 | 0.133 | 5.32E-07 |
| TOGARAM2 | 3.88E-11 | 0.588735 | 0.618 | 0.128 | 5.58E-07 |
| WDR54 | 4.02E-11 | 0.802013 | 0.735 | 0.234 | 5.78E-07 |
| TMEM241 | 4.04E-11 | 0.376102 | 0.5 | 0.08 | 5.80E-07 |
| MAL2 | 4.14E-11 | -0.94344 | 0.824 | 0.926 | 5.95E-07 |
| ENKD1 | 4.19E-11 | 0.377759 | 0.676 | 0.144 | 6.02E-07 |
| CCDC113 | 4.21E-11 | 0.874255 | 0.735 | 0.223 | 6.05E-07 |
| PTPRT | 4.51E-11 | 0.452614 | 0.618 | 0.149 | 6.48E-07 |
| NEK5 | 4.52E-11 | 0.784918 | 0.647 | 0.181 | 6.50E-07 |
| S100A6 | 4.83E-11 | -0.847 | 1 | 1 | 6.93E-07 |
| HIST1H4C | 4.91E-11 | 0.723338 | 0.941 | 0.612 | 7.06E-07 |
| CCDC69 | 4.92E-11 | 0.471588 | 0.853 | 0.351 | 7.06E-07 |
| LINC01765 | 5.53E-11 | 0.631511 | 0.618 | 0.144 | 7.95E-07 |
| EFCAB12 | 5.61E-11 | 0.344691 | 0.588 | 0.117 | 8.07E-07 |
| CCDC125 | 5.95E-11 | 0.496221 | 0.853 | 0.362 | 8.55E-07 |
| NWD1 | 6.35E-11 | 0.960791 | 0.676 | 0.213 | 9.12E-07 |
| FUZ | 6.45E-11 | 0.484729 | 0.706 | 0.223 | 9.27E-07 |
| SAXO2 | 6.50E-11 | 1.048227 | 0.676 | 0.213 | 9.35E-07 |
| ABCC6 | 6.66E-11 | 0.346006 | 0.588 | 0.117 | 9.57E-07 |
| ATP2C2 | 6.75E-11 | 0.572799 | 0.941 | 0.457 | 9.70E-07 |
| C2orf40 | 6.76E-11 | 1.39604 | 0.647 | 0.176 | 9.71E-07 |
| CFAP70 | 6.93E-11 | 0.777515 | 0.676 | 0.207 | 9.96E-07 |
| CXCL8 | 7.08E-11 | -1.9703 | 0.118 | 0.745 | 1.02E-06 |
| AGBL2 | 7.10E-11 | 0.43567 | 0.647 | 0.154 | 1.02E-06 |
| TNFAIP8L1 | 7.12E-11 | 0.669696 | 0.647 | 0.16 | 1.02E-06 |
| ZNF606 | 7.39E-11 | 0.250285 | 0.5 | 0.08 | 1.06E-06 |
| ZC2HC1C | 7.88E-11 | 0.294358 | 0.5 | 0.08 | 1.13E-06 |
| TMEM107 | 7.88E-11 | 0.351253 | 0.676 | 0.17 | 1.13E-06 |
| FOS | 7.94E-11 | -1.78275 | 0.324 | 0.798 | 1.14E-06 |
| CEACAM5 | 7.96E-11 | -1.81435 | 0.118 | 0.723 | 1.14E-06 |
| TPT1 | 7.99E-11 | -0.69821 | 1 | 1 | 1.15E-06 |
| LRRC71 | 8.34E-11 | 0.442034 | 0.647 | 0.144 | 1.20E-06 |
| IGFBP3 | 8.84E-11 | -1.55194 | 0.176 | 0.755 | 1.27E-06 |
| STOX1 | 8.95E-11 | 0.430828 | 0.676 | 0.17 | 1.29E-06 |
| SYTL3 | 9.04E-11 | 0.409033 | 0.618 | 0.149 | 1.30E-06 |
| DNAAF3 | 9.08E-11 | 0.502087 | 0.588 | 0.117 | 1.31E-06 |
| SRGAP3 | 9.09E-11 | 0.45158 | 0.794 | 0.287 | 1.31E-06 |
| F3 | 9.25E-11 | -1.51928 | 0.588 | 0.888 | 1.33E-06 |
| RPL39 | 9.32E-11 | -0.77261 | 0.941 | 0.984 | 1.34E-06 |
| WDR38 | 9.41E-11 | 1.068976 | 0.647 | 0.176 | 1.35E-06 |
| DNAH9 | 9.45E-11 | 0.935722 | 0.647 | 0.181 | 1.36E-06 |
| TTLL10 | 9.47E-11 | 0.618444 | 0.647 | 0.16 | 1.36E-06 |
| FBXO36 | 9.60E-11 | 0.374714 | 0.618 | 0.138 | 1.38E-06 |
| ACTB | 9.87E-11 | -1.14793 | 1 | 0.995 | 1.42E-06 |
| CD9 | 1.01E-10 | -0.8762 | 1 | 0.957 | 1.45E-06 |
| AK9 | 1.01E-10 | 0.563451 | 0.706 | 0.202 | 1.45E-06 |
| AC013470.2 | 1.01E-10 | 0.387916 | 0.5 | 0.08 | 1.46E-06 |
| CFAP53 | 1.03E-10 | 1.110282 | 0.676 | 0.213 | 1.48E-06 |
| ALS2CR12 | 1.04E-10 | 0.292129 | 0.471 | 0.064 | 1.49E-06 |
| DRC3 | 1.04E-10 | 1.144814 | 0.647 | 0.207 | 1.49E-06 |
| TTC30B | 1.11E-10 | 0.313954 | 0.618 | 0.128 | 1.59E-06 |
| TMEM212 | 1.13E-10 | 0.41324 | 0.412 | 0.048 | 1.62E-06 |
| TRIP13 | 1.13E-10 | 0.34072 | 0.529 | 0.101 | 1.63E-06 |
| IK | 1.17E-10 | 1.229683 | 0.941 | 0.718 | 1.69E-06 |
| MRPS31 | 1.19E-10 | 1.024743 | 0.853 | 0.42 | 1.71E-06 |
| PEAK1 | 1.21E-10 | 0.291842 | 0.765 | 0.234 | 1.74E-06 |
| SMPD3 | 1.23E-10 | 0.419217 | 0.647 | 0.16 | 1.76E-06 |
| NME9 | 1.26E-10 | 0.317552 | 0.5 | 0.08 | 1.81E-06 |
| AK8 | 1.27E-10 | 0.361184 | 0.529 | 0.096 | 1.82E-06 |
| CAPSL | 1.28E-10 | 1.070712 | 0.676 | 0.191 | 1.84E-06 |
| NFIA | 1.35E-10 | 0.488989 | 0.941 | 0.564 | 1.94E-06 |
| ARMH1 | 1.55E-10 | 0.314849 | 0.618 | 0.138 | 2.23E-06 |
| ARPC2 | 1.58E-10 | -0.73741 | 0.824 | 0.91 | 2.28E-06 |
| SOCS3 | 1.59E-10 | -1.02581 | 0 | 0.66 | 2.29E-06 |
| STMND1 | 1.65E-10 | 0.524748 | 0.618 | 0.144 | 2.37E-06 |
| GALNT5 | 1.68E-10 | -0.99672 | 0.118 | 0.729 | 2.42E-06 |
| ATP5IF1 | 1.71E-10 | 1.283655 | 0.941 | 0.888 | 2.46E-06 |
| TUBA4B | 1.71E-10 | 0.686018 | 0.647 | 0.149 | 2.46E-06 |
| TSNAXIP1 | 1.76E-10 | 0.331592 | 0.559 | 0.106 | 2.53E-06 |
| CFAP46 | 1.97E-10 | 0.514037 | 0.676 | 0.16 | 2.84E-06 |
| FHL2 | 2.10E-10 | -1.02512 | 0 | 0.654 | 3.02E-06 |
| YWHAZ | 2.15E-10 | -0.72842 | 0.912 | 0.963 | 3.09E-06 |
| CFAP44 | 2.20E-10 | 0.818526 | 0.706 | 0.218 | 3.16E-06 |
| SRGAP3-AS2 | 2.24E-10 | 0.933822 | 0.647 | 0.165 | 3.22E-06 |
| KDM1B | 2.39E-10 | 0.316546 | 0.588 | 0.128 | 3.44E-06 |
| MALL | 2.44E-10 | -1.16094 | 0.206 | 0.745 | 3.50E-06 |
| TEKT2 | 2.44E-10 | 0.626985 | 0.647 | 0.176 | 3.51E-06 |
| NLRP1 | 2.45E-10 | 0.345694 | 0.559 | 0.117 | 3.52E-06 |
| VWA3B | 2.50E-10 | 0.751863 | 0.647 | 0.176 | 3.60E-06 |
| WDPCP | 2.54E-10 | 0.327585 | 0.5 | 0.09 | 3.64E-06 |
| DUSP1 | 2.54E-10 | -1.23646 | 0.853 | 0.92 | 3.65E-06 |
| WDR35 | 2.58E-10 | 0.365059 | 0.618 | 0.149 | 3.70E-06 |
| CES1 | 2.66E-10 | 1.064334 | 0.853 | 0.41 | 3.82E-06 |
| SH3BGRL3 | 2.85E-10 | -1.14063 | 0.882 | 0.963 | 4.09E-06 |
| CCDC78 | 2.90E-10 | 1.164446 | 0.647 | 0.191 | 4.17E-06 |
| CAPS2 | 2.92E-10 | 0.377593 | 0.588 | 0.133 | 4.20E-06 |
| APH1B | 2.96E-10 | 0.251454 | 0.529 | 0.101 | 4.26E-06 |
| CYP4B1 | 3.01E-10 | 1.074625 | 0.971 | 0.883 | 4.33E-06 |
| JPT1 | 3.02E-10 | -0.89569 | 0.765 | 0.899 | 4.35E-06 |
| USP2 | 3.06E-10 | 0.281984 | 0.559 | 0.117 | 4.40E-06 |
| KIF27 | 3.11E-10 | 0.437031 | 0.618 | 0.165 | 4.46E-06 |
| DENND6B | 3.16E-10 | 0.452509 | 0.676 | 0.181 | 4.55E-06 |
| DNAAF4 | 3.23E-10 | 0.624966 | 0.676 | 0.213 | 4.64E-06 |
| MAFF | 3.36E-10 | -0.86854 | 0.206 | 0.739 | 4.82E-06 |
| TMSB4X | 3.38E-10 | -0.57601 | 0.971 | 0.989 | 4.86E-06 |
| S100A14 | 3.42E-10 | -1.45055 | 0.382 | 0.835 | 4.91E-06 |
| ZNF440 | 3.51E-10 | 0.485184 | 0.676 | 0.223 | 5.04E-06 |
| CFAP54 | 3.54E-10 | 0.52808 | 0.647 | 0.176 | 5.08E-06 |
| CYR61 | 3.63E-10 | -1.25332 | 0 | 0.644 | 5.21E-06 |
| C21orf58 | 3.76E-10 | 0.702114 | 0.618 | 0.176 | 5.41E-06 |
| CFL1 | 3.90E-10 | -0.78507 | 0.853 | 0.947 | 5.60E-06 |
| ZC2HC1A | 3.90E-10 | 0.595519 | 0.765 | 0.303 | 5.61E-06 |
| AL357093.2 | 3.91E-10 | 1.078179 | 0.618 | 0.144 | 5.62E-06 |
| MYB | 4.12E-10 | 0.256567 | 0.559 | 0.106 | 5.92E-06 |
| CROCC | 4.13E-10 | 0.752977 | 0.647 | 0.213 | 5.94E-06 |
| SPEF2 | 4.19E-10 | 0.855373 | 0.647 | 0.202 | 6.02E-06 |
| CFAP43 | 4.37E-10 | 0.933405 | 0.647 | 0.191 | 6.29E-06 |
| PRPF4B | 4.38E-10 | 0.42128 | 0.941 | 0.543 | 6.30E-06 |
| EPHX2 | 4.51E-10 | 0.288452 | 0.647 | 0.165 | 6.48E-06 |
| KCTD12 | 4.53E-10 | 0.530292 | 0.647 | 0.17 | 6.50E-06 |
| C1orf87 | 4.53E-10 | 0.382421 | 0.618 | 0.133 | 6.51E-06 |
| GAPDH | 4.72E-10 | -1.10915 | 1 | 0.973 | 6.79E-06 |
| RPS26 | 4.72E-10 | -0.69288 | 1 | 0.979 | 6.79E-06 |
| DNAH6 | 4.81E-10 | 0.751314 | 0.618 | 0.176 | 6.92E-06 |
| COL21A1 | 4.88E-10 | 0.331236 | 0.5 | 0.09 | 7.01E-06 |
| TTC29 | 4.97E-10 | 0.500813 | 0.588 | 0.138 | 7.14E-06 |
| C5AR1 | 4.98E-10 | 0.288069 | 0.647 | 0.165 | 7.16E-06 |
| KIF19 | 5.16E-10 | 0.358506 | 0.618 | 0.133 | 7.42E-06 |
| CCDC65 | 5.66E-10 | 0.56238 | 0.618 | 0.16 | 8.13E-06 |
| RIBC1 | 5.94E-10 | 0.362101 | 0.588 | 0.128 | 8.54E-06 |
| DNAH3 | 6.07E-10 | 0.779121 | 0.647 | 0.186 | 8.73E-06 |
| HMGA1 | 6.14E-10 | -1.01818 | 0.5 | 0.856 | 8.82E-06 |
| SERPINB2 | 6.20E-10 | -1.66262 | 0 | 0.633 | 8.91E-06 |
| RFX3 | 6.31E-10 | 0.779956 | 0.706 | 0.266 | 9.06E-06 |
| PMAIP1 | 6.33E-10 | -0.84513 | 0.118 | 0.697 | 9.10E-06 |
| ERICH5 | 6.41E-10 | 0.581524 | 0.647 | 0.191 | 9.21E-06 |
| PPP1R36 | 6.42E-10 | 0.360216 | 0.471 | 0.08 | 9.22E-06 |
| CCDC24 | 6.56E-10 | 0.512558 | 0.794 | 0.314 | 9.43E-06 |
| FYB2 | 6.63E-10 | 0.55973 | 0.735 | 0.277 | 9.52E-06 |
| CFAP45 | 6.83E-10 | 0.933361 | 0.647 | 0.207 | 9.81E-06 |
| THUMPD3-AS1 | 6.86E-10 | 0.444024 | 0.912 | 0.394 | 9.85E-06 |
| CC2D2A | 6.91E-10 | 0.72155 | 0.676 | 0.218 | 9.93E-06 |
| SNTN | 6.99E-10 | 1.391377 | 0.647 | 0.207 | 1.00E-05 |
| GRIN3B | 7.49E-10 | 0.293007 | 0.529 | 0.096 | 1.08E-05 |
| RSPH4A | 7.53E-10 | 0.793117 | 0.647 | 0.186 | 1.08E-05 |
| CCDC187 | 7.57E-10 | 0.692942 | 0.618 | 0.17 | 1.09E-05 |
| ANKRD18B | 7.59E-10 | 0.329866 | 0.529 | 0.101 | 1.09E-05 |
| KLF4 | 7.86E-10 | -1.09366 | 0.382 | 0.777 | 1.13E-05 |
| DRC1 | 7.90E-10 | 0.861942 | 0.647 | 0.186 | 1.14E-05 |
| SON | 7.98E-10 | 0.671093 | 0.971 | 0.915 | 1.15E-05 |
| ASS1 | 8.08E-10 | -1.23039 | 0.529 | 0.846 | 1.16E-05 |
| LRRC46 | 8.22E-10 | 1.106348 | 0.647 | 0.207 | 1.18E-05 |
| DDX3Y | 8.38E-10 | 0.251361 | 0.588 | 0.117 | 1.20E-05 |
| IQCD | 8.59E-10 | 0.67592 | 0.676 | 0.223 | 1.23E-05 |
| IQCA1 | 8.86E-10 | 0.428649 | 0.618 | 0.176 | 1.27E-05 |
| NIN | 8.89E-10 | 0.306936 | 0.676 | 0.218 | 1.28E-05 |
| FAM92B | 8.93E-10 | 0.900553 | 0.647 | 0.191 | 1.28E-05 |
| JADE1 | 8.96E-10 | 0.267313 | 0.559 | 0.122 | 1.29E-05 |
| KIAA1841 | 9.07E-10 | 0.483226 | 0.706 | 0.245 | 1.30E-05 |
| LINC00240 | 9.32E-10 | 0.265211 | 0.471 | 0.074 | 1.34E-05 |
| MAGIX | 9.51E-10 | 0.254295 | 0.618 | 0.165 | 1.37E-05 |
| CYB5D1 | 9.54E-10 | 0.36615 | 0.618 | 0.16 | 1.37E-05 |
| PRR29 | 1.03E-09 | 0.767194 | 0.647 | 0.191 | 1.48E-05 |
| CEP97 | 1.05E-09 | 0.296831 | 0.559 | 0.122 | 1.51E-05 |
| HLA-DMA | 1.08E-09 | 0.629098 | 0.941 | 0.612 | 1.55E-05 |
| CEP290 | 1.08E-09 | 0.641819 | 0.765 | 0.34 | 1.55E-05 |
| DMD | 1.13E-09 | 0.380967 | 0.676 | 0.207 | 1.63E-05 |
| MAP6 | 1.17E-09 | 0.367267 | 0.559 | 0.122 | 1.68E-05 |
| PPOX | 1.18E-09 | 0.464285 | 0.676 | 0.223 | 1.69E-05 |
| UPK1B | 1.18E-09 | -1.34211 | 0.176 | 0.734 | 1.70E-05 |
| TEX26 | 1.19E-09 | 0.476684 | 0.529 | 0.101 | 1.71E-05 |
| MYL12B | 1.22E-09 | -0.7783 | 0.941 | 0.968 | 1.76E-05 |
| S100A16 | 1.29E-09 | -1.31194 | 0.559 | 0.819 | 1.85E-05 |
| ALG1L | 1.30E-09 | 0.439479 | 0.647 | 0.191 | 1.87E-05 |
| MAATS1 | 1.31E-09 | 0.55917 | 0.618 | 0.165 | 1.88E-05 |
| DIXDC1 | 1.32E-09 | 0.291317 | 0.471 | 0.09 | 1.90E-05 |
| C2CD2L | 1.34E-09 | 0.311395 | 0.588 | 0.149 | 1.93E-05 |
| MSH3 | 1.43E-09 | 0.484791 | 0.706 | 0.255 | 2.06E-05 |
| C11orf88 | 1.44E-09 | 1.373546 | 0.676 | 0.255 | 2.07E-05 |
| CCDC170 | 1.45E-09 | 1.332625 | 0.706 | 0.298 | 2.09E-05 |
| SPAG17 | 1.55E-09 | 0.863799 | 0.647 | 0.218 | 2.23E-05 |
| GSTA1 | 1.57E-09 | 1.050005 | 0.941 | 0.67 | 2.26E-05 |
| GCC2 | 1.58E-09 | 0.595145 | 0.971 | 0.761 | 2.27E-05 |
| C7orf57 | 1.58E-09 | 0.397977 | 0.588 | 0.138 | 2.27E-05 |
| NT5DC1 | 1.72E-09 | 0.322564 | 0.765 | 0.324 | 2.47E-05 |
| EVL | 1.72E-09 | 0.430199 | 0.618 | 0.191 | 2.47E-05 |
| SMAP2 | 1.74E-09 | 0.316343 | 0.794 | 0.293 | 2.50E-05 |
| CCDC153 | 1.78E-09 | 0.646303 | 0.618 | 0.176 | 2.55E-05 |
| NSUN7 | 1.79E-09 | 0.558854 | 0.618 | 0.202 | 2.58E-05 |
| AC004832.1 | 1.80E-09 | 0.614774 | 0.676 | 0.223 | 2.59E-05 |
| SPATA24 | 1.81E-09 | 0.284613 | 0.412 | 0.064 | 2.59E-05 |
| IFT122 | 1.83E-09 | 0.370289 | 0.618 | 0.165 | 2.63E-05 |
| CD44 | 1.88E-09 | -0.77112 | 0.206 | 0.761 | 2.70E-05 |
| OLMALINC | 1.97E-09 | 0.252574 | 0.559 | 0.128 | 2.83E-05 |
| WDR49 | 1.97E-09 | 0.440767 | 0.706 | 0.229 | 2.84E-05 |
| PACRG | 2.11E-09 | 0.512867 | 0.559 | 0.138 | 3.04E-05 |
| ZBBX | 2.14E-09 | 0.790769 | 0.647 | 0.202 | 3.07E-05 |
| RAB5IF | 2.32E-09 | -0.65872 | 0.647 | 0.872 | 3.33E-05 |
| MNS1 | 2.43E-09 | 0.785331 | 0.647 | 0.213 | 3.49E-05 |
| CPLANE1 | 2.44E-09 | 0.402274 | 0.824 | 0.367 | 3.50E-05 |
| IFT80 | 2.51E-09 | 0.292134 | 0.618 | 0.165 | 3.60E-05 |
| WNT9A | 2.52E-09 | 0.344433 | 0.559 | 0.144 | 3.62E-05 |
| RND3 | 2.72E-09 | -0.81778 | 0.647 | 0.888 | 3.91E-05 |
| WDR63 | 2.78E-09 | 0.438436 | 0.647 | 0.176 | 4.00E-05 |
| CCDC146 | 2.84E-09 | 1.21208 | 0.676 | 0.282 | 4.08E-05 |
| LRIG1 | 2.85E-09 | 0.524194 | 0.853 | 0.426 | 4.09E-05 |
| TMEM231 | 2.88E-09 | 0.848948 | 0.706 | 0.239 | 4.14E-05 |
| OCEL1 | 2.91E-09 | 0.409844 | 0.794 | 0.309 | 4.19E-05 |
| CFAP47 | 2.92E-09 | 0.325177 | 0.559 | 0.122 | 4.20E-05 |
| RBM43 | 2.93E-09 | 0.296408 | 0.735 | 0.245 | 4.21E-05 |
| KLHDC9 | 3.05E-09 | 0.40713 | 0.588 | 0.16 | 4.38E-05 |
| IQCE | 3.09E-09 | 0.460929 | 0.647 | 0.207 | 4.44E-05 |
| SYNE1 | 3.14E-09 | 0.738745 | 0.647 | 0.202 | 4.51E-05 |
| CBX5 | 3.24E-09 | 0.514088 | 0.794 | 0.383 | 4.65E-05 |
| TMC5 | 3.41E-09 | 0.932362 | 0.912 | 0.809 | 4.90E-05 |
| DNAL1 | 3.43E-09 | 0.595061 | 0.676 | 0.245 | 4.92E-05 |
| SELENBP1 | 3.45E-09 | 0.675066 | 0.912 | 0.606 | 4.96E-05 |
| DNAH1 | 3.48E-09 | 0.383372 | 0.647 | 0.17 | 5.00E-05 |
| LRTOMT | 3.51E-09 | 0.454422 | 0.618 | 0.181 | 5.05E-05 |
| RAB36 | 3.53E-09 | 0.520618 | 0.588 | 0.138 | 5.07E-05 |
| ANKRD26 | 3.85E-09 | 0.345975 | 0.647 | 0.202 | 5.53E-05 |
| SPATA17 | 3.87E-09 | 0.543511 | 0.647 | 0.207 | 5.56E-05 |
| DALRD3 | 4.06E-09 | 0.425325 | 0.706 | 0.266 | 5.84E-05 |
| LDLRAD1 | 4.21E-09 | 0.866484 | 0.647 | 0.202 | 6.05E-05 |
| WDR78 | 4.36E-09 | 0.579463 | 0.647 | 0.197 | 6.27E-05 |
| MSI2 | 4.37E-09 | 0.495679 | 0.912 | 0.644 | 6.28E-05 |
| WDR66 | 4.59E-09 | 0.757211 | 0.647 | 0.223 | 6.59E-05 |
| MIR4435-2HG | 4.62E-09 | -0.88317 | 0.294 | 0.739 | 6.64E-05 |
| ALDH1L1 | 4.62E-09 | 0.269716 | 0.676 | 0.202 | 6.64E-05 |
| CFAP157 | 4.67E-09 | 1.186881 | 0.676 | 0.255 | 6.72E-05 |
| CAV2 | 4.96E-09 | -0.75681 | 0.294 | 0.729 | 7.12E-05 |
| PPP1R14B | 5.02E-09 | -0.64287 | 0.147 | 0.665 | 7.21E-05 |
| LRRIQ1 | 5.06E-09 | 1.063825 | 0.676 | 0.271 | 7.26E-05 |
| TEX9 | 5.11E-09 | 0.503433 | 0.706 | 0.266 | 7.34E-05 |
| BRD3OS | 5.20E-09 | 0.301892 | 0.588 | 0.149 | 7.47E-05 |
| VPS13C | 5.52E-09 | 0.449506 | 0.912 | 0.622 | 7.93E-05 |
| C4orf47 | 5.52E-09 | 0.668239 | 0.647 | 0.213 | 7.93E-05 |
| KIF3A | 5.52E-09 | 0.44933 | 0.647 | 0.213 | 7.93E-05 |
| LRRC49 | 5.63E-09 | 0.356645 | 0.559 | 0.149 | 8.10E-05 |
| FAM184A | 5.65E-09 | 0.271301 | 0.5 | 0.106 | 8.12E-05 |
| CFAP73 | 5.77E-09 | 0.754707 | 0.647 | 0.207 | 8.29E-05 |
| CCDC74A | 5.91E-09 | 0.646241 | 0.647 | 0.191 | 8.49E-05 |
| HLA-DQA1 | 5.95E-09 | 0.802591 | 0.765 | 0.282 | 8.55E-05 |
| CEP170 | 6.02E-09 | 0.254017 | 0.618 | 0.17 | 8.66E-05 |
| ARMC3 | 6.22E-09 | 0.858629 | 0.647 | 0.223 | 8.94E-05 |
| NR4A1 | 6.24E-09 | -0.87543 | 0 | 0.585 | 8.97E-05 |
| LMTK3 | 6.28E-09 | -0.88568 | 0.206 | 0.723 | 9.02E-05 |
| RPS12 | 6.40E-09 | -0.75795 | 0.971 | 0.995 | 9.20E-05 |
| ANKMY1 | 6.43E-09 | 0.582878 | 0.676 | 0.239 | 9.25E-05 |
| CCDC173 | 6.44E-09 | 0.65281 | 0.618 | 0.176 | 9.25E-05 |
| TRMT10A | 6.52E-09 | 0.281535 | 0.588 | 0.17 | 9.37E-05 |
| LEKR1 | 6.53E-09 | 0.295911 | 0.471 | 0.101 | 9.39E-05 |
| ZKSCAN1 | 6.69E-09 | 0.640489 | 0.882 | 0.585 | 9.61E-05 |
| DYNLRB2 | 6.75E-09 | 1.224529 | 0.676 | 0.271 | 9.71E-05 |
| RPL8 | 6.98E-09 | -0.68933 | 0.971 | 0.973 | 0.0001 |
| RSPH9 | 7.01E-09 | 0.754122 | 0.618 | 0.181 | 0.000101 |
| ODF2L | 7.22E-09 | 0.662896 | 0.912 | 0.676 | 0.000104 |
| IQCG | 7.24E-09 | 0.636737 | 0.735 | 0.277 | 0.000104 |
| PHC1 | 7.25E-09 | 0.287267 | 0.529 | 0.128 | 0.000104 |
| ICA1L | 7.55E-09 | 0.385629 | 0.588 | 0.165 | 0.000108 |
| RABL2A | 7.56E-09 | 0.453045 | 0.647 | 0.191 | 0.000109 |
| TMEM254 | 7.64E-09 | 0.331317 | 0.735 | 0.271 | 0.00011 |
| GPR162 | 7.84E-09 | 0.52213 | 0.647 | 0.181 | 0.000113 |
| IQCK | 8.06E-09 | 0.519123 | 0.765 | 0.314 | 0.000116 |
| AKAP9 | 8.33E-09 | 0.8841 | 0.941 | 0.793 | 0.00012 |
| ULK4 | 8.43E-09 | 0.302678 | 0.529 | 0.128 | 0.000121 |
| LRRC73 | 8.54E-09 | 0.275281 | 0.412 | 0.069 | 0.000123 |
| SPATA33 | 8.73E-09 | 0.491066 | 0.706 | 0.266 | 0.000125 |
| TNRC6B | 8.88E-09 | 0.560284 | 0.912 | 0.622 | 0.000128 |
| AC060780.1 | 9.04E-09 | 0.338387 | 0.5 | 0.112 | 0.00013 |
| PTGFR | 9.10E-09 | 0.277714 | 0.588 | 0.154 | 0.000131 |
| RPL24 | 9.11E-09 | -0.59862 | 1 | 0.963 | 0.000131 |
| PHTF1 | 9.26E-09 | 0.410779 | 0.706 | 0.261 | 0.000133 |
| RHEB | 9.86E-09 | -0.60461 | 0.765 | 0.883 | 0.000142 |
| TMEM67 | 1.01E-08 | 0.310809 | 0.618 | 0.181 | 0.000145 |
| WNK1 | 1.03E-08 | 0.444019 | 0.912 | 0.58 | 0.000148 |
| SPA17 | 1.04E-08 | 0.887162 | 0.647 | 0.239 | 0.000149 |
| DSG2 | 1.05E-08 | -0.6728 | 0.294 | 0.723 | 0.00015 |
| CDHR3 | 1.07E-08 | 0.9302 | 0.618 | 0.197 | 0.000153 |
| HLA-DMB | 1.10E-08 | 0.418888 | 0.676 | 0.245 | 0.000157 |
| CCDC40 | 1.14E-08 | 0.468555 | 0.618 | 0.186 | 0.000163 |
| DUSP5 | 1.16E-08 | -1.25267 | 0.176 | 0.707 | 0.000166 |
| CD55 | 1.17E-08 | -1.20203 | 0.794 | 0.888 | 0.000168 |
| CEP126 | 1.19E-08 | 0.73513 | 0.647 | 0.229 | 0.000172 |
| INTU | 1.20E-08 | 0.31843 | 0.676 | 0.213 | 0.000172 |
| GADD45B | 1.21E-08 | -1.02127 | 0.588 | 0.809 | 0.000174 |
| BAG1 | 1.22E-08 | -0.82736 | 0.824 | 0.91 | 0.000175 |
| GIPR | 1.26E-08 | 0.267152 | 0.559 | 0.138 | 0.000181 |
| FOXJ1 | 1.27E-08 | 0.829152 | 0.647 | 0.207 | 0.000182 |
| SNX29 | 1.27E-08 | 0.341928 | 0.559 | 0.154 | 0.000183 |
| ADAM9 | 1.28E-08 | -0.598 | 0.441 | 0.824 | 0.000184 |
| ADM | 1.30E-08 | -0.72993 | 0 | 0.569 | 0.000187 |
| AQP3 | 1.33E-08 | -1.19271 | 1 | 0.947 | 0.00019 |
| SULT1A1 | 1.33E-08 | 0.385092 | 0.824 | 0.335 | 0.000192 |
| FTO | 1.34E-08 | 0.553889 | 0.706 | 0.287 | 0.000193 |
| LRRC61 | 1.35E-08 | 0.446966 | 0.618 | 0.213 | 0.000194 |
| IQSEC1 | 1.42E-08 | 0.339372 | 0.647 | 0.218 | 0.000204 |
| PLS3 | 1.58E-08 | -0.73289 | 0.176 | 0.681 | 0.000227 |
| PLAUR | 1.63E-08 | -1.06082 | 0.118 | 0.644 | 0.000234 |
| RPL28 | 1.65E-08 | -0.5499 | 1 | 0.995 | 0.000238 |
| FANK1 | 1.67E-08 | 0.768667 | 0.647 | 0.245 | 0.00024 |
| ALDH1A3 | 1.73E-08 | -1.31582 | 0.529 | 0.777 | 0.000248 |
| IFT74 | 1.77E-08 | 0.351321 | 0.706 | 0.266 | 0.000255 |
| ILF3-DT | 1.79E-08 | 0.331198 | 0.647 | 0.202 | 0.000258 |
| C20orf85 | 1.80E-08 | 1.498875 | 0.647 | 0.261 | 0.000258 |
| NBPF1 | 1.81E-08 | 0.298592 | 0.559 | 0.154 | 0.00026 |
| CRIP1 | 1.82E-08 | 0.928065 | 0.882 | 0.654 | 0.000261 |
| CACNG6 | 1.91E-08 | 0.263515 | 0.382 | 0.059 | 0.000275 |
| MT3 | 1.94E-08 | 0.463182 | 0.529 | 0.133 | 0.000278 |
| PPIA | 1.94E-08 | -0.74409 | 0.971 | 0.952 | 0.000278 |
| OSCP1 | 1.99E-08 | 0.618994 | 0.676 | 0.25 | 0.000286 |
| MAPK15 | 2.02E-08 | 0.695587 | 0.647 | 0.229 | 0.00029 |
| IER3 | 2.02E-08 | -0.70275 | 0.088 | 0.633 | 0.000291 |
| FAM81B | 2.06E-08 | 0.757235 | 0.647 | 0.229 | 0.000296 |
| TTC39C | 2.09E-08 | 0.378605 | 0.735 | 0.303 | 0.0003 |
| CHST9 | 2.16E-08 | 0.779876 | 0.882 | 0.686 | 0.00031 |
| PIFO | 2.18E-08 | 1.1545 | 0.647 | 0.255 | 0.000314 |
| LZTFL1 | 2.19E-08 | 0.510059 | 0.676 | 0.261 | 0.000314 |
| ZNF487 | 2.19E-08 | 0.391449 | 0.676 | 0.223 | 0.000315 |
| COX7A2 | 2.23E-08 | -0.51741 | 0.941 | 0.963 | 0.000321 |
| BTBD9 | 2.30E-08 | 0.260123 | 0.588 | 0.17 | 0.00033 |
| ING5 | 2.30E-08 | 0.250732 | 0.618 | 0.186 | 0.000331 |
| ANK3 | 2.33E-08 | 0.551282 | 0.824 | 0.479 | 0.000334 |
| UBXN10 | 2.36E-08 | 0.569069 | 0.647 | 0.218 | 0.000339 |
| CFAP300 | 2.39E-08 | 0.640804 | 0.647 | 0.213 | 0.000343 |
| KREMEN1 | 2.42E-08 | 0.268774 | 0.529 | 0.138 | 0.000347 |
| AC244090.1 | 2.44E-08 | 0.556756 | 0.706 | 0.319 | 0.000351 |
| MT-ND5 | 2.45E-08 | 0.886203 | 0.971 | 0.995 | 0.000352 |
| TTBK2 | 2.54E-08 | 0.341349 | 0.559 | 0.16 | 0.000364 |
| CORO1C | 2.65E-08 | -0.54662 | 0 | 0.553 | 0.000381 |
| MXD1 | 2.65E-08 | -0.68588 | 0 | 0.553 | 0.000381 |
| LMO7 | 2.68E-08 | -1.04975 | 0.941 | 0.947 | 0.000385 |
| VDAC2 | 2.70E-08 | -0.74994 | 0.794 | 0.894 | 0.000388 |
| ACSS1 | 2.76E-08 | 0.297735 | 0.588 | 0.176 | 0.000397 |
| CHMP1B | 2.76E-08 | -0.58173 | 0.559 | 0.819 | 0.000397 |
| APPL2 | 2.92E-08 | 0.533644 | 0.706 | 0.33 | 0.000419 |
| PLEKHG7 | 2.92E-08 | 0.522166 | 0.706 | 0.298 | 0.000419 |
| ANKRD42 | 2.99E-08 | 0.251165 | 0.618 | 0.181 | 0.00043 |
| C9orf24 | 3.10E-08 | 1.424977 | 0.647 | 0.266 | 0.000446 |
| KRT4 | 3.13E-08 | -2.52373 | 0.118 | 0.617 | 0.000449 |
| PIH1D2 | 3.19E-08 | 0.32208 | 0.588 | 0.154 | 0.000458 |
| DDX24 | 3.20E-08 | 0.53238 | 0.971 | 0.739 | 0.00046 |
| ERRFI1 | 3.21E-08 | -1.03384 | 0.147 | 0.654 | 0.000461 |
| TP73 | 3.33E-08 | 0.291843 | 0.5 | 0.106 | 0.000479 |
| FOSL1 | 3.35E-08 | -0.62672 | 0 | 0.548 | 0.000481 |
| CNTRL | 3.40E-08 | 0.595411 | 0.706 | 0.309 | 0.000489 |
| CCDC189 | 3.45E-08 | 0.342869 | 0.647 | 0.181 | 0.000495 |
| RPL12 | 3.47E-08 | -0.71914 | 0.971 | 0.984 | 0.000499 |
| CLTB | 3.78E-08 | -0.81778 | 0.824 | 0.899 | 0.000543 |
| ROPN1L | 3.91E-08 | 0.869244 | 0.647 | 0.223 | 0.000561 |
| GNA14 | 3.91E-08 | 0.299346 | 0.559 | 0.16 | 0.000562 |
| DPCD | 3.97E-08 | 0.620208 | 0.706 | 0.298 | 0.00057 |
| PRPF6 | 4.08E-08 | 0.350473 | 0.882 | 0.559 | 0.000586 |
| RPGR | 4.10E-08 | 0.463517 | 0.588 | 0.191 | 0.000589 |
| BBOF1 | 4.16E-08 | 0.554462 | 0.706 | 0.266 | 0.000598 |
| ESPN | 4.30E-08 | 0.386472 | 0.529 | 0.154 | 0.000618 |
| ENKUR | 4.32E-08 | 0.813154 | 0.647 | 0.234 | 0.00062 |
| PFN1 | 4.41E-08 | -0.58052 | 0.912 | 0.979 | 0.000633 |
| HACD4 | 4.43E-08 | 0.494425 | 0.676 | 0.266 | 0.000636 |
| KIAA1211L | 4.50E-08 | 0.669037 | 0.765 | 0.388 | 0.000646 |
| DNAH7 | 4.61E-08 | 0.603602 | 0.618 | 0.218 | 0.000663 |
| RPLP2 | 4.71E-08 | -0.58891 | 1 | 0.973 | 0.000676 |
| ABI2 | 4.90E-08 | 0.310878 | 0.824 | 0.362 | 0.000704 |
| DZIP3 | 5.02E-08 | 0.570193 | 0.676 | 0.25 | 0.000721 |
| MOK | 5.09E-08 | 0.520772 | 0.647 | 0.229 | 0.000732 |
| CTXN1 | 5.19E-08 | 0.597829 | 0.647 | 0.218 | 0.000746 |
| PDE4DIP | 5.23E-08 | 0.408063 | 0.794 | 0.394 | 0.000751 |
| STEAP3 | 5.39E-08 | 0.520721 | 0.647 | 0.261 | 0.000775 |
| RIBC2 | 5.71E-08 | 0.384095 | 0.559 | 0.154 | 0.000821 |
| ZDHHC1 | 6.02E-08 | 0.601471 | 0.735 | 0.351 | 0.000866 |
| DNAAF1 | 6.06E-08 | 1.023156 | 0.647 | 0.255 | 0.00087 |
| ZMYND10 | 6.21E-08 | 0.820652 | 0.647 | 0.234 | 0.000892 |
| ZC3H6 | 6.24E-08 | 0.366659 | 0.618 | 0.234 | 0.000896 |
| HLA-DRA | 6.36E-08 | 0.957737 | 0.912 | 0.851 | 0.000913 |
| STIM2 | 6.47E-08 | 0.473904 | 0.912 | 0.559 | 0.00093 |
| CFAP77 | 6.62E-08 | 0.38522 | 0.588 | 0.16 | 0.000951 |
| ID1 | 6.69E-08 | -1.08728 | 0.147 | 0.649 | 0.000961 |
| TCTN1 | 6.70E-08 | 0.511061 | 0.765 | 0.351 | 0.000963 |
| RPL18 | 6.79E-08 | -0.54907 | 0.971 | 0.984 | 0.000975 |
| TPM4 | 7.06E-08 | -1.09653 | 0.706 | 0.878 | 0.001014 |
| NBEA | 7.09E-08 | 0.528458 | 0.735 | 0.34 | 0.00102 |
| CCDC114 | 7.20E-08 | 0.554745 | 0.647 | 0.218 | 0.001034 |
| DLEC1 | 7.41E-08 | 0.615927 | 0.618 | 0.202 | 0.001065 |
| SLFN13 | 7.65E-08 | 0.379663 | 0.647 | 0.223 | 0.001099 |
| TUBA1A | 7.87E-08 | 1.623242 | 0.706 | 0.388 | 0.001131 |
| SPAG6 | 8.05E-08 | 0.63475 | 0.647 | 0.234 | 0.001157 |
| MPHOSPH10 | 8.09E-08 | 0.307397 | 0.706 | 0.261 | 0.001162 |
| JUP | 8.13E-08 | -0.7336 | 0.618 | 0.872 | 0.001168 |
| PHGDH | 8.16E-08 | 0.360326 | 0.559 | 0.165 | 0.001173 |
| CALML4 | 8.29E-08 | 0.77376 | 0.706 | 0.319 | 0.001191 |
| KRT6A | 8.38E-08 | -1.75567 | 0 | 0.527 | 0.001205 |
| ORAI2 | 8.53E-08 | 0.319264 | 0.676 | 0.25 | 0.001226 |
| COIL | 8.58E-08 | 0.29849 | 0.618 | 0.207 | 0.001233 |
| METTL7A | 8.68E-08 | 0.567188 | 0.912 | 0.59 | 0.001248 |
| CCDC190 | 8.71E-08 | 0.628044 | 0.647 | 0.234 | 0.001252 |
| ATF7IP2 | 8.85E-08 | 0.459654 | 0.588 | 0.213 | 0.001272 |
| TMEM178A | 8.88E-08 | 0.250484 | 0.5 | 0.117 | 0.001277 |
| A4GALT | 8.93E-08 | -0.70793 | 0.294 | 0.734 | 0.001283 |
| EGR1 | 9.10E-08 | -1.24193 | 0.118 | 0.606 | 0.001308 |
| HLA-DPB1 | 9.36E-08 | 0.715878 | 0.882 | 0.574 | 0.001344 |
| ALOX15 | 9.42E-08 | 0.913371 | 0.824 | 0.5 | 0.001354 |
| JUNB | 9.48E-08 | -1.10745 | 0.618 | 0.84 | 0.001363 |
| ARFGEF3 | 9.54E-08 | 0.482179 | 0.882 | 0.606 | 0.001371 |
| RPS24 | 9.59E-08 | -0.5923 | 1 | 0.973 | 0.001377 |
| FOCAD | 9.84E-08 | 0.350093 | 0.676 | 0.245 | 0.001415 |
| B9D1 | 9.87E-08 | 0.693583 | 0.647 | 0.255 | 0.001418 |
| TCEA2 | 9.91E-08 | 0.250503 | 0.676 | 0.229 | 0.001424 |
| ABHD10 | 9.98E-08 | 0.260636 | 0.559 | 0.181 | 0.001434 |
| CCDC17 | 1.02E-07 | 0.862973 | 0.647 | 0.245 | 0.001464 |
| CETN2 | 1.02E-07 | 0.892918 | 0.735 | 0.388 | 0.001469 |
| SLC27A2 | 1.03E-07 | 0.525286 | 0.794 | 0.447 | 0.001482 |
| EPHA2 | 1.04E-07 | -0.63862 | 0.412 | 0.771 | 0.001491 |
| GCNT3 | 1.05E-07 | -0.62756 | 0 | 0.521 | 0.001508 |
| ARHGAP39 | 1.08E-07 | 0.36372 | 0.618 | 0.186 | 0.001545 |
| FAM183A | 1.08E-07 | 1.123599 | 0.647 | 0.271 | 0.001549 |
| CCDC181 | 1.08E-07 | 0.31914 | 0.5 | 0.122 | 0.001549 |
| SMARCA2 | 1.09E-07 | 0.432935 | 0.882 | 0.543 | 0.001565 |
| SOX2 | 1.09E-07 | 0.522464 | 0.912 | 0.622 | 0.001573 |
| SLC25A36 | 1.10E-07 | 0.312091 | 0.853 | 0.378 | 0.001585 |
| ALMS1 | 1.11E-07 | 0.306669 | 0.559 | 0.176 | 0.00159 |
| ZNF428 | 1.11E-07 | 0.426034 | 0.971 | 0.702 | 0.001602 |
| LPCAT4 | 1.13E-07 | -0.72261 | 0.529 | 0.793 | 0.001617 |
| NECTIN2 | 1.13E-07 | -0.6767 | 0.588 | 0.798 | 0.001622 |
| AC105446.1 | 1.13E-07 | 0.358875 | 0.412 | 0.08 | 0.001628 |
| ARL13B | 1.14E-07 | 0.262474 | 0.647 | 0.213 | 0.001642 |
| PRSS8 | 1.16E-07 | -0.73022 | 0.176 | 0.686 | 0.001673 |
| DDX17 | 1.18E-07 | 0.483154 | 0.971 | 0.92 | 0.001699 |
| CES4A | 1.20E-07 | 0.402257 | 0.559 | 0.17 | 0.00172 |
| C11orf74 | 1.22E-07 | 0.434603 | 0.676 | 0.245 | 0.001751 |
| LCA5 | 1.22E-07 | 0.482301 | 0.618 | 0.229 | 0.001758 |
| SGK1 | 1.23E-07 | -0.84718 | 0.353 | 0.766 | 0.001775 |
| CHMP4B | 1.24E-07 | -0.59534 | 0.912 | 0.888 | 0.001787 |
| RAC1 | 1.27E-07 | -0.48154 | 0.941 | 0.973 | 0.001819 |
| HMGN3 | 1.27E-07 | 0.721558 | 0.971 | 0.856 | 0.001825 |
| LRRC45 | 1.28E-07 | 0.296581 | 0.588 | 0.186 | 0.00184 |
| GSTA2 | 1.31E-07 | 1.16643 | 0.706 | 0.335 | 0.001878 |
| C1orf194 | 1.33E-07 | 1.082488 | 0.647 | 0.271 | 0.001909 |
| MT-ND6 | 1.35E-07 | 1.094915 | 0.971 | 0.862 | 0.001935 |
| LRRC23 | 1.38E-07 | 1.056698 | 0.706 | 0.388 | 0.001986 |
| PRKAR1A | 1.40E-07 | 0.570326 | 0.971 | 0.851 | 0.002017 |
| CLDN7 | 1.48E-07 | -0.64056 | 0.971 | 0.941 | 0.002127 |
| MAP1B | 1.49E-07 | 0.260636 | 0.559 | 0.138 | 0.002147 |
| KRT19 | 1.50E-07 | -0.87762 | 0.971 | 0.979 | 0.002148 |
| GLUD1 | 1.53E-07 | -0.61607 | 0.676 | 0.814 | 0.002198 |
| CFAP126 | 1.55E-07 | 0.723644 | 0.647 | 0.239 | 0.002232 |
| IFT43 | 1.57E-07 | 0.758254 | 0.794 | 0.548 | 0.002253 |
| PLEKHS1 | 1.58E-07 | 0.614853 | 0.912 | 0.633 | 0.002267 |
| PNPLA2 | 1.62E-07 | -0.66794 | 0.588 | 0.83 | 0.002323 |
| AL022068.1 | 1.63E-07 | 0.255712 | 0.471 | 0.112 | 0.002348 |
| ARL5B | 1.64E-07 | -0.38118 | 0 | 0.511 | 0.00235 |
| GJB3 | 1.64E-07 | -0.44963 | 0 | 0.511 | 0.00235 |
| LAD1 | 1.64E-07 | -0.47842 | 0 | 0.511 | 0.00235 |
| AP001207.3 | 1.67E-07 | 0.323012 | 0.5 | 0.149 | 0.002401 |
| CFAP100 | 1.68E-07 | 0.64687 | 0.647 | 0.229 | 0.002417 |
| SPATA18 | 1.69E-07 | 0.708117 | 0.676 | 0.282 | 0.002426 |
| SYNE2 | 1.73E-07 | 0.68249 | 0.882 | 0.702 | 0.002481 |
| LMNA | 1.73E-07 | -0.66464 | 0.853 | 0.915 | 0.002485 |
| MCL1 | 1.76E-07 | -0.63379 | 0.735 | 0.92 | 0.002529 |
| AKNA | 1.85E-07 | 0.446969 | 0.618 | 0.229 | 0.002658 |
| SERPINB5 | 1.86E-07 | -0.62904 | 0.088 | 0.574 | 0.002676 |
| RPLP0 | 1.92E-07 | -0.70537 | 1 | 0.979 | 0.002762 |
| RPL36A | 2.03E-07 | -0.696 | 0.912 | 0.915 | 0.002915 |
| IER5 | 2.04E-07 | -0.37514 | 0 | 0.505 | 0.002926 |
| SPNS2 | 2.04E-07 | -0.82091 | 0 | 0.505 | 0.002926 |
| KRT15 | 2.06E-07 | -0.98843 | 0.265 | 0.686 | 0.002956 |
| EP400 | 2.14E-07 | 0.411702 | 0.735 | 0.356 | 0.003081 |
| EVI5 | 2.19E-07 | 0.276682 | 0.765 | 0.319 | 0.003143 |
| FGF14 | 2.21E-07 | 0.302167 | 0.382 | 0.069 | 0.003181 |
| IFT140 | 2.24E-07 | 0.358287 | 0.588 | 0.181 | 0.003213 |
| CHD4 | 2.25E-07 | 0.432896 | 0.912 | 0.585 | 0.003235 |
| UBXN11 | 2.26E-07 | 0.657951 | 0.735 | 0.362 | 0.00325 |
| TP53BP1 | 2.29E-07 | 0.416063 | 0.706 | 0.319 | 0.003286 |
| TRIB1 | 2.30E-07 | -0.59834 | 0.265 | 0.66 | 0.00331 |
| 10-Mar | 2.45E-07 | 0.373596 | 0.559 | 0.165 | 0.003526 |
| CEP83 | 2.46E-07 | 0.383341 | 0.618 | 0.245 | 0.003542 |
| SLC22A23 | 2.49E-07 | 0.367162 | 0.706 | 0.372 | 0.003574 |
| PLCH1 | 2.51E-07 | 0.273382 | 0.529 | 0.154 | 0.003606 |
| CSRNP1 | 2.51E-07 | -0.54273 | 0.206 | 0.676 | 0.003614 |
| EFNA1 | 2.53E-07 | -0.39332 | 0 | 0.5 | 0.003636 |
| LURAP1L | 2.53E-07 | -0.5533 | 0 | 0.5 | 0.003636 |
| KRT13 | 2.53E-07 | -1.53773 | 0 | 0.5 | 0.003636 |
| RPL35 | 2.54E-07 | -0.58725 | 1 | 0.963 | 0.003653 |
| NENF | 2.56E-07 | -0.55214 | 0.676 | 0.83 | 0.003685 |
| IFT88 | 2.71E-07 | 0.448769 | 0.647 | 0.271 | 0.003888 |
| CCDC88C | 2.74E-07 | 0.494798 | 0.824 | 0.489 | 0.003942 |
| EMB | 2.75E-07 | 0.432344 | 0.765 | 0.404 | 0.003956 |
| LINC00342 | 2.77E-07 | -0.64721 | 0.294 | 0.745 | 0.003981 |
| ENPP5 | 2.81E-07 | 0.354419 | 0.588 | 0.202 | 0.004036 |
| ANXA3 | 2.81E-07 | -0.86684 | 0.324 | 0.723 | 0.004044 |
| C9orf116 | 2.83E-07 | 0.946424 | 0.676 | 0.293 | 0.004061 |
| DHX32 | 2.85E-07 | 0.486885 | 0.794 | 0.431 | 0.004096 |
| HIPK2 | 2.86E-07 | 0.424157 | 0.824 | 0.436 | 0.004106 |
| PTPRF | 2.87E-07 | 0.584843 | 0.824 | 0.622 | 0.004117 |
| AC008915.2 | 2.92E-07 | 0.277167 | 0.559 | 0.176 | 0.004196 |
| EMC2 | 3.08E-07 | 0.340052 | 0.706 | 0.303 | 0.00442 |
| NPIPA1 | 3.11E-07 | 0.292532 | 0.559 | 0.17 | 0.004465 |
| MAPRE3 | 3.13E-07 | 0.376511 | 0.588 | 0.197 | 0.004504 |
| C2orf74 | 3.14E-07 | 0.260162 | 0.559 | 0.186 | 0.004509 |
| PRSS22 | 3.17E-07 | -0.8441 | 0.765 | 0.835 | 0.004551 |
| PRRC2B | 3.19E-07 | 0.444869 | 0.912 | 0.644 | 0.004583 |
| IGFBP7 | 3.24E-07 | 1.318261 | 0.647 | 0.298 | 0.00465 |
| TTLL5 | 3.24E-07 | 0.295566 | 0.647 | 0.229 | 0.004661 |
| RPL18A | 3.41E-07 | -0.67076 | 1 | 0.968 | 0.004896 |
| PSIP1 | 3.42E-07 | 0.459374 | 0.735 | 0.367 | 0.004907 |
| LKAAEAR1 | 3.43E-07 | 0.345711 | 0.382 | 0.074 | 0.00493 |
| BX284668.5 | 3.46E-07 | 0.362828 | 0.676 | 0.298 | 0.004979 |
| ANKRD37 | 3.48E-07 | 0.597313 | 0.676 | 0.319 | 0.004997 |
| PKP3 | 3.50E-07 | -0.48673 | 0.382 | 0.713 | 0.005036 |
| RSPH1 | 3.52E-07 | 1.30819 | 0.676 | 0.356 | 0.00506 |
| FAM161A | 3.59E-07 | 0.282035 | 0.529 | 0.149 | 0.005156 |
| UPF3A | 3.62E-07 | 0.357528 | 0.853 | 0.473 | 0.0052 |
| C12orf75 | 3.67E-07 | 0.793661 | 0.676 | 0.319 | 0.005269 |
| CD164L2 | 3.80E-07 | 0.321866 | 0.588 | 0.186 | 0.005462 |
| ZNHIT2 | 3.84E-07 | 0.348058 | 0.647 | 0.271 | 0.005514 |
| METTL27 | 3.84E-07 | 0.48804 | 0.647 | 0.261 | 0.005516 |
| SMYD2 | 3.84E-07 | 0.398719 | 0.706 | 0.309 | 0.005517 |
| ENOSF1 | 3.97E-07 | 0.382683 | 0.824 | 0.42 | 0.005707 |
| TUBB4B | 4.01E-07 | 1.594136 | 0.971 | 0.915 | 0.00576 |
| PFDN5 | 4.02E-07 | 0.484035 | 0.941 | 0.936 | 0.005775 |
| NEK11 | 4.04E-07 | 0.454805 | 0.588 | 0.213 | 0.005811 |
| PATL2 | 4.05E-07 | 0.261662 | 0.588 | 0.181 | 0.005814 |
| UBB | 4.08E-07 | 0.578947 | 0.971 | 0.936 | 0.005867 |
| PPP1R32 | 4.11E-07 | 0.467096 | 0.471 | 0.122 | 0.005905 |
| RSBN1L | 4.19E-07 | 0.299519 | 0.941 | 0.553 | 0.006024 |
| BTBD3 | 4.20E-07 | 0.348791 | 0.588 | 0.213 | 0.006042 |
| TTC3 | 4.21E-07 | 0.405556 | 0.912 | 0.66 | 0.006053 |
| KRT23 | 4.29E-07 | -1.36885 | 0.588 | 0.793 | 0.006168 |
| MYADM | 4.48E-07 | -0.72761 | 0.5 | 0.782 | 0.006438 |
| C10orf95 | 4.65E-07 | 0.356512 | 0.529 | 0.17 | 0.006683 |
| TCTEX1D2 | 4.73E-07 | 0.42367 | 0.706 | 0.277 | 0.006796 |
| SLAIN2 | 4.76E-07 | 0.496452 | 0.765 | 0.473 | 0.006844 |
| EEF1D | 4.79E-07 | -0.50606 | 0.971 | 0.931 | 0.006877 |
| LY6D | 4.80E-07 | -1.31948 | 0 | 0.484 | 0.006903 |
| ISG20 | 4.83E-07 | -0.89617 | 0.971 | 0.936 | 0.006934 |
| PTPRN2 | 4.87E-07 | 0.333902 | 0.765 | 0.372 | 0.007005 |
| PPL | 5.05E-07 | -0.63298 | 0.529 | 0.835 | 0.00725 |
| ATXN2 | 5.08E-07 | 0.391477 | 0.853 | 0.511 | 0.0073 |
| C11orf49 | 5.11E-07 | 0.353003 | 0.706 | 0.314 | 0.007347 |
| FAM229B | 5.13E-07 | 0.771612 | 0.706 | 0.34 | 0.007377 |
| MAPK10 | 5.14E-07 | 0.458386 | 0.765 | 0.383 | 0.007388 |
| SPACA9 | 5.23E-07 | 0.517124 | 0.676 | 0.266 | 0.00751 |
| MAP9 | 5.78E-07 | 0.565951 | 0.706 | 0.367 | 0.008306 |
| RAP2B | 5.84E-07 | -0.49947 | 0.176 | 0.617 | 0.008386 |
| LDLR | 5.92E-07 | -0.62101 | 0.441 | 0.798 | 0.008507 |
| FAM95C | 5.98E-07 | 0.27853 | 0.382 | 0.085 | 0.008594 |
| C1orf116 | 6.00E-07 | -0.90744 | 0.618 | 0.787 | 0.008621 |
| KLF5 | 6.04E-07 | -0.67633 | 0.882 | 0.936 | 0.008675 |
| GDF15 | 6.08E-07 | -1.28164 | 0.382 | 0.723 | 0.008738 |
| AC234582.1 | 6.17E-07 | 0.258661 | 0.588 | 0.181 | 0.008866 |
| WDR60 | 6.32E-07 | 0.623367 | 0.706 | 0.372 | 0.009082 |
| PPIL6 | 6.57E-07 | 0.633062 | 0.647 | 0.266 | 0.009439 |
| CLMN | 6.64E-07 | 0.531236 | 0.794 | 0.521 | 0.009535 |
| TTC21A | 6.77E-07 | 0.330513 | 0.588 | 0.197 | 0.009731 |
| TMEM154 | 6.77E-07 | 0.494693 | 0.676 | 0.33 | 0.009734 |
| LSR | 6.84E-07 | -0.56429 | 0.706 | 0.84 | 0.009826 |
| SQLE | 6.87E-07 | 0.426938 | 0.765 | 0.415 | 0.009871 |
| RALGAPA2 | 7.04E-07 | 0.366595 | 0.853 | 0.532 | 0.010121 |
| RPS21 | 7.05E-07 | -0.52206 | 0.971 | 0.989 | 0.010125 |
| PPA1 | 7.12E-07 | -0.56346 | 0.765 | 0.878 | 0.010228 |
| SPINT2 | 7.30E-07 | -0.44715 | 0.971 | 0.941 | 0.01049 |
| ARNTL2 | 7.30E-07 | -0.38427 | 0 | 0.473 | 0.01049 |
| MRNIP | 7.32E-07 | 0.356453 | 0.588 | 0.218 | 0.01052 |
| FUT3 | 7.39E-07 | -0.57533 | 0.265 | 0.649 | 0.01062 |
| PPP1R16A | 7.46E-07 | 0.400354 | 0.824 | 0.457 | 0.010718 |
| CRB3 | 7.47E-07 | -0.58376 | 0.441 | 0.723 | 0.010728 |
| SDC1 | 7.57E-07 | -0.58037 | 0.265 | 0.734 | 0.01088 |
| TRAPPC12 | 7.58E-07 | 0.259507 | 0.794 | 0.378 | 0.010889 |
| ETS2 | 7.60E-07 | -0.75521 | 0.529 | 0.766 | 0.010925 |
| LRWD1 | 7.69E-07 | 0.286785 | 0.647 | 0.245 | 0.011055 |
| SAA2-SAA4 | 7.80E-07 | 0.315141 | 0.353 | 0.069 | 0.011209 |
| SMIM19 | 7.82E-07 | 0.45259 | 0.824 | 0.569 | 0.011235 |
| SLC25A25 | 7.94E-07 | -0.54746 | 0.265 | 0.67 | 0.011409 |
| HLA-DQB1 | 7.94E-07 | 0.685381 | 0.765 | 0.447 | 0.01141 |
| NUCB2 | 7.97E-07 | 1.027289 | 0.941 | 0.862 | 0.011458 |
| CTNNAL1 | 7.98E-07 | 0.30466 | 0.676 | 0.282 | 0.011471 |
| PSENEN | 8.38E-07 | 0.718099 | 0.882 | 0.569 | 0.012047 |
| RPL26 | 8.42E-07 | -0.56307 | 0.941 | 0.979 | 0.012103 |
| MS4A8 | 8.54E-07 | 0.936225 | 0.735 | 0.495 | 0.012274 |
| SPATA6 | 8.67E-07 | 0.459844 | 0.618 | 0.271 | 0.012461 |
| PIBF1 | 8.76E-07 | 0.384898 | 0.676 | 0.314 | 0.012593 |
| DUSP18 | 8.82E-07 | 0.276457 | 0.618 | 0.229 | 0.012674 |
| SCEL | 8.98E-07 | -0.51324 | 0 | 0.468 | 0.012899 |
| CLUAP1 | 9.02E-07 | 0.496157 | 0.706 | 0.351 | 0.012957 |
| SUDS3 | 9.05E-07 | 0.37248 | 0.853 | 0.574 | 0.013001 |
| CYB561 | 9.08E-07 | 0.489578 | 0.853 | 0.58 | 0.013042 |
| VCL | 9.41E-07 | -0.52003 | 0.235 | 0.707 | 0.013518 |
| FGD5-AS1 | 9.60E-07 | 0.367936 | 0.853 | 0.516 | 0.013788 |
| EIF6 | 9.61E-07 | -0.59655 | 0.794 | 0.846 | 0.013811 |
| ZNF106 | 9.85E-07 | 0.80313 | 0.794 | 0.617 | 0.014153 |
| EFHC1 | 9.91E-07 | 0.960674 | 0.647 | 0.314 | 0.014245 |
| MZF1 | 1.00E-06 | 0.25939 | 0.765 | 0.314 | 0.014384 |
| TMBIM1 | 1.01E-06 | -0.62703 | 0.471 | 0.819 | 0.014487 |
| FAM104B | 1.02E-06 | 0.414357 | 0.676 | 0.33 | 0.014629 |
| IFT27 | 1.05E-06 | 0.628607 | 0.735 | 0.447 | 0.015144 |
| RPGRIP1L | 1.08E-06 | 0.293578 | 0.471 | 0.144 | 0.015456 |
| SERINC2 | 1.08E-06 | -0.54255 | 0.618 | 0.824 | 0.015564 |
| ARF6 | 1.09E-06 | -0.58933 | 0.824 | 0.846 | 0.015724 |
| SMAGP | 1.10E-06 | -0.46178 | 0.235 | 0.66 | 0.015813 |
| GPR87 | 1.10E-06 | -0.3345 | 0 | 0.463 | 0.015834 |
| MYC | 1.10E-06 | -0.54874 | 0 | 0.463 | 0.015834 |
| TMEM131 | 1.11E-06 | 0.361465 | 0.735 | 0.383 | 0.015982 |
| VNN3 | 1.12E-06 | 0.461371 | 0.559 | 0.202 | 0.016074 |
| DYDC2 | 1.12E-06 | 0.277599 | 0.529 | 0.144 | 0.016111 |
| TRAF3IP1 | 1.13E-06 | 0.485773 | 0.647 | 0.298 | 0.016234 |
| RHOD | 1.14E-06 | -0.67145 | 0.382 | 0.691 | 0.016452 |
| GAPVD1 | 1.15E-06 | 0.45044 | 0.824 | 0.484 | 0.016525 |
| SAMHD1 | 1.17E-06 | 0.521627 | 1 | 0.824 | 0.016808 |
| CDH26 | 1.24E-06 | 0.335367 | 0.706 | 0.346 | 0.017884 |
| CDC16 | 1.29E-06 | 0.425381 | 0.735 | 0.452 | 0.018467 |
| RAD9A | 1.29E-06 | 0.299389 | 0.706 | 0.314 | 0.018501 |
| RORC | 1.31E-06 | 0.268091 | 0.618 | 0.234 | 0.018844 |
| PHLDA3 | 1.32E-06 | -0.45564 | 0.118 | 0.564 | 0.018905 |
| POMP | 1.32E-06 | -0.44644 | 0.853 | 0.947 | 0.019031 |
| ABRACL | 1.33E-06 | -0.56952 | 0.471 | 0.755 | 0.01908 |
| ETHE1 | 1.34E-06 | -0.47461 | 0.235 | 0.66 | 0.019256 |
| AC008397.1 | 1.35E-06 | -0.28223 | 0 | 0.457 | 0.019403 |
| NFIC | 1.35E-06 | 0.44343 | 0.971 | 0.755 | 0.019406 |
| RRAD | 1.36E-06 | 0.579963 | 0.647 | 0.261 | 0.019477 |
| UVSSA | 1.36E-06 | 0.277816 | 0.647 | 0.25 | 0.019545 |
| NDUFA2 | 1.38E-06 | 0.399749 | 0.941 | 0.777 | 0.019815 |
| YWHAE | 1.39E-06 | 0.393254 | 1 | 0.931 | 0.019908 |
| H3F3B | 1.39E-06 | -0.58311 | 1 | 0.995 | 0.019956 |
| SQSTM1 | 1.41E-06 | -0.70047 | 0.971 | 0.947 | 0.020219 |
| C9orf135 | 1.43E-06 | 0.527003 | 0.647 | 0.234 | 0.020602 |
| ENDOG | 1.44E-06 | 0.441734 | 0.676 | 0.346 | 0.02071 |
| KIAA0232 | 1.44E-06 | 0.412883 | 0.735 | 0.426 | 0.020736 |
| TJP3 | 1.45E-06 | 0.408377 | 0.912 | 0.612 | 0.020801 |
| FARP1 | 1.45E-06 | 0.457826 | 0.853 | 0.479 | 0.020816 |
| KIAA1671 | 1.46E-06 | 0.327214 | 0.765 | 0.415 | 0.020932 |
| CSPP1 | 1.46E-06 | 0.632404 | 0.676 | 0.34 | 0.020995 |
| CCDC66 | 1.51E-06 | 0.26967 | 0.647 | 0.266 | 0.02172 |
| MGST1 | 1.52E-06 | -0.73456 | 0.676 | 0.883 | 0.021863 |
| ANKRD36C | 1.52E-06 | -0.64016 | 0.5 | 0.798 | 0.021888 |
| PCM1 | 1.54E-06 | 0.73514 | 0.853 | 0.707 | 0.022116 |
| ALKBH5 | 1.55E-06 | 0.3586 | 0.824 | 0.516 | 0.022231 |
| UNC119B | 1.56E-06 | 0.340352 | 0.588 | 0.223 | 0.022397 |
| GLRX | 1.56E-06 | -0.81112 | 0.853 | 0.888 | 0.022472 |
| HSP90AB1 | 1.61E-06 | 0.740851 | 1 | 0.968 | 0.02307 |
| ZC3H13 | 1.64E-06 | 0.513735 | 0.765 | 0.399 | 0.023529 |
| KIAA0319L | 1.64E-06 | 0.475569 | 0.882 | 0.59 | 0.023541 |
| PRDX5 | 1.65E-06 | 0.95166 | 0.941 | 0.947 | 0.023708 |
| TLE1 | 1.65E-06 | -0.31181 | 0 | 0.452 | 0.023738 |
| RPS5 | 1.68E-06 | -0.52526 | 0.971 | 0.957 | 0.02408 |
| RPS15A | 1.68E-06 | -0.50431 | 0.941 | 0.989 | 0.024097 |
| MICAL3 | 1.68E-06 | 0.41575 | 0.588 | 0.213 | 0.024136 |
| CHST6 | 1.68E-06 | 0.468845 | 0.735 | 0.362 | 0.024199 |
| NAA40 | 1.69E-06 | 0.266317 | 0.529 | 0.165 | 0.024298 |
| RBL2 | 1.74E-06 | 0.322801 | 0.765 | 0.383 | 0.025003 |
| IL20RA | 1.77E-06 | 0.368015 | 0.676 | 0.319 | 0.025415 |
| LRRC6 | 1.78E-06 | 0.382365 | 0.618 | 0.234 | 0.02561 |
| PFDN6 | 1.80E-06 | 0.336372 | 0.853 | 0.527 | 0.025831 |
| UTRN | 1.80E-06 | 0.397409 | 0.765 | 0.41 | 0.025898 |
| CERS4 | 1.85E-06 | 0.441533 | 0.618 | 0.282 | 0.026519 |
| RPS16 | 1.85E-06 | -0.5064 | 0.971 | 0.957 | 0.026639 |
| EPS8L1 | 1.86E-06 | -0.57149 | 0.706 | 0.856 | 0.026735 |
| HMGB1 | 1.90E-06 | 0.345289 | 1 | 0.899 | 0.027237 |
| GRAMD1C | 1.93E-06 | 0.281509 | 0.559 | 0.181 | 0.027765 |
| LGALS3 | 1.94E-06 | -0.53626 | 1 | 0.984 | 0.027835 |
| TRIM56 | 1.97E-06 | 0.651916 | 0.882 | 0.67 | 0.028371 |
| ANXA11 | 2.02E-06 | -0.55156 | 0.941 | 0.931 | 0.028957 |
| CDKN1A | 2.04E-06 | -0.87728 | 0.706 | 0.819 | 0.029321 |
| EFCAB2 | 2.07E-06 | 0.48323 | 0.676 | 0.324 | 0.029717 |
| IQANK1 | 2.21E-06 | 0.31363 | 0.676 | 0.293 | 0.031763 |
| UBE2D2 | 2.24E-06 | -0.46232 | 0.618 | 0.819 | 0.032241 |
| FAM13A | 2.27E-06 | 0.434327 | 0.676 | 0.356 | 0.032663 |
| IFT81 | 2.30E-06 | 0.458895 | 0.647 | 0.298 | 0.033122 |
| TOGARAM1 | 2.31E-06 | 0.345906 | 0.559 | 0.223 | 0.033242 |
| DSE | 2.34E-06 | 0.29849 | 0.706 | 0.309 | 0.033651 |
| C5orf49 | 2.34E-06 | 0.843536 | 0.647 | 0.298 | 0.033681 |
| CSTA | 2.35E-06 | -1.036 | 0.382 | 0.745 | 0.03372 |
| FAM3B | 2.36E-06 | -0.46642 | 0.176 | 0.569 | 0.033868 |
| IRX3 | 2.43E-06 | 0.588753 | 0.735 | 0.436 | 0.034932 |
| LYPD2 | 2.44E-06 | -2.5268 | 0.294 | 0.67 | 0.035038 |
| DYNC2LI1 | 2.47E-06 | 0.456105 | 0.676 | 0.346 | 0.035455 |
| HSD11B1L | 2.54E-06 | 0.29591 | 0.412 | 0.112 | 0.036531 |
| GAS8 | 2.56E-06 | 0.340864 | 0.647 | 0.282 | 0.036828 |
| ZBTB20 | 2.62E-06 | 0.26894 | 0.794 | 0.426 | 0.037714 |
| LAMTOR1 | 2.63E-06 | -0.41832 | 0.706 | 0.835 | 0.037789 |
| CAT | 2.66E-06 | 0.285946 | 0.647 | 0.293 | 0.038208 |
| MET | 2.66E-06 | -0.55872 | 0.5 | 0.83 | 0.038267 |
| PPP1R15A | 2.68E-06 | -0.76611 | 0.706 | 0.846 | 0.038447 |
| CCDC57 | 2.71E-06 | 0.280797 | 0.853 | 0.505 | 0.038873 |
| NEK1 | 2.71E-06 | 0.326985 | 0.559 | 0.239 | 0.039002 |
| VSTM2L | 2.76E-06 | 0.472173 | 0.735 | 0.431 | 0.039674 |
| RHPN1 | 2.79E-06 | 0.350036 | 0.676 | 0.293 | 0.040125 |
| RPS23 | 2.81E-06 | -0.52917 | 0.941 | 0.984 | 0.040335 |
| ST14 | 2.90E-06 | -0.51294 | 0.882 | 0.904 | 0.041626 |
| CDS1 | 2.99E-06 | 0.479249 | 0.853 | 0.585 | 0.042911 |
| SNRPN | 2.99E-06 | 0.28202 | 0.706 | 0.346 | 0.042954 |
| SIK1B | 2.99E-06 | -0.33057 | 0 | 0.436 | 0.043037 |
| KRT5 | 2.99E-06 | -1.21941 | 0 | 0.436 | 0.043037 |
| TUBGCP2 | 3.04E-06 | 0.393936 | 0.824 | 0.484 | 0.043632 |
| MYCBP | 3.06E-06 | 0.437774 | 0.735 | 0.436 | 0.043932 |
| KIF21A | 3.20E-06 | 0.812508 | 0.824 | 0.782 | 0.046049 |
| TMED2 | 3.24E-06 | -0.42132 | 0.735 | 0.856 | 0.046511 |
| LYRM2 | 3.32E-06 | 0.282999 | 0.882 | 0.516 | 0.047742 |
| VEGFA | 3.40E-06 | -0.3844 | 0.088 | 0.511 | 0.048817 |
| DHRS7B | 3.43E-06 | 0.273883 | 0.794 | 0.415 | 0.04932 |
| MSLN | 3.49E-06 | -0.67497 | 0.265 | 0.681 | 0.050181 |
| CEACAM6 | 3.53E-06 | -1.06413 | 0.735 | 0.851 | 0.050761 |
| CYTOR | 3.58E-06 | -0.48158 | 0.147 | 0.537 | 0.051396 |
| RPS3 | 3.59E-06 | -0.6261 | 0.971 | 0.973 | 0.051613 |
| RPS19 | 3.62E-06 | -0.55022 | 1 | 1 | 0.051987 |
| RPS28 | 3.67E-06 | -0.47718 | 1 | 0.989 | 0.05272 |
| SSBP4 | 3.70E-06 | 0.460541 | 0.735 | 0.441 | 0.053163 |
| KLK11 | 3.72E-06 | -0.58867 | 0.265 | 0.734 | 0.053435 |
| MYL6 | 3.72E-06 | -0.599 | 1 | 0.984 | 0.053463 |
| BSCL2 | 3.73E-06 | 0.481307 | 0.706 | 0.41 | 0.053645 |
| GIGYF2 | 3.77E-06 | 0.304993 | 0.676 | 0.362 | 0.054127 |
| OXTR | 3.79E-06 | 0.463496 | 0.412 | 0.112 | 0.054515 |
| RPL41 | 3.80E-06 | -0.41743 | 0.971 | 0.995 | 0.054598 |
| RAB25 | 3.81E-06 | -0.54092 | 0.824 | 0.862 | 0.054687 |
| ATP6V1G1 | 3.98E-06 | -0.41973 | 0.941 | 0.947 | 0.057236 |
| FAM129B | 4.04E-06 | -0.69016 | 0.647 | 0.819 | 0.058078 |
| GADD45A | 4.07E-06 | -0.88558 | 0.265 | 0.649 | 0.058416 |
| AC007906.2 | 4.07E-06 | 0.882178 | 0.647 | 0.324 | 0.058463 |
| GNG5 | 4.10E-06 | -0.41473 | 0.912 | 0.957 | 0.05888 |
| RPA3 | 4.10E-06 | 0.485246 | 0.794 | 0.511 | 0.05894 |
| RHOB | 4.12E-06 | -0.71764 | 0.735 | 0.851 | 0.059225 |
| NUCKS1 | 4.13E-06 | 0.387443 | 0.941 | 0.761 | 0.059334 |
| SRPX2 | 4.22E-06 | -0.43848 | 0.294 | 0.654 | 0.060691 |
| GIPC1 | 4.23E-06 | -0.50167 | 0.618 | 0.787 | 0.060778 |
| CAMK1D | 4.31E-06 | 0.371272 | 0.824 | 0.516 | 0.061905 |
| CLCF1 | 4.42E-06 | -0.47737 | 0 | 0.426 | 0.063475 |
| SERPINB1 | 4.44E-06 | -0.83258 | 0.971 | 0.915 | 0.06378 |
| AREG | 4.48E-06 | -0.67124 | 0.088 | 0.511 | 0.064341 |
| MT-ND3 | 4.52E-06 | 0.865786 | 1 | 1 | 0.064991 |
| CD47 | 4.74E-06 | -0.58988 | 0.706 | 0.84 | 0.068115 |
| SAA2 | 4.79E-06 | 0.945073 | 0.618 | 0.261 | 0.068803 |
| CLEC2B | 4.84E-06 | -0.79315 | 0.147 | 0.559 | 0.069532 |
| RPL32 | 4.85E-06 | -0.53408 | 1 | 0.973 | 0.069649 |
| SPPL2B | 4.88E-06 | 0.250452 | 0.735 | 0.383 | 0.070165 |
| RNF7 | 4.89E-06 | -0.44645 | 0.853 | 0.883 | 0.070224 |
| TFF3 | 4.93E-06 | 0.597184 | 0.941 | 0.654 | 0.07084 |
| NME7 | 4.95E-06 | 0.287686 | 0.676 | 0.324 | 0.071125 |
| IRS2 | 4.97E-06 | -0.46057 | 0.176 | 0.628 | 0.071411 |
| EEF1A1 | 4.98E-06 | -0.40554 | 1 | 0.995 | 0.071613 |
| MYL12A | 5.04E-06 | -0.82715 | 0.912 | 0.947 | 0.072453 |
| SH2D3A | 5.06E-06 | -0.27159 | 0.088 | 0.511 | 0.072668 |
| CBLC | 5.35E-06 | -0.26391 | 0 | 0.42 | 0.076904 |
| TRIM7 | 5.35E-06 | -0.35206 | 0 | 0.42 | 0.076904 |
| NPHP1 | 5.40E-06 | 0.419679 | 0.559 | 0.213 | 0.077666 |
| S100P | 5.56E-06 | -0.96986 | 0.706 | 0.888 | 0.079828 |
| TAX1BP3 | 5.59E-06 | -0.34869 | 0.294 | 0.633 | 0.080386 |
| HSPB1 | 5.68E-06 | -0.96221 | 0.912 | 0.915 | 0.081652 |
| CEBPZOS | 5.69E-06 | 0.251239 | 0.765 | 0.378 | 0.081704 |
| HEBP2 | 5.71E-06 | -0.56438 | 0.824 | 0.867 | 0.082058 |
| LINC02166 | 5.75E-06 | 0.26608 | 0.559 | 0.181 | 0.082666 |
| MGST3 | 5.78E-06 | 0.397251 | 0.912 | 0.691 | 0.083008 |
| RFX2 | 5.88E-06 | 0.550335 | 0.676 | 0.367 | 0.084433 |
| C21orf2 | 5.97E-06 | 0.326097 | 0.676 | 0.367 | 0.08574 |
| TMEM54 | 6.14E-06 | -0.39114 | 0.206 | 0.644 | 0.088274 |
| LXN | 6.18E-06 | 0.30201 | 0.676 | 0.298 | 0.088864 |
| RUVBL1 | 6.22E-06 | 0.546669 | 0.647 | 0.351 | 0.089438 |
| MDM4 | 6.30E-06 | 0.387491 | 0.735 | 0.362 | 0.090465 |
| POLD4 | 6.32E-06 | -0.51227 | 0.471 | 0.755 | 0.090798 |
| VEZF1 | 6.49E-06 | 0.343214 | 0.824 | 0.574 | 0.093191 |
| RHOA | 6.54E-06 | -0.38579 | 0.912 | 0.941 | 0.094033 |
| DNALI1 | 6.68E-06 | 0.805312 | 0.706 | 0.394 | 0.095961 |
| CLCN3 | 6.75E-06 | 0.334033 | 0.765 | 0.426 | 0.097006 |
| ADIRF | 6.90E-06 | -0.81262 | 0.265 | 0.67 | 0.09909 |
| EXOSC10 | 6.90E-06 | 0.308435 | 0.765 | 0.41 | 0.099121 |
| MAP7 | 6.90E-06 | 0.343293 | 0.824 | 0.548 | 0.099143 |
| RPS13 | 6.93E-06 | -0.56987 | 0.941 | 0.989 | 0.09957 |
| PLEKHA5 | 7.08E-06 | 0.451408 | 0.853 | 0.66 | 0.101756 |
| ODF3B | 7.12E-06 | 0.913041 | 0.882 | 0.745 | 0.102366 |
| TSPAN6 | 7.13E-06 | 0.490118 | 0.794 | 0.665 | 0.102404 |
| CXCL16 | 7.18E-06 | -0.44328 | 0.618 | 0.803 | 0.103225 |
| GON7 | 7.21E-06 | 0.510304 | 0.706 | 0.372 | 0.103596 |
| TPI1 | 7.29E-06 | -0.47885 | 0.912 | 0.936 | 0.104813 |
| RPL27 | 7.31E-06 | -0.49651 | 0.882 | 0.957 | 0.104978 |
| P4HTM | 7.33E-06 | 0.667984 | 0.765 | 0.601 | 0.105267 |
| PRR34-AS1 | 7.37E-06 | 0.279445 | 0.647 | 0.309 | 0.105932 |
| PCED1A | 7.38E-06 | 0.25132 | 0.529 | 0.202 | 0.106028 |
| ANAPC5 | 7.75E-06 | 0.348637 | 0.824 | 0.537 | 0.111434 |
| KTN1 | 7.81E-06 | 0.593273 | 0.941 | 0.931 | 0.112182 |
| MDFI | 7.82E-06 | -0.32445 | 0 | 0.41 | 0.112358 |
| SPHK1 | 7.82E-06 | -0.35602 | 0 | 0.41 | 0.112358 |
| NAT1 | 7.97E-06 | 0.323401 | 0.618 | 0.293 | 0.114586 |
| DPY30 | 8.19E-06 | 0.521405 | 0.853 | 0.691 | 0.117749 |
| TMPRSS4 | 8.21E-06 | -0.49879 | 0.735 | 0.851 | 0.117913 |
| SEC63 | 8.45E-06 | 0.297962 | 0.882 | 0.516 | 0.121476 |
| RPL14 | 8.49E-06 | -0.5131 | 1 | 0.957 | 0.122009 |
| MORN2 | 8.61E-06 | 0.967703 | 0.735 | 0.457 | 0.123664 |
| SFXN3 | 8.79E-06 | 0.2536 | 0.618 | 0.239 | 0.126266 |
| REV3L | 9.04E-06 | 0.252935 | 0.618 | 0.266 | 0.129926 |
| IPO11 | 9.15E-06 | 0.379614 | 0.529 | 0.218 | 0.131422 |
| HIPK1 | 9.16E-06 | 0.408679 | 0.882 | 0.553 | 0.131696 |
| TNFAIP2 | 9.21E-06 | 0.801827 | 0.618 | 0.293 | 0.132301 |
| SEC62 | 9.25E-06 | 0.339102 | 0.971 | 0.894 | 0.132994 |
| ITGA6 | 9.28E-06 | -0.36768 | 0.088 | 0.489 | 0.133383 |
| MACC1 | 9.30E-06 | -0.7656 | 0.529 | 0.787 | 0.133601 |
| COTL1 | 9.43E-06 | -0.36983 | 0 | 0.404 | 0.135497 |
| SOX9 | 9.43E-06 | -0.41868 | 0 | 0.404 | 0.135497 |
| CLIC3 | 9.43E-06 | -0.50397 | 0 | 0.404 | 0.135497 |
| HLA-DPA1 | 9.54E-06 | 0.816826 | 0.912 | 0.734 | 0.137048 |
| TCEAL3 | 9.71E-06 | 0.318796 | 0.765 | 0.463 | 0.139562 |
| TXNDC17 | 9.81E-06 | -0.45823 | 0.824 | 0.862 | 0.141032 |
| PFN2 | 1.01E-05 | 0.358016 | 0.853 | 0.638 | 0.144844 |
| TMEM43 | 1.03E-05 | -0.32382 | 0.147 | 0.569 | 0.147427 |
| CCP110 | 1.04E-05 | 0.416627 | 0.647 | 0.314 | 0.149303 |
| PLA2G10 | 1.05E-05 | 0.294906 | 0.794 | 0.441 | 0.150653 |
| NFX1 | 1.05E-05 | 0.395452 | 0.853 | 0.527 | 0.150814 |
| RPRD2 | 1.05E-05 | 0.267737 | 0.735 | 0.399 | 0.150901 |
| TMEM165 | 1.06E-05 | -0.52294 | 0.824 | 0.862 | 0.152863 |
| SLC25A3 | 1.08E-05 | -0.47839 | 0.971 | 0.91 | 0.155689 |
| RPL29 | 1.09E-05 | -0.49891 | 0.971 | 0.963 | 0.156457 |
| GPCPD1 | 1.11E-05 | -0.41722 | 0.294 | 0.638 | 0.159957 |
| KIF2A | 1.12E-05 | 0.298826 | 0.765 | 0.447 | 0.160681 |
| MUC1 | 1.13E-05 | -0.62802 | 0.853 | 0.926 | 0.162987 |
| ABLIM3 | 1.14E-05 | -0.33564 | 0 | 0.399 | 0.163151 |
| ATF3 | 1.14E-05 | -0.92901 | 0.412 | 0.729 | 0.164152 |
| CFAP298 | 1.17E-05 | 0.608052 | 0.824 | 0.617 | 0.168185 |
| RPLP1 | 1.20E-05 | -0.48166 | 0.971 | 0.995 | 0.171849 |
| MAP7D1 | 1.22E-05 | -0.37511 | 0.147 | 0.548 | 0.174635 |
| RUVBL2 | 1.22E-05 | 0.638435 | 0.794 | 0.612 | 0.17486 |
| OVCA2 | 1.23E-05 | -0.30268 | 0.088 | 0.511 | 0.177468 |
| EPHX1 | 1.27E-05 | 0.598898 | 0.882 | 0.734 | 0.182779 |
| VPS37B | 1.28E-05 | -0.43042 | 0.559 | 0.793 | 0.184005 |
| PDLIM2 | 1.28E-05 | -0.45173 | 0.235 | 0.633 | 0.18461 |
| RPL10 | 1.30E-05 | -0.45767 | 0.971 | 0.984 | 0.1861 |
| MRPS33 | 1.31E-05 | 0.415959 | 0.882 | 0.612 | 0.188171 |
| UBAC1 | 1.33E-05 | 0.37021 | 0.794 | 0.5 | 0.191336 |
| LPGAT1 | 1.33E-05 | 0.316136 | 0.735 | 0.362 | 0.191738 |
| UPP1 | 1.37E-05 | -0.48079 | 0 | 0.394 | 0.196155 |
| SERPINB4 | 1.37E-05 | -0.6705 | 0 | 0.394 | 0.196155 |
| TSPAN14 | 1.38E-05 | -0.40341 | 0.441 | 0.734 | 0.198433 |
| IFT172 | 1.41E-05 | 0.399441 | 0.824 | 0.574 | 0.202429 |
| NME2 | 1.42E-05 | -0.62227 | 0.853 | 0.883 | 0.204594 |
| STX16 | 1.45E-05 | 0.291598 | 0.765 | 0.441 | 0.208188 |
| NAP1L4 | 1.45E-05 | 0.2878 | 0.882 | 0.628 | 0.208446 |
| TSC22D3 | 1.45E-05 | -0.52939 | 0.353 | 0.686 | 0.208982 |
| KIAA0556 | 1.47E-05 | 0.253925 | 0.647 | 0.298 | 0.211031 |
| SECISBP2L | 1.48E-05 | 0.348851 | 0.824 | 0.628 | 0.213125 |
| TNFRSF21 | 1.49E-05 | -0.48144 | 0.676 | 0.809 | 0.213551 |
| FAM206A | 1.49E-05 | 0.303023 | 0.676 | 0.319 | 0.213671 |
| WDR19 | 1.52E-05 | 0.302988 | 0.5 | 0.181 | 0.218824 |
| SLC6A14 | 1.54E-05 | -0.68941 | 0.471 | 0.803 | 0.221363 |
| ATP6V0E1 | 1.54E-05 | -0.45227 | 0.882 | 0.952 | 0.222001 |
| AL451165.2 | 1.56E-05 | 0.256097 | 0.647 | 0.303 | 0.224199 |
| ARHGAP18 | 1.57E-05 | 0.742586 | 0.765 | 0.638 | 0.224891 |
| BHLHE40 | 1.57E-05 | -0.52955 | 0.382 | 0.691 | 0.225622 |
| CAPS | 1.58E-05 | 1.270259 | 0.765 | 0.633 | 0.22726 |
| B4GALT5 | 1.59E-05 | -0.5052 | 0.706 | 0.851 | 0.229167 |
| RABL2B | 1.61E-05 | 0.545261 | 0.647 | 0.34 | 0.231315 |
| HIST1H2BK | 1.63E-05 | -0.43783 | 0.118 | 0.484 | 0.233581 |
| ZSWIM4 | 1.64E-05 | -0.27866 | 0 | 0.388 | 0.235483 |
| TAX1BP1 | 1.66E-05 | 0.440031 | 0.912 | 0.894 | 0.238081 |
| SEMA3C | 1.67E-05 | 0.291267 | 0.824 | 0.479 | 0.239595 |
| PGD | 1.67E-05 | -0.52544 | 0.676 | 0.787 | 0.240612 |
| SLC25A29 | 1.69E-05 | 0.317547 | 0.765 | 0.473 | 0.242309 |
| TMEM245 | 1.71E-05 | 0.29764 | 0.706 | 0.383 | 0.246135 |
| DMKN | 1.73E-05 | 0.631492 | 0.735 | 0.537 | 0.248213 |
| BCLAF1 | 1.79E-05 | 0.379173 | 0.912 | 0.707 | 0.257824 |
| GPX2 | 1.85E-05 | -0.80224 | 0.235 | 0.596 | 0.265824 |
| CLDN1 | 1.86E-05 | -0.6477 | 0.441 | 0.75 | 0.267161 |
| CEACAM1 | 1.90E-05 | -0.48872 | 0.118 | 0.505 | 0.273717 |
| MT-ND1 | 1.91E-05 | 0.620185 | 1 | 1 | 0.274254 |
| DMAC1 | 1.93E-05 | 0.270705 | 0.824 | 0.569 | 0.277868 |
| BDH1 | 1.95E-05 | 0.571635 | 0.647 | 0.394 | 0.280287 |
| GNA15 | 1.96E-05 | -0.35338 | 0.324 | 0.66 | 0.281622 |
| JUND | 1.97E-05 | -0.56398 | 0.735 | 0.91 | 0.28266 |
| DPM3 | 1.98E-05 | 0.329441 | 0.882 | 0.691 | 0.284795 |
| HSPH1 | 2.03E-05 | 0.859728 | 0.853 | 0.729 | 0.291418 |
| ROCK1 | 2.04E-05 | 0.303867 | 0.853 | 0.532 | 0.292658 |
| ZNF664 | 2.04E-05 | 0.375247 | 0.794 | 0.574 | 0.293178 |
| METRNL | 2.07E-05 | -0.37745 | 0.088 | 0.463 | 0.297247 |
| YBX1 | 2.09E-05 | -0.61931 | 0.912 | 0.91 | 0.300237 |
| NFKBIA | 2.11E-05 | -0.65955 | 0.412 | 0.734 | 0.303689 |
| FDXR | 2.14E-05 | 0.252784 | 0.588 | 0.255 | 0.307457 |
| NABP1 | 2.14E-05 | -0.43271 | 0.118 | 0.495 | 0.307683 |
| LRP11 | 2.15E-05 | 0.619697 | 0.794 | 0.606 | 0.308237 |
| PCBP1 | 2.18E-05 | -0.42248 | 0.912 | 0.936 | 0.31339 |
| PRR7 | 2.21E-05 | 0.331963 | 0.588 | 0.255 | 0.317364 |
| MT1F | 2.23E-05 | 0.352054 | 0.618 | 0.282 | 0.320539 |
| AUTS2 | 2.23E-05 | 0.388674 | 0.824 | 0.543 | 0.320621 |
| SPINT1 | 2.23E-05 | -0.47654 | 0.706 | 0.83 | 0.320651 |
| DCAF7 | 2.24E-05 | 0.264543 | 0.882 | 0.527 | 0.32157 |
| XAB2 | 2.30E-05 | 0.279406 | 0.735 | 0.41 | 0.330247 |
| ALPL | 2.30E-05 | -0.53619 | 0.265 | 0.665 | 0.330261 |
| UCP2 | 2.32E-05 | 0.354291 | 0.912 | 0.734 | 0.333322 |
| AC005392.2 | 2.35E-05 | -0.40573 | 0 | 0.378 | 0.33788 |
| PLAU | 2.35E-05 | -0.68226 | 0 | 0.378 | 0.33788 |
| CCL2 | 2.35E-05 | -1.62041 | 0 | 0.378 | 0.33788 |
| IFITM2 | 2.38E-05 | -0.66574 | 0.735 | 0.84 | 0.34204 |
| EPS8L2 | 2.38E-05 | -0.56124 | 0.647 | 0.803 | 0.342494 |
| ARL3 | 2.44E-05 | 0.503508 | 0.735 | 0.489 | 0.350304 |
| ST5 | 2.48E-05 | 0.320546 | 0.824 | 0.574 | 0.355865 |
| SPAG16 | 2.50E-05 | 0.576416 | 0.706 | 0.468 | 0.358859 |
| SLC38A2 | 2.50E-05 | -0.44758 | 0.529 | 0.777 | 0.359856 |
| SHROOM3 | 2.50E-05 | 0.28391 | 0.706 | 0.378 | 0.359872 |
| MALAT1 | 2.50E-05 | 0.503233 | 0.971 | 1 | 0.359884 |
| ALS2CL | 2.56E-05 | -0.43132 | 0.294 | 0.628 | 0.368569 |
| RPL9 | 2.69E-05 | -0.36014 | 0.971 | 0.963 | 0.386037 |
| LUC7L3 | 2.72E-05 | 0.367165 | 0.882 | 0.612 | 0.391255 |
| MLEC | 2.77E-05 | 0.331334 | 0.912 | 0.617 | 0.398145 |
| CLCA4 | 2.81E-05 | -0.47505 | 0 | 0.372 | 0.403848 |
| SPRR3 | 2.81E-05 | -0.66098 | 0 | 0.372 | 0.403848 |
| SPON2 | 2.84E-05 | -0.443 | 0.118 | 0.473 | 0.407771 |
| UBA52 | 2.86E-05 | -0.35248 | 0.971 | 0.973 | 0.411634 |
| MAGED2 | 2.89E-05 | 0.355635 | 0.882 | 0.691 | 0.415507 |
| CCNL1 | 2.95E-05 | -0.51472 | 0.647 | 0.835 | 0.423764 |
| ZNF688 | 2.96E-05 | 0.368539 | 0.588 | 0.303 | 0.425786 |
| RB1CC1 | 2.96E-05 | 0.404868 | 0.853 | 0.686 | 0.4259 |
| EPRS | 2.99E-05 | 0.273522 | 0.824 | 0.543 | 0.429768 |
| CASZ1 | 2.99E-05 | 0.471529 | 0.735 | 0.505 | 0.430298 |
| MGMT | 3.02E-05 | 0.478479 | 0.765 | 0.527 | 0.43454 |
| RPL22L1 | 3.04E-05 | -0.57623 | 0.676 | 0.798 | 0.43744 |
| AQR | 3.06E-05 | 0.259053 | 0.559 | 0.239 | 0.439871 |
| ABCA5 | 3.08E-05 | 0.323684 | 0.647 | 0.356 | 0.443042 |
| SELENOK | 3.10E-05 | -0.40443 | 0.882 | 0.846 | 0.445232 |
| SCPEP1 | 3.11E-05 | 0.423143 | 0.794 | 0.537 | 0.446922 |
| RABL6 | 3.11E-05 | 0.264706 | 0.824 | 0.543 | 0.447141 |
| CFAP20 | 3.13E-05 | 0.277176 | 0.647 | 0.303 | 0.449194 |
| RPL15 | 3.19E-05 | -0.3979 | 0.941 | 0.979 | 0.458698 |
| MPC2 | 3.20E-05 | 0.338583 | 0.853 | 0.686 | 0.460117 |
| NIPBL | 3.21E-05 | 0.39974 | 0.853 | 0.606 | 0.460769 |
| SF3B2 | 3.24E-05 | 0.43029 | 0.941 | 0.803 | 0.465315 |
| GADD45GIP1 | 3.26E-05 | 0.337964 | 0.941 | 0.766 | 0.468307 |
| FLNB | 3.32E-05 | 0.383983 | 0.941 | 0.771 | 0.476782 |
| MTG2 | 3.32E-05 | 0.290478 | 0.647 | 0.266 | 0.477194 |
| SPTSSB | 3.35E-05 | -0.25674 | 0 | 0.367 | 0.482005 |
| AL121761.2 | 3.35E-05 | -0.31963 | 0 | 0.367 | 0.482005 |
| TMPRSS11D | 3.35E-05 | -0.38086 | 0 | 0.367 | 0.482005 |
| TIMP2 | 3.35E-05 | -0.43325 | 0 | 0.367 | 0.482005 |
| TPR | 3.36E-05 | 0.333954 | 0.824 | 0.628 | 0.482869 |
| TUFT1 | 3.36E-05 | -0.45326 | 0.118 | 0.473 | 0.48347 |
| FBXO32 | 3.37E-05 | -0.39025 | 0.147 | 0.489 | 0.483646 |
| IFT57 | 3.40E-05 | 0.792458 | 0.765 | 0.612 | 0.488046 |
| CXCL1 | 3.46E-05 | -0.87997 | 0.324 | 0.676 | 0.496978 |
| RPL37A | 3.49E-05 | -0.41576 | 0.971 | 0.989 | 0.501329 |
| PBRM1 | 3.50E-05 | 0.284401 | 0.765 | 0.505 | 0.502536 |
| SPAG1 | 3.51E-05 | 0.56537 | 0.735 | 0.58 | 0.503737 |
| ZFP36L2 | 3.53E-05 | -0.62521 | 0.765 | 0.851 | 0.507457 |
| TES | 3.59E-05 | -0.49149 | 0.588 | 0.766 | 0.515778 |
| ZFHX3 | 3.62E-05 | 0.340481 | 0.824 | 0.479 | 0.519632 |
| WRB | 3.62E-05 | 0.357726 | 0.618 | 0.309 | 0.520841 |
| HIST1H2AC | 3.67E-05 | -0.73949 | 0.441 | 0.718 | 0.527517 |
| RPL30 | 3.72E-05 | -0.45126 | 0.971 | 0.979 | 0.533954 |
| LAYN | 3.78E-05 | -0.43956 | 0.147 | 0.505 | 0.542899 |
| COLCA1 | 3.80E-05 | 0.259452 | 0.676 | 0.309 | 0.545463 |
| KMT2A | 3.82E-05 | 0.300473 | 0.824 | 0.527 | 0.549447 |
| ICE2 | 3.85E-05 | 0.300625 | 0.588 | 0.239 | 0.553478 |
| KIF3B | 3.89E-05 | 0.41175 | 0.765 | 0.479 | 0.558828 |
| PKM | 3.90E-05 | -0.38944 | 0.941 | 0.957 | 0.561133 |
| SNRNP200 | 3.91E-05 | 0.323058 | 0.794 | 0.473 | 0.562562 |
| SMPD2 | 3.93E-05 | 0.340113 | 0.676 | 0.383 | 0.565069 |
| COQ4 | 3.96E-05 | 0.455901 | 0.853 | 0.644 | 0.569657 |
| GLG1 | 3.98E-05 | 0.25451 | 0.824 | 0.548 | 0.571314 |
| PLCD3 | 4.00E-05 | -0.25253 | 0 | 0.362 | 0.574474 |
| GJB4 | 4.00E-05 | -0.2557 | 0 | 0.362 | 0.574474 |
| TINAGL1 | 4.00E-05 | -0.27214 | 0 | 0.362 | 0.574474 |
| PRDM1 | 4.00E-05 | -0.27426 | 0 | 0.362 | 0.574474 |
| FOXQ1 | 4.00E-05 | -0.27662 | 0 | 0.362 | 0.574474 |
| AL355312.4 | 4.00E-05 | -0.36099 | 0 | 0.362 | 0.574474 |
| GOLGB1 | 4.02E-05 | 0.392699 | 0.912 | 0.766 | 0.577848 |
| MT-CYB | 4.08E-05 | 0.636589 | 0.971 | 0.995 | 0.586819 |
| SMIM22 | 4.11E-05 | 0.555707 | 0.882 | 0.888 | 0.590453 |
| WDR90 | 4.15E-05 | 0.28871 | 0.588 | 0.271 | 0.596421 |
| VSIG2 | 4.21E-05 | -0.53337 | 0.412 | 0.67 | 0.605339 |
| CARS | 4.29E-05 | 0.72429 | 0.676 | 0.42 | 0.615954 |
| TMEM63A | 4.31E-05 | 0.306689 | 0.618 | 0.287 | 0.619888 |
| DRAM2 | 4.35E-05 | 0.307517 | 0.706 | 0.399 | 0.625632 |
| FMO5 | 4.39E-05 | 0.255655 | 0.529 | 0.229 | 0.631442 |
| ERGIC3 | 4.41E-05 | 0.46327 | 0.941 | 0.835 | 0.634093 |
| SAT1 | 4.49E-05 | -0.45168 | 1 | 1 | 0.644568 |
| TNKS1BP1 | 4.53E-05 | -0.27898 | 0.176 | 0.564 | 0.650438 |
| PLP2 | 4.58E-05 | -0.58668 | 0.471 | 0.702 | 0.658756 |
| DNAL4 | 4.60E-05 | 0.253418 | 0.618 | 0.293 | 0.661624 |
| FTSJ1 | 4.61E-05 | -0.26337 | 0.176 | 0.527 | 0.662113 |
| SCAMP4 | 4.68E-05 | 0.331065 | 0.735 | 0.463 | 0.672434 |
| AC007255.1 | 4.70E-05 | 0.27492 | 0.588 | 0.261 | 0.675149 |
| TMEM106C | 4.76E-05 | -0.26898 | 0 | 0.356 | 0.683722 |
| FSTL3 | 4.76E-05 | -0.43485 | 0 | 0.356 | 0.683722 |
| CHCHD6 | 4.80E-05 | 0.257425 | 0.529 | 0.223 | 0.690194 |
| N4BP2L2 | 4.95E-05 | 0.368742 | 0.912 | 0.649 | 0.710727 |
| AMOTL2 | 4.96E-05 | -0.336 | 0.176 | 0.553 | 0.713338 |
| DEFB1 | 5.05E-05 | -0.59098 | 0.235 | 0.596 | 0.725736 |
| EID1 | 5.14E-05 | 0.421801 | 0.941 | 0.798 | 0.738313 |
| B9D2 | 5.15E-05 | 0.301338 | 0.618 | 0.314 | 0.739946 |
| LRP10 | 5.27E-05 | -0.40164 | 0.706 | 0.862 | 0.757897 |
| AQP5 | 5.31E-05 | -1.33346 | 0.735 | 0.777 | 0.76265 |
| RSAD2 | 5.32E-05 | -0.92181 | 0.765 | 0.867 | 0.764348 |
| IFT22 | 5.41E-05 | 0.413625 | 0.765 | 0.452 | 0.777999 |
| GABARAPL1 | 5.44E-05 | -0.39987 | 0.294 | 0.574 | 0.782055 |
| OFD1 | 5.53E-05 | 0.324034 | 0.765 | 0.5 | 0.795022 |
| SOX4 | 5.56E-05 | -0.79477 | 0.647 | 0.84 | 0.798912 |
| LRBA | 5.58E-05 | 0.306623 | 0.647 | 0.324 | 0.801529 |
| KRT18 | 5.61E-05 | -0.64064 | 0.971 | 0.968 | 0.805721 |
| FOSL2 | 5.61E-05 | -0.34274 | 0.294 | 0.66 | 0.80663 |
| MIR222HG | 5.65E-05 | -0.27735 | 0 | 0.351 | 0.812619 |
| NDRG1 | 5.65E-05 | -0.34784 | 0 | 0.351 | 0.812619 |
| LIF | 5.65E-05 | -0.36283 | 0 | 0.351 | 0.812619 |
| TFPI2 | 5.65E-05 | -0.53857 | 0 | 0.351 | 0.812619 |
| RPS18 | 5.78E-05 | -0.51007 | 0.971 | 0.973 | 0.831027 |
| MECP2 | 5.88E-05 | 0.334362 | 0.824 | 0.479 | 0.845607 |
| PIM1 | 5.89E-05 | -0.44093 | 0.206 | 0.58 | 0.846417 |
| MTSS1 | 6.01E-05 | 0.34077 | 0.618 | 0.324 | 0.863424 |
| ALDOA | 6.02E-05 | -0.55966 | 0.941 | 0.867 | 0.865159 |
| UBE2D3 | 6.10E-05 | -0.39246 | 0.912 | 0.936 | 0.876185 |
| 7-Sep | 6.13E-05 | 0.289717 | 0.912 | 0.713 | 0.881323 |
| SMIM14 | 6.17E-05 | 0.390227 | 1 | 0.824 | 0.886411 |
| ANXA2 | 6.19E-05 | -0.43803 | 1 | 0.979 | 0.889285 |
| SH3BGRL | 6.19E-05 | 0.279292 | 0.824 | 0.601 | 0.889993 |
| UFC1 | 6.21E-05 | 0.665713 | 0.882 | 0.899 | 0.891881 |
| C22orf39 | 6.25E-05 | 0.270241 | 0.824 | 0.596 | 0.897994 |
| NFIX | 6.28E-05 | 0.429321 | 0.765 | 0.436 | 0.9028 |
| RXRA | 6.39E-05 | 0.44998 | 0.794 | 0.468 | 0.917857 |
| PIGR | 6.40E-05 | 0.645536 | 0.971 | 0.83 | 0.919691 |
| ECPAS | 6.46E-05 | 0.323918 | 0.765 | 0.457 | 0.928724 |
| C16orf89 | 6.55E-05 | 0.311183 | 0.529 | 0.218 | 0.940534 |
| F2RL1 | 6.63E-05 | -0.45129 | 0.294 | 0.585 | 0.952823 |
| MAPK13 | 6.75E-05 | -0.2621 | 0.147 | 0.495 | 0.970317 |
| BAIAP2L1 | 6.80E-05 | 0.487122 | 0.824 | 0.707 | 0.977124 |
| GSTP1 | 6.82E-05 | -0.45742 | 0.971 | 0.968 | 0.980591 |
| U2AF2 | 6.83E-05 | 0.264928 | 0.882 | 0.548 | 0.981478 |
| MPZL2 | 6.85E-05 | -0.4014 | 0.559 | 0.745 | 0.983627 |
| C19orf70 | 7.04E-05 | 0.367294 | 0.912 | 0.803 | 1 |
| ATP6V0C | 7.09E-05 | -0.47065 | 0.765 | 0.878 | 1 |
| AARS | 7.25E-05 | 0.270441 | 0.706 | 0.388 | 1 |
| LDHA | 7.29E-05 | -0.57797 | 0.588 | 0.755 | 1 |
| WASHC2A | 7.30E-05 | 0.267261 | 0.647 | 0.346 | 1 |
| PRSS12 | 7.37E-05 | 0.264394 | 0.618 | 0.303 | 1 |
| PPIC | 7.38E-05 | -0.40052 | 0.588 | 0.755 | 1 |
| NUDT4 | 7.49E-05 | 0.471804 | 0.735 | 0.548 | 1 |
| STRBP | 7.51E-05 | 0.299009 | 0.765 | 0.484 | 1 |
| FAM3C | 7.72E-05 | -0.4751 | 0.765 | 0.851 | 1 |
| HNRNPA2B1 | 7.89E-05 | 0.28037 | 1 | 0.947 | 1 |
| FEM1C | 7.95E-05 | -0.2808 | 0.176 | 0.543 | 1 |
| CREB3L1 | 7.99E-05 | -0.43248 | 0.235 | 0.67 | 1 |
| KLF2 | 8.07E-05 | -0.66558 | 0.412 | 0.617 | 1 |
| ALDH3B1 | 8.18E-05 | 0.723087 | 0.706 | 0.628 | 1 |
| LARP1 | 8.20E-05 | 0.264848 | 0.912 | 0.622 | 1 |
| SIVA1 | 8.21E-05 | 0.264675 | 0.882 | 0.665 | 1 |
| B3GNT3 | 8.24E-05 | -0.30106 | 0.235 | 0.601 | 1 |
| RHOC | 8.31E-05 | -0.42853 | 0.588 | 0.761 | 1 |
| TRIM13 | 8.33E-05 | 0.303341 | 0.706 | 0.404 | 1 |
| PRKAR2A | 8.42E-05 | 0.262048 | 0.765 | 0.463 | 1 |
| FAM174A | 8.59E-05 | 0.599064 | 0.765 | 0.617 | 1 |
| 6-Mar | 8.66E-05 | 0.374653 | 0.735 | 0.537 | 1 |
| TSTD1 | 8.79E-05 | 0.474948 | 0.853 | 0.83 | 1 |
| MCUB | 8.88E-05 | -0.41635 | 0.294 | 0.644 | 1 |
| IARS2 | 8.89E-05 | 0.345986 | 0.735 | 0.5 | 1 |
| FUT2 | 8.94E-05 | -0.41102 | 0.382 | 0.67 | 1 |
| ZNF638 | 9.14E-05 | 0.264472 | 0.794 | 0.532 | 1 |
| CDC42EP5 | 9.16E-05 | -0.31618 | 0.235 | 0.569 | 1 |
| CIB1 | 9.20E-05 | 0.582749 | 0.941 | 0.915 | 1 |
| REC8 | 9.40E-05 | 0.260445 | 0.824 | 0.532 | 1 |
| PHLDA1 | 9.42E-05 | -0.37119 | 0 | 0.335 | 1 |
| EDN1 | 9.42E-05 | -0.50734 | 0 | 0.335 | 1 |
| TUBB | 9.84E-05 | -0.49356 | 0.529 | 0.734 | 1 |
| BIK | 9.89E-05 | -0.47523 | 0.559 | 0.782 | 1 |
| PSMA7 | 9.93E-05 | -0.37641 | 0.912 | 0.91 | 1 |
| KCNK1 | 9.95E-05 | -0.35405 | 0.294 | 0.574 | 1 |
| ADRB2 | 9.96E-05 | -0.32629 | 0.118 | 0.463 | 1 |
| RTF1 | 0.000102 | 0.258957 | 0.853 | 0.564 | 1 |
| MGLL | 0.000102 | 0.475071 | 0.676 | 0.42 | 1 |
| PSD3 | 0.000103 | 0.284169 | 0.588 | 0.314 | 1 |
| DDX46 | 0.000104 | 0.298073 | 0.824 | 0.574 | 1 |
| RNF24 | 0.000104 | -0.25186 | 0.176 | 0.495 | 1 |
| TMC4 | 0.000104 | 0.449198 | 0.824 | 0.723 | 1 |
| SIX1 | 0.000105 | 0.296222 | 0.765 | 0.511 | 1 |
| LGALS7B | 0.000105 | -0.65435 | 0.088 | 0.42 | 1 |
| ZNF609 | 0.000106 | 0.307679 | 0.676 | 0.436 | 1 |
| FAM49B | 0.000107 | -0.33958 | 0.471 | 0.67 | 1 |
| SLC3A2 | 0.000108 | -0.71542 | 0.529 | 0.761 | 1 |
| MBTPS1 | 0.000109 | 0.273126 | 0.794 | 0.532 | 1 |
| EIF4G3 | 0.000109 | 0.341609 | 0.735 | 0.479 | 1 |
| TNS4 | 0.000111 | -0.26422 | 0 | 0.33 | 1 |
| HPCAL1 | 0.000111 | -0.27425 | 0 | 0.33 | 1 |
| HBEGF | 0.000111 | -0.34966 | 0 | 0.33 | 1 |
| LGALS7 | 0.000111 | -0.44229 | 0 | 0.33 | 1 |
| MYL9 | 0.000111 | -0.60441 | 0 | 0.33 | 1 |
| BASP1 | 0.000113 | 0.606407 | 0.647 | 0.415 | 1 |
| RPL36 | 0.000113 | -0.33431 | 1 | 0.989 | 1 |
| TMOD3 | 0.000114 | -0.40257 | 0.794 | 0.851 | 1 |
| CX3CL1 | 0.000114 | 0.312099 | 0.676 | 0.372 | 1 |
| ITGB8 | 0.000114 | -0.83393 | 0.618 | 0.777 | 1 |
| AGPAT2 | 0.000116 | -0.47362 | 0.618 | 0.718 | 1 |
| VPS35 | 0.00012 | 0.254793 | 0.882 | 0.697 | 1 |
| APH1A | 0.000121 | -0.35635 | 0.412 | 0.681 | 1 |
| FUT6 | 0.000122 | -0.34671 | 0.235 | 0.537 | 1 |
| MRPL14 | 0.000123 | -0.34502 | 0.882 | 0.846 | 1 |
| PNISR | 0.000126 | 0.292467 | 0.853 | 0.691 | 1 |
| PEA15 | 0.000126 | -0.3461 | 0.294 | 0.628 | 1 |
| KRT10 | 0.000127 | -0.39189 | 0.912 | 0.904 | 1 |
| EIF1 | 0.000127 | -0.35292 | 0.971 | 0.979 | 1 |
| CKAP4 | 0.000129 | -0.34756 | 0.471 | 0.739 | 1 |
| PARVA | 0.00013 | 0.312927 | 0.794 | 0.537 | 1 |
| TKT | 0.00013 | -0.4759 | 0.618 | 0.766 | 1 |
| RPS2 | 0.00013 | -0.54761 | 0.971 | 0.968 | 1 |
| TIMP1 | 0.000131 | -0.80305 | 0.735 | 0.867 | 1 |
| MYEOV | 0.000131 | -0.28053 | 0 | 0.324 | 1 |
| TCIM | 0.000131 | -0.38261 | 0 | 0.324 | 1 |
| CXCL2 | 0.000131 | -0.50699 | 0 | 0.324 | 1 |
| SERPINB8 | 0.000131 | -0.2603 | 0.147 | 0.468 | 1 |
| AC015912.3 | 0.000132 | -0.43767 | 0.235 | 0.548 | 1 |
| CASC3 | 0.000133 | 0.30382 | 0.735 | 0.415 | 1 |
| WBP1 | 0.000133 | 0.254426 | 0.676 | 0.404 | 1 |
| VSIR | 0.000134 | -0.38943 | 0.559 | 0.75 | 1 |
| MANF | 0.000136 | -0.35255 | 0.5 | 0.713 | 1 |
| BRWD1 | 0.000137 | 0.294457 | 0.735 | 0.394 | 1 |
| RPS6KA3 | 0.000139 | -0.41778 | 0.294 | 0.564 | 1 |
| RPL37 | 0.00014 | -0.39039 | 1 | 0.984 | 1 |
| DENND2C | 0.00014 | -0.39258 | 0.206 | 0.527 | 1 |
| COL4A3BP | 0.000142 | 0.296363 | 0.853 | 0.553 | 1 |
| CD59 | 0.000143 | 0.516176 | 0.912 | 0.883 | 1 |
| RCAN3 | 0.000146 | 0.358164 | 0.676 | 0.42 | 1 |
| GTF3C1 | 0.000148 | 0.258383 | 0.676 | 0.372 | 1 |
| MT-CO3 | 0.000148 | 0.593736 | 1 | 0.995 | 1 |
| GBP2 | 0.000149 | -0.40194 | 0.088 | 0.441 | 1 |
| UBL3 | 0.000149 | 0.272597 | 0.853 | 0.638 | 1 |
| MAN1B1 | 0.000151 | 0.277981 | 0.676 | 0.33 | 1 |
| PTGES | 0.000155 | -0.25974 | 0 | 0.319 | 1 |
| OST4 | 0.000155 | -0.36129 | 0.853 | 0.91 | 1 |
| PDLIM5 | 0.000155 | -0.50745 | 0.794 | 0.84 | 1 |
| MYO5C | 0.000156 | -0.37431 | 0.618 | 0.777 | 1 |
| RARG | 0.000159 | -0.25593 | 0.176 | 0.5 | 1 |
| NTS | 0.000164 | -0.83985 | 0.324 | 0.628 | 1 |
| GLYR1 | 0.000165 | 0.282099 | 0.824 | 0.527 | 1 |
| RPS27A | 0.000166 | -0.35306 | 0.971 | 0.984 | 1 |
| CXCL17 | 0.000167 | -0.52962 | 0.971 | 0.957 | 1 |
| CIR1 | 0.000167 | 0.3632 | 0.824 | 0.67 | 1 |
| HMGB3 | 0.000169 | 0.371713 | 0.824 | 0.553 | 1 |
| PPP1R14C | 0.000169 | 0.285518 | 0.588 | 0.287 | 1 |
| CYB561A3 | 0.000171 | 0.29586 | 0.618 | 0.362 | 1 |
| ANKRD65 | 0.000172 | 0.280878 | 0.676 | 0.42 | 1 |
| MAGED1 | 0.000177 | 0.278579 | 0.824 | 0.436 | 1 |
| AK1 | 0.000178 | 0.372621 | 0.647 | 0.34 | 1 |
| GNB2 | 0.000179 | -0.33039 | 0.941 | 0.952 | 1 |
| SPTLC2 | 0.000182 | 0.301551 | 0.824 | 0.537 | 1 |
| FLNA | 0.000182 | -0.45629 | 0 | 0.314 | 1 |
| ANKLE2 | 0.000183 | -0.38172 | 0.441 | 0.702 | 1 |
| EEF1B2 | 0.000186 | -0.47251 | 0.941 | 0.888 | 1 |
| POLR2J3.1 | 0.000186 | 0.290523 | 0.618 | 0.309 | 1 |
| LY6E | 0.000189 | -0.50985 | 1 | 0.936 | 1 |
| DEGS2 | 0.00019 | 0.529737 | 0.676 | 0.484 | 1 |
| UCHL3 | 0.000193 | -0.3106 | 0.382 | 0.612 | 1 |
| DNAJC10 | 0.000194 | 0.363854 | 0.735 | 0.569 | 1 |
| SCAF11 | 0.000194 | 0.258908 | 0.853 | 0.734 | 1 |
| EMP3 | 0.000195 | -0.57431 | 0.176 | 0.505 | 1 |
| SERTAD1 | 0.000196 | -0.44186 | 0.618 | 0.729 | 1 |
| ANKRD54 | 0.000198 | 0.268648 | 0.647 | 0.372 | 1 |
| NASP | 0.000206 | 0.374555 | 0.706 | 0.447 | 1 |
| NEDD9 | 0.000214 | -0.48241 | 0.176 | 0.479 | 1 |
| CDKN2B | 0.000215 | -0.32351 | 0 | 0.309 | 1 |
| DKK1 | 0.000215 | -0.60492 | 0 | 0.309 | 1 |
| ATP1B3 | 0.000218 | -0.3358 | 0.294 | 0.601 | 1 |
| WDR34 | 0.000222 | 0.409908 | 0.765 | 0.59 | 1 |
| OXSR1 | 0.000228 | -0.26785 | 0.235 | 0.553 | 1 |
| APOBEC3A | 0.00023 | -0.72734 | 0.088 | 0.42 | 1 |
| SSB | 0.000231 | 0.381403 | 0.912 | 0.793 | 1 |
| TGM2 | 0.000233 | 0.638173 | 0.794 | 0.574 | 1 |
| CNN3 | 0.000235 | -0.35175 | 0.618 | 0.83 | 1 |
| GLIPR2 | 0.000236 | 0.300233 | 0.853 | 0.649 | 1 |
| AP3S1 | 0.000237 | -0.27921 | 0.265 | 0.553 | 1 |
| ARF1 | 0.000242 | -0.34217 | 0.853 | 0.888 | 1 |
| MRPL40 | 0.000244 | 0.321783 | 0.882 | 0.718 | 1 |
| MAN1A2 | 0.000246 | 0.33739 | 0.794 | 0.548 | 1 |
| DNPH1 | 0.000252 | 0.538306 | 0.765 | 0.67 | 1 |
| PGAM1 | 0.000253 | -0.3671 | 0.882 | 0.894 | 1 |
| GRAMD2B | 0.000257 | -0.6584 | 0.735 | 0.814 | 1 |
| POR | 0.000263 | 0.283382 | 0.912 | 0.771 | 1 |
| TRIM2 | 0.000272 | 0.272256 | 0.853 | 0.793 | 1 |
| CLU | 0.000273 | 0.559203 | 0.971 | 0.91 | 1 |
| MIR22HG | 0.000275 | -0.30605 | 0.324 | 0.617 | 1 |
| NFKBIZ | 0.000282 | -0.30606 | 0.588 | 0.798 | 1 |
| OAS3 | 0.000283 | -0.43311 | 0.765 | 0.872 | 1 |
| RPS4X | 0.000285 | -0.51283 | 0.941 | 0.963 | 1 |
| RPL11 | 0.000285 | -0.42012 | 1 | 0.957 | 1 |
| MMP7 | 0.000291 | -0.66147 | 0.088 | 0.394 | 1 |
| CTSS | 0.000293 | 0.416159 | 0.853 | 0.862 | 1 |
| CTSB | 0.000293 | -0.36342 | 0.971 | 0.952 | 1 |
| ARID1B | 0.000293 | 0.341101 | 0.824 | 0.564 | 1 |
| KRT6B | 0.000296 | -0.4876 | 0 | 0.298 | 1 |
| KRT16 | 0.000296 | -0.59075 | 0 | 0.298 | 1 |
| PEBP1 | 0.0003 | 0.450616 | 0.853 | 0.739 | 1 |
| NAPRT | 0.000305 | -0.2893 | 0.235 | 0.559 | 1 |
| FDX1 | 0.000306 | -0.36864 | 0.765 | 0.782 | 1 |
| PABPC1 | 0.000315 | -0.35827 | 0.941 | 0.952 | 1 |
| RPL7 | 0.000317 | -0.3717 | 0.971 | 0.941 | 1 |
| DCLRE1C | 0.000318 | -0.31078 | 0.147 | 0.441 | 1 |
| CPD | 0.00032 | 0.269072 | 0.882 | 0.654 | 1 |
| NDUFB1 | 0.000325 | 0.278644 | 0.941 | 0.915 | 1 |
| OCIAD2 | 0.000327 | -0.41686 | 0.676 | 0.803 | 1 |
| RPL17 | 0.000327 | -0.3688 | 0.882 | 0.899 | 1 |
| NBL1 | 0.000331 | -0.36345 | 0.412 | 0.665 | 1 |
| HAGH | 0.000335 | 0.327791 | 0.735 | 0.532 | 1 |
| JPT2 | 0.000337 | 0.391412 | 0.853 | 0.691 | 1 |
| KIFAP3 | 0.000339 | 0.326708 | 0.559 | 0.277 | 1 |
| MT-ND2 | 0.000339 | 0.503392 | 0.971 | 0.995 | 1 |
| POLR2I | 0.000342 | 0.544599 | 0.853 | 0.809 | 1 |
| GPC1 | 0.000343 | -0.28283 | 0.265 | 0.66 | 1 |
| CNDP2 | 0.000344 | 0.27191 | 0.971 | 0.771 | 1 |
| RHOF | 0.000346 | -0.41847 | 0 | 0.293 | 1 |
| RPL34 | 0.000347 | -0.35931 | 0.971 | 0.984 | 1 |
| ERN2 | 0.000347 | 0.368727 | 0.882 | 0.564 | 1 |
| DDX1 | 0.000353 | 0.259534 | 0.735 | 0.463 | 1 |
| USP22 | 0.000354 | 0.345117 | 0.853 | 0.676 | 1 |
| PPP1R13L | 0.000356 | -0.28355 | 0.176 | 0.468 | 1 |
| TAP1 | 0.000362 | -0.45004 | 0.794 | 0.856 | 1 |
| IDO1 | 0.000364 | -0.86098 | 0.235 | 0.553 | 1 |
| PET100 | 0.000375 | 0.271321 | 0.882 | 0.835 | 1 |
| NIPSNAP2 | 0.000377 | 0.268645 | 0.676 | 0.463 | 1 |
| DNAJA4 | 0.00038 | 0.569145 | 0.824 | 0.713 | 1 |
| NAP1L1 | 0.000386 | 0.321714 | 0.853 | 0.777 | 1 |
| NUDC | 0.000388 | 0.556679 | 0.853 | 0.745 | 1 |
| ZFAS1 | 0.000389 | -0.45819 | 0.853 | 0.91 | 1 |
| LRRC59 | 0.000396 | -0.3355 | 0.618 | 0.75 | 1 |
| FAM111A | 0.000396 | 0.321283 | 0.706 | 0.394 | 1 |
| RNF39 | 0.000397 | -0.39255 | 0.147 | 0.447 | 1 |
| ACTN4 | 0.000398 | -0.36312 | 0.971 | 0.936 | 1 |
| ARF5 | 0.000398 | -0.32929 | 0.853 | 0.851 | 1 |
| LIPH | 0.000402 | -0.44453 | 0.471 | 0.745 | 1 |
| MAP3K8 | 0.000407 | -0.3711 | 0.353 | 0.585 | 1 |
| DUSP22 | 0.000408 | 0.263422 | 0.706 | 0.42 | 1 |
| NACA | 0.000412 | -0.36645 | 1 | 0.973 | 1 |
| PNMA1 | 0.000412 | 0.256636 | 0.529 | 0.271 | 1 |
| LNPEP | 0.00042 | 0.293279 | 0.765 | 0.468 | 1 |
| TMEM190 | 0.00042 | 0.549621 | 0.529 | 0.234 | 1 |
| RPS6 | 0.00043 | -0.389 | 0.971 | 0.979 | 1 |
| NAA20 | 0.000431 | 0.303105 | 0.853 | 0.697 | 1 |
| IGFBP2 | 0.000442 | 0.731863 | 0.794 | 0.771 | 1 |
| CIRBP | 0.000443 | 0.291439 | 0.971 | 0.878 | 1 |
| FGGY | 0.000453 | 0.389353 | 0.735 | 0.59 | 1 |
| MLXIP | 0.000457 | 0.261273 | 0.794 | 0.628 | 1 |
| RBPMS | 0.000457 | -0.35177 | 0.5 | 0.681 | 1 |
| KIF9 | 0.00046 | 0.548335 | 0.706 | 0.543 | 1 |
| CD151 | 0.000462 | -0.40832 | 0.647 | 0.819 | 1 |
| WDR1 | 0.000463 | -0.39967 | 0.824 | 0.883 | 1 |
| RPL35A | 0.000467 | -0.36762 | 0.971 | 0.984 | 1 |
| SYF2 | 0.000467 | 0.267726 | 0.853 | 0.617 | 1 |
| ICK | 0.00047 | 0.278351 | 0.676 | 0.394 | 1 |
| GLIPR1 | 0.000474 | -0.31373 | 0 | 0.282 | 1 |
| ADGRF4 | 0.000474 | -0.31501 | 0 | 0.282 | 1 |
| SERPINB13 | 0.000474 | -0.34465 | 0 | 0.282 | 1 |
| RIOK3 | 0.000477 | -0.36904 | 0.618 | 0.75 | 1 |
| RPS15 | 0.00048 | -0.27032 | 1 | 0.973 | 1 |
| TICAM1 | 0.000486 | -0.43516 | 0.353 | 0.574 | 1 |
| AHSA1 | 0.000492 | 0.431208 | 0.824 | 0.676 | 1 |
| ITPRIPL2 | 0.000492 | -0.31577 | 0.235 | 0.527 | 1 |
| SBDS | 0.000494 | -0.3606 | 0.676 | 0.787 | 1 |
| RHPN2 | 0.000494 | 0.292956 | 0.735 | 0.479 | 1 |
| NDUFA3 | 0.000501 | 0.308286 | 0.882 | 0.883 | 1 |
| BTG1 | 0.000506 | -0.58689 | 0.765 | 0.803 | 1 |
| SEC61B | 0.000511 | -0.30104 | 0.941 | 0.926 | 1 |
| PPP2R2D | 0.000514 | 0.300362 | 0.5 | 0.229 | 1 |
| NEAT1 | 0.000515 | -0.64297 | 0.971 | 0.984 | 1 |
| CMTM7 | 0.00053 | -0.31135 | 0.412 | 0.66 | 1 |
| PLCB4 | 0.000531 | 0.2646 | 0.559 | 0.282 | 1 |
| NPIPB15 | 0.000533 | 0.255322 | 0.471 | 0.218 | 1 |
| PHIP | 0.000533 | 0.296861 | 0.853 | 0.718 | 1 |
| HM13 | 0.00054 | -0.31398 | 0.853 | 0.851 | 1 |
| OSMR | 0.000543 | -0.27676 | 0.118 | 0.42 | 1 |
| MNT | 0.000545 | -0.25725 | 0.088 | 0.388 | 1 |
| APOC1 | 0.000554 | 1.662837 | 0.353 | 0.112 | 1 |
| CLIC6 | 0.000554 | 0.303583 | 0.853 | 0.649 | 1 |
| CXCL6 | 0.000554 | -0.6108 | 0.235 | 0.564 | 1 |
| BTF3 | 0.000555 | -0.30666 | 0.971 | 0.952 | 1 |
| CCDC96 | 0.000565 | 0.252728 | 0.441 | 0.181 | 1 |
| FCGRT | 0.000569 | 0.383528 | 0.853 | 0.596 | 1 |
| RNASE1 | 0.00057 | -0.5255 | 0.147 | 0.457 | 1 |
| ATP6V0B | 0.00057 | -0.36632 | 0.853 | 0.867 | 1 |
| HSP90AA1 | 0.000576 | 0.762662 | 1 | 0.989 | 1 |
| C3orf52 | 0.000583 | -0.29691 | 0.147 | 0.441 | 1 |
| TPPP3 | 0.000586 | 0.688718 | 0.676 | 0.516 | 1 |
| PIK3R3 | 0.000602 | 0.298407 | 0.706 | 0.447 | 1 |
| UQCRH | 0.000602 | -0.34576 | 0.912 | 0.883 | 1 |
| DPH3 | 0.00061 | -0.32086 | 0.353 | 0.617 | 1 |
| CDH1 | 0.000619 | -0.41811 | 0.824 | 0.878 | 1 |
| CTNND1 | 0.000626 | -0.32327 | 0.765 | 0.856 | 1 |
| CD99 | 0.000635 | -0.49926 | 0.706 | 0.798 | 1 |
| PERP | 0.000641 | -0.36159 | 0.941 | 0.968 | 1 |
| AGR3 | 0.000647 | 0.607657 | 0.794 | 0.723 | 1 |
| KCNQ1OT1 | 0.00065 | 0.276782 | 0.471 | 0.229 | 1 |
| NDFIP1 | 0.000652 | -0.37252 | 0.824 | 0.872 | 1 |
| AGR2 | 0.000654 | -0.567 | 0.941 | 0.931 | 1 |
| HES4 | 0.000659 | -0.45104 | 0.618 | 0.814 | 1 |
| GDE1 | 0.00067 | -0.30393 | 0.441 | 0.638 | 1 |
| GPT2 | 0.000672 | -0.25258 | 0.147 | 0.447 | 1 |
| DUSP10 | 0.000711 | -0.39189 | 0.118 | 0.404 | 1 |
| CD63 | 0.000724 | -0.29711 | 0.971 | 0.952 | 1 |
| MTSS1L | 0.000775 | 0.28678 | 0.588 | 0.367 | 1 |
| TMEM92 | 0.000783 | -0.26745 | 0.147 | 0.426 | 1 |
| TMA7 | 0.000783 | -0.32207 | 0.971 | 0.957 | 1 |
| AZIN1 | 0.0008 | 0.364974 | 0.794 | 0.681 | 1 |
| CLSTN1 | 0.000806 | 0.277328 | 0.882 | 0.734 | 1 |
| PTOV1 | 0.000826 | 0.352545 | 0.853 | 0.755 | 1 |
| TSC22D1 | 0.00083 | -0.5043 | 0.882 | 0.91 | 1 |
| TUSC3 | 0.000834 | 0.414804 | 0.706 | 0.537 | 1 |
| TMEM167A | 0.000838 | -0.27616 | 0.529 | 0.723 | 1 |
| MT-ND4L | 0.000843 | 0.842195 | 1 | 0.995 | 1 |
| ERLEC1 | 0.00085 | 0.293911 | 0.912 | 0.755 | 1 |
| RHOBTB3 | 0.000895 | -0.26461 | 0.294 | 0.569 | 1 |
| LENG8 | 0.0009 | 0.302406 | 0.882 | 0.739 | 1 |
| CFL2 | 0.00091 | -0.29741 | 0.147 | 0.436 | 1 |
| DNAJC1 | 0.000923 | -0.28382 | 0.529 | 0.691 | 1 |
| UBC | 0.000926 | -0.47759 | 1 | 0.979 | 1 |
| LCOR | 0.000928 | 0.311081 | 0.853 | 0.633 | 1 |
| FAM173A | 0.00094 | -0.31457 | 0.206 | 0.484 | 1 |
| RHOG | 0.000942 | -0.28035 | 0.265 | 0.585 | 1 |
| ERCC1 | 0.00095 | 0.260598 | 0.706 | 0.548 | 1 |
| CEP350 | 0.000953 | 0.299794 | 0.824 | 0.559 | 1 |
| NAA38 | 0.000956 | 0.278003 | 0.912 | 0.856 | 1 |
| IFRD1 | 0.000956 | -0.34571 | 0.412 | 0.622 | 1 |
| TBC1D8 | 0.000977 | 0.270122 | 0.588 | 0.34 | 1 |
| MLF1 | 0.000995 | 0.362869 | 0.647 | 0.447 | 1 |
| TSPO | 0.000999 | -0.32089 | 0.941 | 0.883 | 1 |
| HDLBP | 0.001 | 0.285863 | 0.971 | 0.824 | 1 |
| GSN | 0.00101 | -0.39718 | 0.971 | 0.941 | 1 |
| NEBL | 0.001025 | 0.300553 | 0.765 | 0.59 | 1 |
| AKR1C3 | 0.00103 | 0.305864 | 0.765 | 0.532 | 1 |
| PSMB10 | 0.001037 | 0.276099 | 0.941 | 0.75 | 1 |
| RPL19 | 0.001038 | -0.29397 | 1 | 0.989 | 1 |
| NGRN | 0.001045 | 0.315211 | 0.824 | 0.67 | 1 |
| BTG3 | 0.001075 | -0.38085 | 0.618 | 0.691 | 1 |
| RAB10 | 0.001097 | -0.33213 | 0.794 | 0.819 | 1 |
| TSPAN3 | 0.001103 | -0.30809 | 0.912 | 0.957 | 1 |
| C1QB | 0.001109 | 1.347617 | 0.265 | 0.074 | 1 |
| PAWR | 0.001119 | -0.31101 | 0.471 | 0.676 | 1 |
| RPS8 | 0.001173 | -0.35855 | 0.971 | 1 | 1 |
| ACTR3 | 0.001175 | -0.40947 | 0.735 | 0.856 | 1 |
| SERPINF1 | 0.001177 | 0.25972 | 0.794 | 0.527 | 1 |
| ENAH | 0.001211 | 0.297871 | 0.765 | 0.585 | 1 |
| TRA2B | 0.001215 | -0.33039 | 0.647 | 0.777 | 1 |
| RDH10 | 0.001216 | -0.41345 | 0.735 | 0.83 | 1 |
| KMT2C | 0.001216 | 0.28999 | 0.824 | 0.596 | 1 |
| DNASE1L1 | 0.001227 | 0.254537 | 0.382 | 0.16 | 1 |
| RAP1B | 0.00123 | -0.33908 | 0.765 | 0.851 | 1 |
| TPM3 | 0.001243 | -0.27109 | 0.882 | 0.926 | 1 |
| MKL2 | 0.001246 | 0.265846 | 0.647 | 0.378 | 1 |
| NDUFAF3 | 0.001254 | 0.276145 | 0.824 | 0.718 | 1 |
| TRIP10 | 0.001254 | -0.2695 | 0.324 | 0.564 | 1 |
| AUP1 | 0.001263 | -0.28746 | 0.647 | 0.793 | 1 |
| PPP4R3B | 0.001274 | 0.321733 | 0.765 | 0.559 | 1 |
| KDM5A | 0.001286 | 0.386062 | 0.706 | 0.441 | 1 |
| FAIM | 0.001292 | 0.267279 | 0.618 | 0.372 | 1 |
| STX3 | 0.001295 | -0.31128 | 0.353 | 0.59 | 1 |
| AVPI1 | 0.001295 | -0.3239 | 0.353 | 0.601 | 1 |
| MCU | 0.001302 | -0.27968 | 0.353 | 0.606 | 1 |
| DAAM1 | 0.001309 | 0.264836 | 0.765 | 0.484 | 1 |
| BST2 | 0.001313 | -0.44971 | 0.853 | 0.92 | 1 |
| B3GNT5 | 0.001327 | 0.258241 | 0.794 | 0.59 | 1 |
| TLNRD1 | 0.001347 | -0.3048 | 0.294 | 0.537 | 1 |
| EIF3H | 0.00136 | -0.31372 | 0.706 | 0.761 | 1 |
| MESP1 | 0.001366 | -0.28061 | 0.088 | 0.362 | 1 |
| SDCCAG8 | 0.001376 | 0.250791 | 0.735 | 0.484 | 1 |
| ELF3 | 0.001379 | -0.33187 | 0.971 | 0.957 | 1 |
| SPCS1 | 0.001395 | 0.284804 | 0.971 | 0.899 | 1 |
| EFHD2 | 0.0014 | -0.3189 | 0.706 | 0.798 | 1 |
| HMOX1 | 0.001417 | -0.44668 | 0.088 | 0.362 | 1 |
| MIDN | 0.001428 | -0.39407 | 0.912 | 0.867 | 1 |
| MISP | 0.001448 | -0.3679 | 0.824 | 0.846 | 1 |
| ERICH1 | 0.001449 | 0.305538 | 0.647 | 0.356 | 1 |
| ZNF185 | 0.001449 | -0.31082 | 0.265 | 0.521 | 1 |
| ELK3 | 0.001474 | 0.296049 | 0.647 | 0.441 | 1 |
| LAMTOR5 | 0.001507 | -0.32735 | 0.765 | 0.888 | 1 |
| CRNDE | 0.001521 | 0.408779 | 0.794 | 0.729 | 1 |
| MYD88 | 0.001526 | -0.35776 | 0.618 | 0.718 | 1 |
| MBD2 | 0.001539 | -0.25335 | 0.471 | 0.691 | 1 |
| LRRFIP2 | 0.001562 | -0.32578 | 0.5 | 0.686 | 1 |
| MYOF | 0.001572 | -0.37495 | 0.794 | 0.809 | 1 |
| ITPKC | 0.00158 | -0.31226 | 0.412 | 0.617 | 1 |
| PTGES3 | 0.001581 | 0.324331 | 0.941 | 0.904 | 1 |
| RPS29 | 0.001587 | -0.33878 | 1 | 0.973 | 1 |
| PPP2CA | 0.001595 | -0.26149 | 0.618 | 0.734 | 1 |
| ZNF706 | 0.001607 | -0.30007 | 0.824 | 0.899 | 1 |
| STK17B | 0.001614 | -0.30117 | 0.324 | 0.543 | 1 |
| EIF2AK1 | 0.00163 | 0.250878 | 0.735 | 0.516 | 1 |
| RALA | 0.001632 | -0.29109 | 0.559 | 0.697 | 1 |
| FAM120AOS | 0.001647 | -0.28108 | 0.382 | 0.601 | 1 |
| RPL22 | 0.001659 | -0.3282 | 0.971 | 0.952 | 1 |
| S100A13 | 0.001684 | -0.3724 | 0.912 | 0.899 | 1 |
| KRT80 | 0.001695 | -0.48006 | 0.324 | 0.553 | 1 |
| NOP10 | 0.00173 | -0.34777 | 0.882 | 0.92 | 1 |
| DGKH | 0.001734 | 0.30519 | 0.706 | 0.516 | 1 |
| MRPS12 | 0.001752 | -0.2731 | 0.353 | 0.58 | 1 |
| ARPC5L | 0.001791 | -0.32 | 0.794 | 0.846 | 1 |
| ELOVL1 | 0.001801 | -0.25389 | 0.353 | 0.553 | 1 |
| IFITM1 | 0.001805 | -0.58166 | 0.941 | 0.979 | 1 |
| SPCS3 | 0.001814 | -0.31436 | 0.5 | 0.718 | 1 |
| KLK10 | 0.001815 | -0.67776 | 0.412 | 0.681 | 1 |
| DIAPH1 | 0.001831 | -0.35382 | 0.647 | 0.718 | 1 |
| SECTM1 | 0.001843 | -0.31077 | 0.235 | 0.511 | 1 |
| RPS14 | 0.00185 | -0.34535 | 0.971 | 0.984 | 1 |
| SH3GLB1 | 0.001869 | -0.34879 | 0.824 | 0.835 | 1 |
| OCLN | 0.001888 | -0.41939 | 0.559 | 0.707 | 1 |
| BPTF | 0.001905 | 0.286199 | 0.912 | 0.745 | 1 |
| PART1 | 0.001933 | 0.309618 | 0.618 | 0.404 | 1 |
| WHAMM | 0.001938 | -0.25426 | 0.206 | 0.452 | 1 |
| SRI | 0.001946 | 0.391041 | 0.912 | 0.915 | 1 |
| ATP5F1D | 0.001957 | -0.36494 | 0.882 | 0.888 | 1 |
| KYNU | 0.00197 | -0.28831 | 0.147 | 0.415 | 1 |
| MPHOSPH8 | 0.002025 | 0.309614 | 0.853 | 0.574 | 1 |
| CD46 | 0.002048 | 0.262913 | 0.853 | 0.734 | 1 |
| HSPB11 | 0.002073 | 0.299807 | 0.853 | 0.665 | 1 |
| ATP8B1 | 0.002087 | 0.259145 | 0.794 | 0.5 | 1 |
| AL138963.3 | 0.00209 | -0.5219 | 0.176 | 0.447 | 1 |
| TP53TG1 | 0.002128 | 0.337144 | 0.765 | 0.628 | 1 |
| SERPINH1 | 0.00215 | -0.26448 | 0.118 | 0.362 | 1 |
| RPL23A | 0.002152 | -0.36273 | 0.941 | 0.968 | 1 |
| PODXL | 0.002162 | -0.32395 | 0.529 | 0.686 | 1 |
| PTP4A1 | 0.002216 | -0.28284 | 0.618 | 0.745 | 1 |
| SRF | 0.002222 | -0.28842 | 0.206 | 0.479 | 1 |
| GTF2I | 0.002265 | 0.279799 | 0.941 | 0.878 | 1 |
| HK2 | 0.002284 | -0.25332 | 0.176 | 0.441 | 1 |
| INSR | 0.002308 | 0.367098 | 0.765 | 0.495 | 1 |
| TRIP11 | 0.002308 | 0.294967 | 0.765 | 0.606 | 1 |
| ARHGEF28 | 0.002383 | 0.272714 | 0.618 | 0.378 | 1 |
| RPL7A | 0.002394 | -0.40959 | 0.971 | 0.979 | 1 |
| CFAP97 | 0.002411 | 0.287087 | 0.735 | 0.479 | 1 |
| HSPBP1 | 0.002412 | 0.490984 | 0.676 | 0.585 | 1 |
| UBE2B | 0.00244 | -0.39569 | 0.794 | 0.814 | 1 |
| AKR1C2 | 0.002467 | -0.5951 | 0.471 | 0.633 | 1 |
| PRSS23 | 0.002484 | -0.48729 | 0.853 | 0.872 | 1 |
| ISG15 | 0.002512 | -0.59631 | 1 | 0.968 | 1 |
| SEC61G | 0.002689 | -0.35227 | 0.912 | 0.91 | 1 |
| FAM221A | 0.002697 | 0.286003 | 0.647 | 0.457 | 1 |
| GFPT1 | 0.002739 | 0.284023 | 0.853 | 0.686 | 1 |
| RPL6 | 0.002789 | -0.34456 | 1 | 0.952 | 1 |
| LGALS9 | 0.002878 | -0.34866 | 0.765 | 0.814 | 1 |
| FCGBP | 0.002918 | 0.56647 | 0.382 | 0.191 | 1 |
| HIST1H2BC | 0.002919 | -0.39571 | 0.324 | 0.532 | 1 |
| 5-Mar | 0.002937 | -0.33785 | 0.5 | 0.617 | 1 |
| CALM2 | 0.003009 | 0.296636 | 0.971 | 0.979 | 1 |
| SLC25A1 | 0.00303 | -0.25355 | 0.353 | 0.58 | 1 |
| CRYM | 0.003073 | 0.387998 | 0.441 | 0.255 | 1 |
| MUC4 | 0.003107 | -0.36299 | 0.912 | 0.941 | 1 |
| CELSR1 | 0.003156 | 0.273549 | 0.765 | 0.596 | 1 |
| TNFRSF1A | 0.003303 | -0.27716 | 0.588 | 0.745 | 1 |
| RAB1A | 0.003408 | -0.33558 | 0.765 | 0.835 | 1 |
| BRI3 | 0.003463 | -0.2852 | 0.676 | 0.856 | 1 |
| FAU | 0.003464 | -0.27159 | 1 | 0.989 | 1 |
| PPP1CA | 0.003491 | -0.328 | 0.853 | 0.809 | 1 |
| ST13 | 0.00359 | 0.259739 | 0.971 | 0.926 | 1 |
| SEC11C | 0.003607 | -0.31683 | 0.706 | 0.707 | 1 |
| CALM1 | 0.003646 | 0.479121 | 0.971 | 0.984 | 1 |
| OAS1 | 0.003742 | -0.53009 | 0.912 | 0.931 | 1 |
| SLC25A6 | 0.003758 | -0.34314 | 0.882 | 0.862 | 1 |
| RRAGC | 0.003827 | -0.26605 | 0.235 | 0.495 | 1 |
| ATRAID | 0.003858 | 0.255352 | 0.794 | 0.638 | 1 |
| CTNNBIP1 | 0.003867 | -0.25073 | 0.353 | 0.559 | 1 |
| C1QC | 0.003919 | 1.376078 | 0.324 | 0.122 | 1 |
| TMEM123 | 0.003994 | 0.250686 | 0.941 | 0.835 | 1 |
| KDELR2 | 0.004012 | -0.27642 | 0.853 | 0.787 | 1 |
| RPL13 | 0.004017 | -0.28316 | 1 | 0.995 | 1 |
| PLAC8 | 0.004026 | -0.8122 | 0.853 | 0.931 | 1 |
| RACK1 | 0.004166 | -0.34197 | 0.941 | 0.968 | 1 |
| SMARCA5 | 0.004168 | 0.260436 | 0.824 | 0.649 | 1 |
| NSMCE1 | 0.004226 | 0.275828 | 0.647 | 0.468 | 1 |
| TRIP6 | 0.004234 | -0.29987 | 0.588 | 0.697 | 1 |
| SCNN1A | 0.00424 | -0.26542 | 0.765 | 0.803 | 1 |
| ARF4 | 0.004351 | -0.28231 | 0.912 | 0.878 | 1 |
| PLK2 | 0.004373 | -0.27497 | 0.324 | 0.527 | 1 |
| WDR13 | 0.004438 | 0.262158 | 0.676 | 0.585 | 1 |
| MGAT1 | 0.004596 | -0.276 | 0.471 | 0.676 | 1 |
| MBOAT2 | 0.004604 | -0.25397 | 0.324 | 0.58 | 1 |
| RPS27 | 0.004628 | -0.27738 | 0.971 | 0.995 | 1 |
| C12orf57 | 0.004634 | -0.29889 | 0.676 | 0.846 | 1 |
| HNRNPDL | 0.004676 | -0.29672 | 0.941 | 0.904 | 1 |
| VMO1 | 0.004722 | -0.56793 | 0.647 | 0.761 | 1 |
| CYP2S1 | 0.004737 | -0.27409 | 0.265 | 0.484 | 1 |
| CCT2 | 0.00484 | 0.287842 | 0.853 | 0.665 | 1 |
| DYNLL1 | 0.004849 | 0.603094 | 0.824 | 0.926 | 1 |
| TM7SF2 | 0.004968 | 0.257444 | 0.706 | 0.42 | 1 |
| TMF1 | 0.005036 | 0.25224 | 0.794 | 0.729 | 1 |
| GALNT12 | 0.005042 | -0.27727 | 0.353 | 0.564 | 1 |
| NAA50 | 0.005139 | -0.28055 | 0.559 | 0.702 | 1 |
| BCL10 | 0.00524 | -0.25395 | 0.412 | 0.622 | 1 |
| SYAP1 | 0.005255 | 0.259241 | 0.794 | 0.676 | 1 |
| SAMD9 | 0.005361 | -0.3514 | 0.882 | 0.862 | 1 |
| MTCH1 | 0.005383 | -0.25031 | 0.882 | 0.84 | 1 |
| PRELID1 | 0.005501 | -0.31485 | 0.794 | 0.824 | 1 |
| BZW1 | 0.005581 | -0.26971 | 0.794 | 0.878 | 1 |
| EHF | 0.005592 | 0.30877 | 0.941 | 0.681 | 1 |
| CWF19L2 | 0.005605 | 0.258364 | 0.412 | 0.197 | 1 |
| SLC25A5 | 0.005672 | -0.50094 | 0.971 | 0.862 | 1 |
| SH3GL1 | 0.00568 | -0.27774 | 0.382 | 0.617 | 1 |
| TMEM14B | 0.005689 | 0.254463 | 0.912 | 0.824 | 1 |
| CTSL | 0.00574 | -0.56609 | 0.706 | 0.436 | 1 |
| SNHG12 | 0.005754 | -0.38418 | 0.471 | 0.617 | 1 |
| HSBP1L1 | 0.00581 | -0.28091 | 0.618 | 0.713 | 1 |
| GNAI3 | 0.005811 | -0.2528 | 0.5 | 0.617 | 1 |
| UBE2I | 0.006075 | -0.2505 | 0.735 | 0.75 | 1 |
| SNRPD2 | 0.006203 | -0.27866 | 0.882 | 0.883 | 1 |
| GUK1 | 0.006263 | -0.257 | 0.912 | 0.931 | 1 |
| MAFK | 0.006474 | -0.32905 | 0.412 | 0.574 | 1 |
| BUD23 | 0.006535 | 0.252043 | 0.824 | 0.718 | 1 |
| ID3 | 0.00654 | -0.25365 | 0.118 | 0.319 | 1 |
| WDTC1 | 0.006561 | 0.259539 | 0.559 | 0.426 | 1 |
| IFIT3 | 0.006575 | -0.6184 | 0.971 | 0.926 | 1 |
| PHF11 | 0.006639 | -0.30585 | 0.676 | 0.761 | 1 |
| MDK | 0.0068 | -0.38705 | 0.971 | 0.904 | 1 |
| FRMD4B | 0.006803 | -0.26939 | 0.324 | 0.532 | 1 |
| UBE2J1 | 0.006838 | -0.25109 | 0.529 | 0.676 | 1 |
| PPDPF | 0.006996 | -0.36861 | 0.941 | 0.957 | 1 |
| MT-CO1 | 0.006998 | 0.522582 | 1 | 1 | 1 |
| HDAC7 | 0.007067 | -0.28652 | 0.441 | 0.633 | 1 |
| SOD1 | 0.00717 | 0.332681 | 0.912 | 0.926 | 1 |
| RAB5A | 0.007198 | -0.26967 | 0.471 | 0.665 | 1 |
| SLC4A11 | 0.007277 | -0.27664 | 0.5 | 0.681 | 1 |
| SLC16A3 | 0.007423 | -0.31631 | 0.618 | 0.691 | 1 |
| FTH1 | 0.007437 | -0.32127 | 1 | 0.995 | 1 |
| RPL5 | 0.00763 | -0.38952 | 1 | 0.952 | 1 |
| UBXN4 | 0.007669 | 0.255549 | 0.941 | 0.894 | 1 |
| ADH1C | 0.007703 | 0.404382 | 0.824 | 0.612 | 1 |
| IRF7 | 0.007875 | -0.50406 | 0.882 | 0.851 | 1 |
| TIMM8B | 0.007878 | -0.30869 | 0.735 | 0.819 | 1 |
| SLC2A1 | 0.007973 | -0.3145 | 0.471 | 0.628 | 1 |
| CFAP36 | 0.007973 | 0.313778 | 0.588 | 0.463 | 1 |
| PLSCR1 | 0.008097 | -0.36337 | 0.971 | 0.926 | 1 |
| KIF5B | 0.008399 | 0.293127 | 0.971 | 0.835 | 1 |
| RBMS2 | 0.008475 | -0.27194 | 0.353 | 0.553 | 1 |
| SULT2B1 | 0.008635 | -0.27864 | 0.265 | 0.5 | 1 |
| MAFB | 0.008639 | -0.33055 | 0.088 | 0.298 | 1 |
| TM2D2 | 0.008708 | -0.2756 | 0.441 | 0.638 | 1 |
| CLDND1 | 0.00876 | -0.27754 | 0.412 | 0.564 | 1 |
| BBIP1 | 0.008776 | 0.251872 | 0.647 | 0.479 | 1 |
| PDLIM7 | 0.008796 | -0.26015 | 0.147 | 0.351 | 1 |
| OASL | 0.008827 | -0.51176 | 0.529 | 0.676 | 1 |
| MRPS6 | 0.009021 | -0.35665 | 0.706 | 0.782 | 1 |
| KDM6B | 0.009053 | -0.34376 | 0.265 | 0.479 | 1 |
| TOP1 | 0.009103 | -0.2865 | 0.853 | 0.878 | 1 |
| WASL | 0.009251 | -0.31184 | 0.794 | 0.793 | 1 |
| RPSA | 0.009448 | -0.26648 | 1 | 0.973 | 1 |
| PPIB | 0.010065 | -0.28929 | 0.941 | 0.888 | 1 |
| DUSP6 | 0.010203 | -0.36489 | 0.088 | 0.293 | 1 |
| ARRDC2 | 0.010293 | -0.26762 | 0.206 | 0.436 | 1 |
| MYO5B | 0.010572 | -0.28778 | 0.588 | 0.734 | 1 |
| TUBA1C | 0.010954 | -0.34377 | 0.735 | 0.739 | 1 |
| ZBTB43 | 0.010977 | -0.28439 | 0.382 | 0.569 | 1 |
| IFITM3 | 0.011077 | -0.32544 | 1 | 0.973 | 1 |
| TRIM29 | 0.01108 | -0.31859 | 0.765 | 0.819 | 1 |
| RBMS1 | 0.011345 | -0.33919 | 0.735 | 0.771 | 1 |
| AC009133.1 | 0.011431 | -0.26038 | 0.265 | 0.463 | 1 |
| CEBPB | 0.0116 | -0.262 | 0.765 | 0.867 | 1 |
| HDAC9 | 0.01191 | -0.28434 | 0.206 | 0.394 | 1 |
| FAM84A | 0.012028 | -0.38732 | 0.5 | 0.633 | 1 |
| ICAM1 | 0.012062 | -0.43622 | 0.088 | 0.282 | 1 |
| NCOA7 | 0.012105 | -0.55243 | 0.765 | 0.771 | 1 |
| AMD1 | 0.012107 | -0.25939 | 0.618 | 0.66 | 1 |
| GCLC | 0.012338 | -0.40329 | 0.559 | 0.723 | 1 |
| ENO1 | 0.012474 | -0.44034 | 0.824 | 0.793 | 1 |
| PTPN2 | 0.012772 | -0.28073 | 0.588 | 0.676 | 1 |
| VMP1 | 0.012882 | -0.39492 | 0.794 | 0.835 | 1 |
| BTG2 | 0.013043 | -0.37404 | 0.441 | 0.58 | 1 |
| XBP1 | 0.013267 | 0.431774 | 0.971 | 0.824 | 1 |
| MUC5B | 0.013269 | 1.523275 | 0.441 | 0.324 | 1 |
| MUC5AC | 0.013509 | -0.2794 | 0.235 | 0.463 | 1 |
| IRX2 | 0.014034 | 0.272356 | 0.5 | 0.271 | 1 |
| MED15 | 0.014038 | -0.26378 | 0.5 | 0.676 | 1 |
| MYDGF | 0.01415 | -0.25252 | 0.794 | 0.809 | 1 |
| EIF3A | 0.014313 | 0.283272 | 0.912 | 0.798 | 1 |
| RRBP1 | 0.014782 | 0.257698 | 0.912 | 0.83 | 1 |
| SPATS2L | 0.014844 | -0.39084 | 0.971 | 0.926 | 1 |
| TRAF4 | 0.015066 | -0.26865 | 0.853 | 0.819 | 1 |
| TMEM50A | 0.015156 | -0.30899 | 0.912 | 0.856 | 1 |
| SDHD | 0.0153 | -0.26269 | 0.471 | 0.596 | 1 |
| CDK2AP2 | 0.015524 | -0.38654 | 0.706 | 0.707 | 1 |
| YWHAH | 0.016167 | -0.39072 | 0.765 | 0.83 | 1 |
| EIF5B | 0.016327 | 0.255349 | 0.882 | 0.766 | 1 |
| HNRNPA1 | 0.016457 | -0.25869 | 0.941 | 0.92 | 1 |
| PICALM | 0.016733 | -0.30934 | 0.529 | 0.617 | 1 |
| FKBP4 | 0.018045 | -0.37082 | 0.735 | 0.723 | 1 |
| DRAP1 | 0.018367 | -0.31011 | 0.912 | 0.824 | 1 |
| TRAM1 | 0.018747 | -0.42216 | 0.765 | 0.798 | 1 |
| CGN | 0.018802 | 0.305167 | 0.706 | 0.606 | 1 |
| PPP2R2A | 0.018907 | -0.26452 | 0.706 | 0.771 | 1 |
| IFI44L | 0.019148 | 0.381598 | 0.971 | 0.888 | 1 |
| SAA1 | 0.019229 | 0.890646 | 0.647 | 0.516 | 1 |
| UBE2L6 | 0.01938 | -0.2766 | 0.912 | 0.904 | 1 |
| CCT5 | 0.019477 | 0.252975 | 0.765 | 0.702 | 1 |
| RAN | 0.019778 | -0.29742 | 0.882 | 0.878 | 1 |
| IL18 | 0.02009 | -0.52542 | 0.618 | 0.638 | 1 |
| COX5A | 0.020643 | -0.25486 | 0.853 | 0.936 | 1 |
| FABP5 | 0.020749 | -0.3477 | 0.471 | 0.638 | 1 |
| COX20 | 0.020795 | -0.2639 | 0.676 | 0.729 | 1 |
| RPS7 | 0.020829 | -0.27035 | 1 | 0.963 | 1 |
| CNN2 | 0.021798 | -0.28361 | 0.676 | 0.723 | 1 |
| RAP1A | 0.022193 | -0.25647 | 0.647 | 0.67 | 1 |
| EPSTI1 | 0.023156 | -0.27611 | 0.794 | 0.814 | 1 |
| TTC9 | 0.02316 | -0.31188 | 0.618 | 0.676 | 1 |
| MAX | 0.023834 | -0.25056 | 0.676 | 0.745 | 1 |
| PTMS | 0.023834 | -0.33477 | 0.647 | 0.75 | 1 |
| PABPN1 | 0.023901 | 0.356618 | 0.882 | 0.713 | 1 |
| FAM118A | 0.024026 | 0.292678 | 0.324 | 0.17 | 1 |
| ARID5B | 0.02403 | -0.29527 | 0.5 | 0.606 | 1 |
| PDP1 | 0.024605 | -0.26266 | 0.412 | 0.548 | 1 |
| RPL3 | 0.024627 | -0.27742 | 0.971 | 0.979 | 1 |
| OXR1 | 0.024692 | 0.253374 | 0.559 | 0.431 | 1 |
| SELENOW | 0.024996 | 0.289855 | 1 | 0.952 | 1 |
| TRIM16 | 0.025613 | -0.31343 | 0.529 | 0.585 | 1 |
| MUC20 | 0.026195 | -0.34701 | 0.853 | 0.878 | 1 |
| INSIG1 | 0.026891 | -0.37483 | 0.412 | 0.532 | 1 |
| RARRES3 | 0.027168 | -0.46449 | 0.971 | 0.888 | 1 |
| HLA-F | 0.027391 | 0.266334 | 0.882 | 0.793 | 1 |
| ABCF1 | 0.027483 | 0.252391 | 0.706 | 0.495 | 1 |
| LPIN1 | 0.027598 | -0.27353 | 0.294 | 0.452 | 1 |
| RGCC | 0.028472 | -0.46759 | 0.706 | 0.707 | 1 |
| SMS | 0.028639 | -0.33063 | 0.706 | 0.824 | 1 |
| CLIC5 | 0.028661 | -0.27142 | 0.176 | 0.324 | 1 |
| CALR | 0.028729 | -0.25903 | 0.971 | 0.894 | 1 |
| GABRP | 0.028996 | -0.28624 | 0.588 | 0.777 | 1 |
| SNHG7 | 0.029478 | -0.27858 | 0.647 | 0.654 | 1 |
| NAMPT | 0.029581 | -0.36986 | 0.706 | 0.809 | 1 |
| MTRNR2L8 | 0.029973 | -0.33996 | 0.735 | 0.777 | 1 |
| SMARCA4 | 0.030769 | 0.27142 | 0.824 | 0.612 | 1 |
| LITAF | 0.030983 | -0.30987 | 0.882 | 0.856 | 1 |
| YBX3 | 0.031334 | -0.26752 | 0.824 | 0.904 | 1 |
| TNFAIP3 | 0.031611 | -0.4185 | 0.471 | 0.622 | 1 |
| RNF149 | 0.031787 | -0.2671 | 0.559 | 0.676 | 1 |
| HS3ST1 | 0.032195 | -0.33929 | 0.765 | 0.787 | 1 |
| NFE2L2 | 0.032518 | 0.251285 | 0.941 | 0.894 | 1 |
| PLD3 | 0.033141 | 0.441221 | 0.765 | 0.606 | 1 |
| NCL | 0.0339 | 0.273083 | 0.912 | 0.846 | 1 |
| GLTP | 0.034249 | -0.29428 | 0.471 | 0.59 | 1 |
| PIM3 | 0.036865 | -0.31584 | 0.618 | 0.686 | 1 |
| ABHD2 | 0.041813 | -0.28792 | 0.794 | 0.846 | 1 |
| TENT5A | 0.042271 | -0.25126 | 0.647 | 0.723 | 1 |
| TJP1 | 0.042324 | -0.27693 | 0.765 | 0.771 | 1 |
| C9orf16 | 0.044774 | -0.26473 | 0.853 | 0.851 | 1 |
| RBM3 | 0.044861 | -0.28138 | 0.706 | 0.734 | 1 |
| SERPINB3 | 0.045597 | -0.36139 | 0.559 | 0.766 | 1 |
| SLC20A1 | 0.045932 | -0.2883 | 0.5 | 0.601 | 1 |
| SLC4A4 | 0.045985 | 0.416595 | 0.471 | 0.362 | 1 |
| MT-CO2 | 0.046068 | 0.319798 | 1 | 1 | 1 |
| RAB11FIP1 | 0.048942 | -0.33759 | 0.912 | 0.926 | 1 |
| NQO1 | 0.050226 | 0.349418 | 0.735 | 0.691 | 1 |
| SORD | 0.051861 | 0.283577 | 0.735 | 0.612 | 1 |
| CSRP1 | 0.05205 | -0.32158 | 0.559 | 0.628 | 1 |
| CLDN23 | 0.054321 | -0.34956 | 0.588 | 0.649 | 1 |
| RASEF | 0.05443 | -0.25622 | 0.588 | 0.691 | 1 |
| GALNT3 | 0.054652 | -0.25304 | 0.647 | 0.798 | 1 |
| ZFAND5 | 0.056652 | -0.30933 | 0.882 | 0.83 | 1 |
| FOLR1 | 0.057982 | 0.315807 | 0.324 | 0.218 | 1 |
| CARHSP1 | 0.058898 | -0.31658 | 0.706 | 0.75 | 1 |
| MYH9 | 0.061327 | -0.38199 | 0.794 | 0.824 | 1 |
| TGIF1 | 0.062485 | -0.28286 | 0.676 | 0.729 | 1 |
| WFDC2 | 0.063726 | -0.44211 | 0.971 | 0.963 | 1 |
| PIGX | 0.065274 | 0.335173 | 0.765 | 0.601 | 1 |
| HRASLS2 | 0.066005 | -1.00552 | 0.706 | 0.755 | 1 |
| ADGRF1 | 0.067037 | -0.39884 | 0.882 | 0.867 | 1 |
| SRD5A3 | 0.068878 | -0.28553 | 0.588 | 0.617 | 1 |
| WEE1 | 0.074454 | -0.33857 | 0.559 | 0.58 | 1 |
| DDX21 | 0.079625 | -0.25091 | 0.735 | 0.702 | 1 |
| WARS | 0.082725 | -0.53946 | 0.882 | 0.819 | 1 |
| DDIT3 | 0.085859 | -0.51584 | 0.382 | 0.479 | 1 |
| OGFR | 0.086535 | -0.26459 | 0.588 | 0.628 | 1 |
| KLHDC7B | 0.088159 | -0.31312 | 0.265 | 0.394 | 1 |
| VAMP5 | 0.091157 | -0.35234 | 0.529 | 0.606 | 1 |
| LBH | 0.092576 | -0.26026 | 0.324 | 0.41 | 1 |
| UNC5B-AS1 | 0.094568 | -0.30037 | 0.441 | 0.516 | 1 |
| SERPINA1 | 0.094627 | 0.393285 | 0.353 | 0.207 | 1 |
| CCNO | 0.095847 | -0.25105 | 0.765 | 0.527 | 1 |
| TXNRD1 | 0.100226 | -0.32815 | 0.676 | 0.814 | 1 |
| NPM1 | 0.103643 | -0.25481 | 0.882 | 0.888 | 1 |
| CKB | 0.105736 | 0.299859 | 0.676 | 0.739 | 1 |
| MSMB | 0.1118 | -0.3692 | 0.559 | 0.633 | 1 |
| GPI | 0.115131 | -0.27219 | 0.735 | 0.691 | 1 |
| HES1 | 0.118655 | -0.43793 | 0.618 | 0.617 | 1 |
| RPS27L | 0.120037 | -0.29353 | 0.853 | 0.856 | 1 |
| STXBP2 | 0.122987 | 0.374353 | 0.706 | 0.612 | 1 |
| SLC20A2 | 0.131153 | -0.35635 | 0.647 | 0.622 | 1 |
| CDCP1 | 0.13425 | -0.30171 | 0.559 | 0.574 | 1 |
| XAF1 | 0.13468 | 0.351913 | 0.971 | 0.931 | 1 |
| CEBPD | 0.149219 | -0.3698 | 0.765 | 0.755 | 1 |
| CLDN3 | 0.159833 | -0.59213 | 0.794 | 0.782 | 1 |
| SCARB2 | 0.164049 | -0.26284 | 0.824 | 0.835 | 1 |
| PAQR4 | 0.168834 | -0.26341 | 0.294 | 0.346 | 1 |
| IFIT2 | 0.173389 | -0.42255 | 0.853 | 0.798 | 1 |
| LAG3 | 0.174712 | 0.349598 | 0.294 | 0.207 | 1 |
| MUC13 | 0.184491 | -0.33647 | 0.441 | 0.543 | 1 |
| MAP1LC3B | 0.185656 | -0.32504 | 0.824 | 0.803 | 1 |
| GBP1 | 0.198121 | -0.41577 | 0.794 | 0.771 | 1 |
| BPIFB1 | 0.213276 | -0.47933 | 0.706 | 0.755 | 1 |
| ITGA2 | 0.2134 | -0.29762 | 0.735 | 0.739 | 1 |
| DUOX2 | 0.236163 | -0.26254 | 0.382 | 0.42 | 1 |
| H2AFZ | 0.237952 | -0.34218 | 0.853 | 0.846 | 1 |
| HSPA8 | 0.240507 | 0.287696 | 0.912 | 0.957 | 1 |
| H1F0 | 0.24488 | -0.58626 | 0.618 | 0.654 | 1 |
| HIST1H1C | 0.327936 | -0.52206 | 0.706 | 0.697 | 1 |
| SLC5A3 | 0.403572 | -0.29152 | 0.294 | 0.356 | 1 |
| IFIT1 | 0.432397 | -0.58582 | 0.971 | 0.904 | 1 |
| SCGB3A1 | 0.484257 | 0.566541 | 0.794 | 0.793 | 1 |
| DDX58 | 0.527271 | -0.28418 | 0.735 | 0.697 | 1 |
| SERPING1 | 0.529647 | 0.382027 | 0.706 | 0.628 | 1 |
| ECE1 | 0.61263 | 0.2636 | 0.706 | 0.718 | 1 |
| H1FX | 0.625555 | -0.32253 | 0.765 | 0.628 | 1 |
| C3 | 0.693269 | 0.453922 | 0.647 | 0.676 | 1 |
| BPIFA1 | 0.749461 | -0.96935 | 0.441 | 0.463 | 1 |
| SCGB1A1 | 0.751263 | 0.61255 | 0.735 | 0.766 | 1 |
| TYROBP | 0.766697 | 0.519076 | 0.265 | 0.261 | 1 |
| CTSC | 0.924559 | 0.366492 | 0.706 | 0.814 | 1 |
| CTGF | 0.960382 | -0.35388 | 0.588 | 0.585 | 1 |
