# Supplementary material for: Single‐cell RNA sequencing identify SDCBP in ACE2‐positive bronchial epithelial cells negatively correlates with COVID‐19 severity
Source: J Cell Mol Med. 2021 Jun 16;25(14):7001–12. doi: 10.1111/jcmm.16714 (PMC8278084; doi:10.1111/jcmm.16714)
Supplement: Supplementary file 7 — Table S4 [file JCMM-25-7001-s010.docx]

| substanceBXH | ye | GS.tissue | p.GS.tissue |
| --- | --- | --- | --- |
| HLA-DRB5 | yellow | 0.51834 | 8.41E-17 |
| CD74 | yellow | 0.483135 | 1.67E-14 |
| HLA-DRA | yellow | 0.477268 | 3.82E-14 |
| HLA-DPB1 | yellow | 0.44293 | 3.52E-12 |
| HLA-DRB1 | yellow | 0.429694 | 1.77E-11 |
| HLA-DQA2 | yellow | 0.407853 | 2.18E-10 |
| HLA-DQB1 | yellow | 0.405226 | 2.91E-10 |
| HLA-DPA1 | yellow | 0.404546 | 3.14E-10 |
| DSE | yellow | 0.339981 | 1.83E-07 |
| BHLHE41 | yellow | 0.287391 | 1.24E-05 |
| HLA-DQA1 | yellow | 0.283083 | 1.70E-05 |
| FCGRT | yellow | 0.274673 | 3.07E-05 |
| CYP27A1 | yellow | 0.263559 | 6.51E-05 |
| APOE | yellow | 0.22363 | 0.000749 |
| C1QB | yellow | 0.217214 | 0.001068 |
| STXBP2 | yellow | 0.211969 | 0.001418 |
| PLD3 | yellow | 0.206973 | 0.001845 |
| APOC1 | yellow | 0.206234 | 0.001917 |
| SERPINA1 | yellow | 0.201388 | 0.002459 |
| SERPING1 | yellow | 0.19991 | 0.00265 |
| C1QC | yellow | 0.194336 | 0.003498 |
| ADA2 | yellow | 0.187842 | 0.004791 |
| LYZ | yellow | 0.17925 | 0.007154 |
| VIM | yellow | 0.17216 | 0.009836 |
| TXNIP | yellow | 0.171541 | 0.010108 |
| FABP4 | yellow | 0.169069 | 0.01126 |
| CD14 | yellow | 0.15681 | 0.018858 |
| C1orf162 | yellow | 0.154947 | 0.020336 |
| DDAH2 | yellow | 0.153564 | 0.021497 |
| GRB2 | yellow | 0.15217 | 0.022726 |
| C1QA | yellow | 0.149463 | 0.025285 |
| CCDC88A | yellow | 0.135675 | 0.042496 |
| CD52 | yellow | 0.135295 | 0.043084 |
| PSAP | yellow | 0.133788 | 0.045484 |
| LGALS1 | yellow | 0.132236 | 0.048071 |
| CCL18 | yellow | 0.130522 | 0.051069 |
| SOD2 | yellow | 0.128571 | 0.054669 |
| MS4A7 | yellow | 0.125535 | 0.060689 |
| NAGK | yellow | 0.124593 | 0.062664 |
| TLN1 | yellow | 0.119462 | 0.07437 |
| TYROBP | yellow | 0.118086 | 0.077792 |
| LTA4H | yellow | 0.115198 | 0.085393 |
| IFI30 | yellow | 0.114922 | 0.086152 |
| LIPA | yellow | 0.114852 | 0.086344 |
| GCA | yellow | 0.114621 | 0.086981 |
| SRGN | yellow | -0.11339 | 0.090441 |
| IGF2R | yellow | 0.113176 | 0.091062 |
| TMSB4X | yellow | -0.11192 | 0.094722 |
| GSTO1 | yellow | 0.109384 | 0.102498 |
| MS4A6A | yellow | 0.108533 | 0.105218 |
| CTSZ | yellow | 0.108088 | 0.106659 |
| ACP5 | yellow | 0.107384 | 0.108973 |
| LAPTM5 | yellow | 0.100555 | 0.133523 |
| CXCL10 | yellow | 0.100361 | 0.134277 |
| CTSB | yellow | -0.09886 | 0.140236 |
| MAFB | yellow | -0.09615 | 0.151502 |
| CCL2 | yellow | -0.09479 | 0.157391 |
| CD68 | yellow | 0.084427 | 0.208116 |
| PLEK | yellow | -0.08342 | 0.213604 |
| CCL3 | yellow | -0.08342 | 0.21362 |
| RNASET2 | yellow | 0.081304 | 0.225494 |
| CFD | yellow | 0.077609 | 0.247359 |
| HCST | yellow | 0.077085 | 0.250576 |
| SPI1 | yellow | 0.076987 | 0.251179 |
| AIF1 | yellow | -0.07681 | 0.252291 |
| CTSL | yellow | 0.076697 | 0.252975 |
| LGMN | yellow | -0.07643 | 0.25462 |
| BCL2A1 | yellow | -0.07395 | 0.270437 |
| CALHM6 | yellow | -0.06647 | 0.32203 |
| GRN | yellow | 0.06129 | 0.361228 |
| FCER1G | yellow | 0.058877 | 0.380473 |
| HEXB | yellow | 0.047729 | 0.477235 |
| FTH1 | yellow | 0.047678 | 0.477706 |
| FTL | yellow | 0.046706 | 0.486739 |
| VAT1 | yellow | 0.045303 | 0.499939 |
| GCH1 | yellow | -0.04113 | 0.54032 |
| CAPG | yellow | -0.03561 | 0.595972 |
| ANKRD1 | yellow | -0.03233 | 0.63036 |
| SDCBP | yellow | 0.02694 | 0.688402 |
| IFNGR2 | yellow | 0.021216 | 0.752163 |
| TNFSF13B | yellow | 0.017373 | 0.795962 |
| KIAA1551 | yellow | 0.007942 | 0.905908 |
| ACSL1 | yellow | -0.00037 | 0.995641 |
